# Teleworking as the main risk factor for sedentary behavior: the COVISTRESS international study

**DOI:** 10.1371/journal.pone.0356764

**Published:** 2026-09-10

**Authors:** Hijrah Nasir, Martine Duclos, Bruno Pereira, David Thivel, Jiao Jiao, Binh Quach, Reza Bagheri, Marek Zak, Ukadike Chris Ugbolue, Julien S. Baker, Alistair Cole, Martial Mermillod, Morteza Charkhabi, The COVISTRESS network, Maëlys Clinchamps, Frederic Dutheil

**Affiliations:** 1 Université Clermont Auvergne, Laboratory of the Metabolic Adaptations to Exercise under Physiological and Pathological Conditions (AME2P), CNRS LaPSCo, CHU Clermont-Ferrand, Clermont-Ferrand, France; 2 Université Clermont Auvergne, INRAE, UNH, CHU Clermont-Ferrand, Sport Medicine and Functional Exploration, Clermont-Ferrand, France; 3 CHU Clermont-Ferrand, Biostatistics, Clinical Research Direction, Clermont-Ferrand, France; 4 Université Clermont Auvergne, Laboratory of the Metabolic Adaptations to Exercise under Physiological and Pathological Conditions (AME2P), Clermont-Ferrand, France; 5 Hong Kong Baptist University, Sport and Physical Education, Hong KongChina; 6 University of Isfahan, Exercise Physiology, Isfahan, Iran; 7 The Jan Kochanowski University, Faculty of Medicine and Health Sciences, Institute of Physiotherapy, Kielce, Poland; 8 University of the West of Scotland, School of Health and Life Sciences, Institute for Clinical Exercise & Health Science, South Lanarkshire, Glasgow, United Kingdom; 9 Hong Kong Baptist University, Centre for Health and Exercise Science Research, Hong Kong, China; 10 Université Lumière - Lyon 2, SciencePo Lyon, Lyon, France; 11 Université Grenoble Alpes, Université Savoie Mont Blanc, CNRS, LPNC, 38000 Grenoble, France; 12 HSE University, Moscow, Russia; 13 Université Clermont Auvergne, CNRS, LaPSCo, Physiological and Psychosocial Stress, CHU Clermont-Ferrand, Occupational and Environmental Medicine, Clermont-Ferrand, France; China University of Mining and Technology, CHINA

## Abstract

**Background:**

If teleworking is already associated with prolonged sedentary behavior, the literature lacks an integrating model with all putative influencing variables, as well as a sufficient number of participants to explore extreme sitting behavior. Therefore, we aimed to investigate the influence of teleworking on sitting time, considering main sociodemographic, occupational and lifestyle factors.

**Methods:**

The COVISTRESS study is an international observational study, using self-reported questionnaires (REDCap® software). We used linear and logistic multivariate model to assess the impact of telework on sitting time concomitantly with other sociodemographic, occupational, and lifestyle variables.

**Results:**

6,653 respondents were included. The mean sitting time was 5.6 ± 3.4h/day, with 15.2% of respondents sitting for >8-14h/day and 1.6% sitting >14 hours/day. Workers who teleworked 0%, 1–25%, 26–50%, 51–75%, 76–99%, and 100% respectively sat on average 4.9 ± 3.2h/day, 5.8 ± 3.2h/day, 6.4 ± 3.1h/day, 6.5 ± 3.8h/day, 7.6 ± 3.5h/day, and 9.1 ± 4.3h/day, and the prevalence of workers sitting >14h/day increased from 1.5%, 1.9%, 0.4%, 3.2%, 5.4% to 10.7%. Compared with workers who did not telework, the risk of sitting 8–14 h/day was multiplied by 1.9 (1.2 to 3.0) for people working 1–25% in telework, by 4.7 (2.2 to 9.7) for people working 26–50% in telework, by 4.3 (1.5 to 12.2) for people working 51–75% in telework, and by 9.2 (1.6 to 51.5) for people working >75% in telework; the risk of sitting ≥ 14h/day was multiplied by 5.8 (1.07 to 31.5) for people working 51–75% in telework and dramatically increased by 34.3 (3.71 to 317) for people working >75% in telework in multivariate model.

**Conclusion:**

Teleworking is the strongest risk factor for high sitting time, with a marked dose-response relation. While several other sociodemographic, occupational, and lifestyle factors showed association with sitting time in univariate model, many of these relationships weakened or disappeared in the multivariate models, except telework that remained the most consistent determinants of sedentary behavior when concomitantly put together with all other variables.

**Trial registration:**

ClinicalTrials.gov NCT04538586

## Introduction

Prolonged sedentary behavior is associated with increased risks of all-cause mortality, cardiometabolic disease, cancer, and mental health disorders, independently of physical activity levels [1–4]. Sedentary behavior is defined as any waking behavior characterized by an energy expenditure ≤1.5 metabolic equivalents, while in a sitting, reclining, or lying posture [5]. Despite strong evidence of its detrimental effects, sitting time remains high worldwide [6]. Numerous sociodemographic, occupational, and lifestyle factors have been associated with sedentary behavior [7–10], including older age [10–13], women [10,13,14], being single [12,15,16] or living alone [13], higher education [14–16], living in the city vs rural [13], and lower household income [10], administrative workers and customer services occupations [17], work-related stress [18–22], smoking [23,24], alcohol consumption [23,25], cannabis use [25], obesity [13,15,24,26], and poor sleep [27,28]. Each of these factors contributes only partially to sedentary behavior, and their combined influence remains insufficiently explored.

A major recent occupational change concerns the rapid expansion of teleworking particularly since the COVID-19 pandemic [29–31]. The European Foundation defines teleworking as an arrangement in which employees carry out their job tasks away from the usual workplace, typically outside the employer’s premises, using information and communication technologies (ICT) [32]. Several studies and systematic reviews have reported substantial increases in sedentary time among teleworkers [15,33–41]. A systematic review showed between +6% and +67% increase in sedentary behavior during the COVID-19 pandemic [35], and thereafter [42], as well as shorter daily breaks during typical working hours [43]. Workers with the highest proportion of telework (76%−100% of working time) have been shown to accumulate the highest sedentary time (305 mins/day) compared with other groups (235.2 mins/day for those being 1%−25% in telework, 243.5 mins/day for 26%−50% in telework, and 258.5 mins/day for 51%‐75% in telework) [39]. However, most existing studies were conducted on a single country – three in Japan [11,12,21] and one in the Netherland [20] and only a minority used multivariate models adjusting simultaneously for sociodemographics, occupational, and lifestyle variables [11,12,20,21]. As a result, the independent contribution of telework relative to other determinants of sedentary behavior remains unclear. Furthermore, previous studies generally categorized sitting time using thresholds such as 8 h/day [44–48] or, more rarely, 11–12 h/day [49–53]. To our knowledge, no study has examined extreme sitting time, defined as >14 h/day, largely because available sample sizes were insufficient to capture such rare but clinically relevant behaviors. Identifying this subgroup is important, as extremely prolonged sitting may reflect distinct behavioral patterns and may carry disproportionately high health risks.

The COVISTRESS international study provides a unique opportunity to address this gap. Its sample size allows the simultaneous inclusion of all major sociodemographics, occupational, and lifestyle variables and the identification of extreme sedentary behavior (>14 h/day). For these reasons, sitting time was analyzed both as a continuous variable (hours/day) and as a categorical variable (≤3 h/day, >3–8 h/day, >8–14 h/day, and >14 h/day). These categories demonstrate commonly used thresholds in the literature (≤3 h/day for low sitting, >3–8 h/day for moderate sitting, >8–14 h/day for high sitting) while adding an original category capturing extreme sitting behavior (>14 h/day), made possible by the size and diversity of our dataset.

Therefore, we aimed to investigate the influence of telework on sedentary behavior, taking into account major sociodemographic, occupational and lifestyle factors, using statistical models that simultaneously combining all putative variables that may influence sedentary behavior.

## Methods

### Study design

The COVISTRESS study is an international, cross-sectional online survey assessing various sociodemographic, occupational, and lifestyle behavior using a self-reported questionnaire. Data from this study were collected from 1^st^ January 2023 until 10^th^ March 2025. Participants answered the questionnaire through the COVISTRESS.org website. It was distributed by any distribution method, i.e., through informatic (social media, mailing list of organizations, etc.) and printed format (flyers distributed in places open to the public, such as supermarkets or shops). The secure internet application REDCap^®^ was used to build and manage the questionnaire, hosted by the University Hospital of Clermont-Ferrand (CHU Clermont-Ferrand). It has received an approval from the Ethical Committee South-East VI, France and has been registered at ClinicalTrials.gov (Clinical trial number NCT04538586). Participation was entirely voluntary and anonymous. Participants were self‑selecting. Prior to accessing the questionnaire, participants were provided with detailed written information regarding the study’s purpose, procedures, data confidentiality, and the voluntary nature of participation. Proceeding to the questionnaire constituted informed electronic consent. Written consent was not required, and this procedure was approved by the Ethical Committee South-East VI, France (CPP Sud-Est VI), which waived the need for written consent given the anonymous, minimal-risk design. Only participants (≥16 years) were eligible; no minors were enrolled. No personally identifiable information is collected in the COVISTRESS survey, ensuring full compliance with ethical and data protection requirements. This study complies with the guidelines of the Declaration of Helsinki.

### Participants

**Fig 1:**
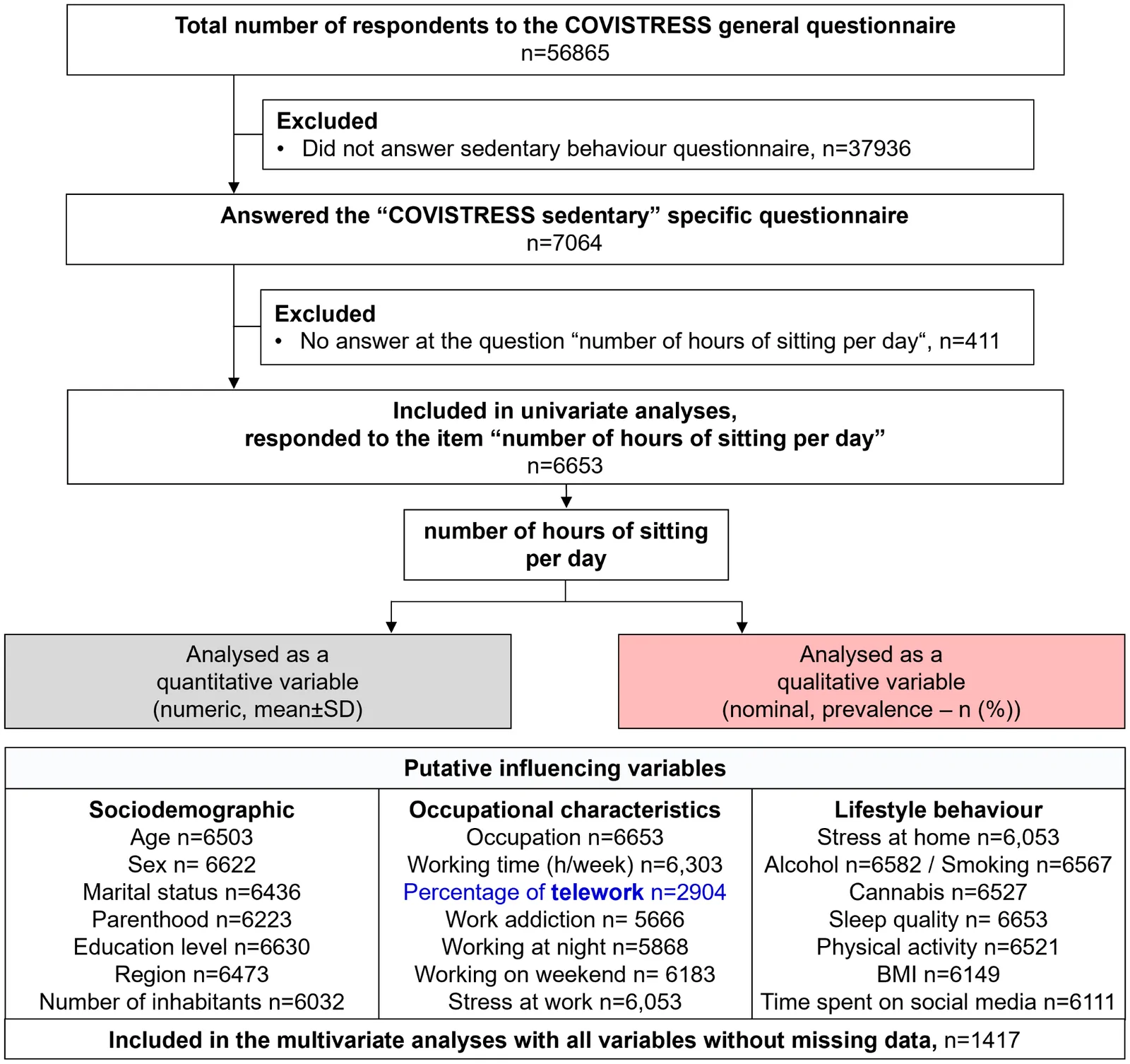
Flow chart.

The COVISTRESS study was designed for general public without exclusion criteria. We included all participants who answered their sitting time (Fig 1). However, analyses focused on employed participants, as teleworking is only applicable for workers.

### Survey variables

**Outcome and exposure** variables were sedentary behavior and telework. Sedentary behavior was measured by answering the question ‘Number of hours sitting per day’ with a drop-down menu between 0 and 24, with 30 min intervals. Sitting time was used as a quantitative data and as a qualitative data (≤3, >3–8, >8–14, and >14 hours sitting per day). We treated the outcome using both continuous and categorical analyses to strengthen the robustness of our findings. Continuous models test overall trends, while categorical models identify threshold effects and facilitate interpretation. The exposure (telework) was measured by answering the question ‘Percentage of telework’. Telework was originally presented in the questionnaire in ordered categories (0%, 1–25%, 26–50%, 51–75%, 76–99%, 100%).

**Covariates** (other influencing variables) were sociodemographic, occupational, and lifestyle variables. All secondary variables were categorized based on the structure of the original questionnaire, established epidemiological standards (for Body mass index – BMI – and stress level), and percentile-based cut-offs. Sociodemographic were age (≤42 vs >42 years old); sex (men vs women); marital status (in a relationship vs single); parenthood (no child vs ≥1 child); education level (≤high school, undergraduate, master degree, doctorate); number of inhabitants (≤5000, >5000–1 million, >1 million). Occupational variables were occupation (superior, intermediary, employee, others – in detail 1) superior occupations refers to executives and senior intellectual professionals, e.g., highly skilled professionals, engineers, senior administrators or managers; 2) intermediary professions, e.g., technicians, mid-level supervisors, administrative coordinators; 3) workers or employees, e.g., manual, clerical, or service workers; and 4) others, e.g., farmers, artisans, merchants or entrepreneurs, information and arts professions, self-employed individuals) –; declared working time per week (≤30h, >30-40h, >40-50h, >50h); working at night or working on weekend; level of stress at work measured using an analog visual scale (low level of stress <50/100, intermediate 50–80, high >80) [54]. Lifestyle behavior variables were stress at home (<50, 50–80, >80); alcohol (yes vs no) and tobacco (yes vs no) consumption; physical activity (≤30 min, >30min-1h, >1h-2h, >2h-4h, and >4h/week); body mass index (<18.5 kg/m^2^ underweight, 18.5-24.99 kg/m^2^ normal, ≥25 kg/m^2^ overweight, ≥30 kg/m^2^ obesity) [55]; and time spent on social media (≤20 min, >20min-1h, >1h-3h, >3h/day).

### Statistical analysis

**Fig 2:**
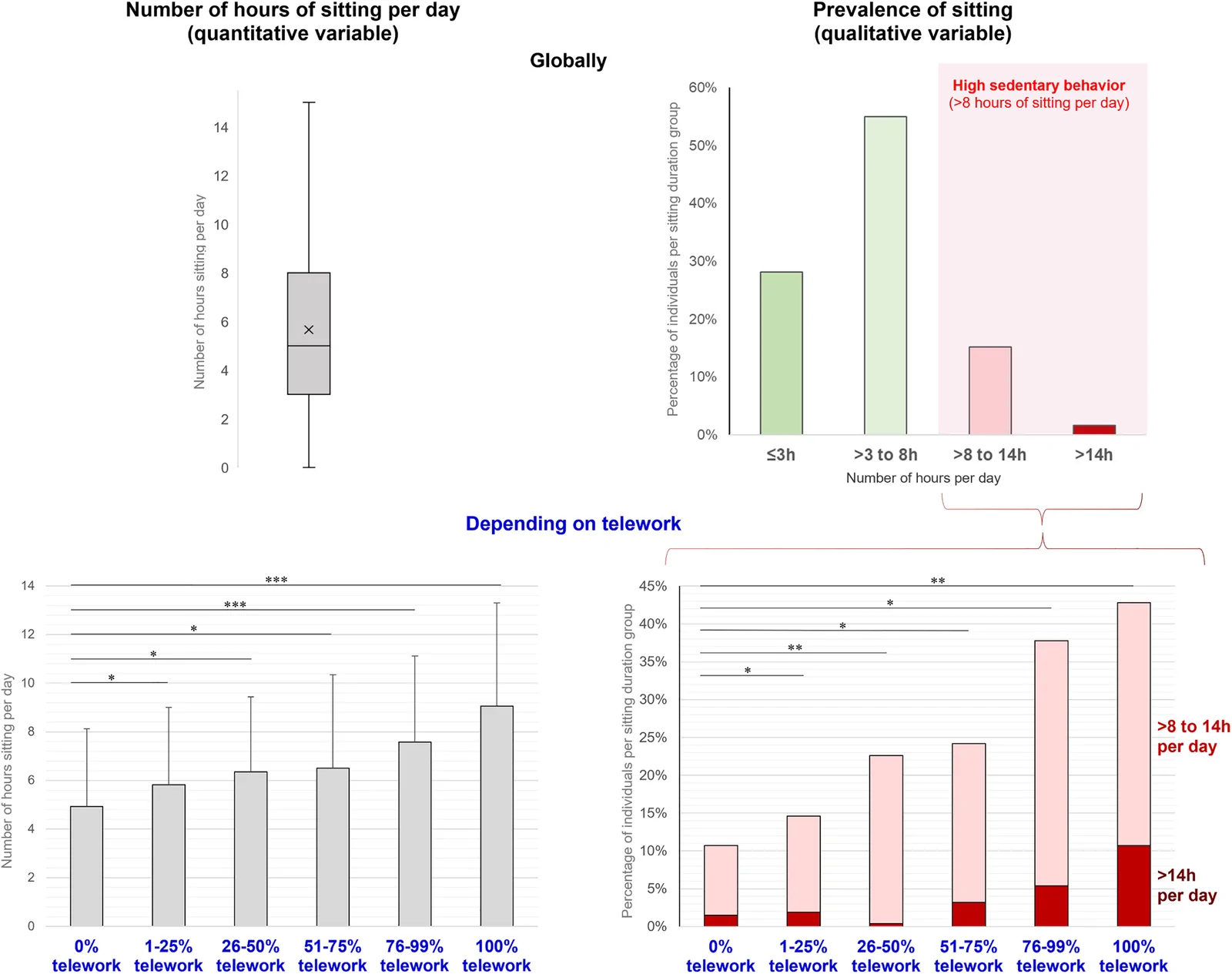
Sedentary behavior as quantitative and qualitative variable globally and depending on percentage of telework. In the box and whisker plot (sitting time in grey), the lower and upper sides of the box are the lower and upper quartiles (Q1 and Q3). The box covers the interquartile interval (IQR), where 50% of the data is found. The horizontal line that splits the box in two is the median. The mean is indicated by a cross in the box plot. The whiskers are the two vertical lines outside the box, that go from −1.5 IQR to Q1 and then from Q3 to +1.5 IQR. Depending on telework, grey histograms are means and vertical line is the standard deviation (SD), and stars are significant Cohen’s d effect-size: * ≥0.20, ** ≥0.50, *** ≥0.80; and red histograms are prevalence of sitting with stars being significant Cramer’s V effect size: * ≥0.10, ** ≥0.15, *** ≥0.25.

**Fig 3:**
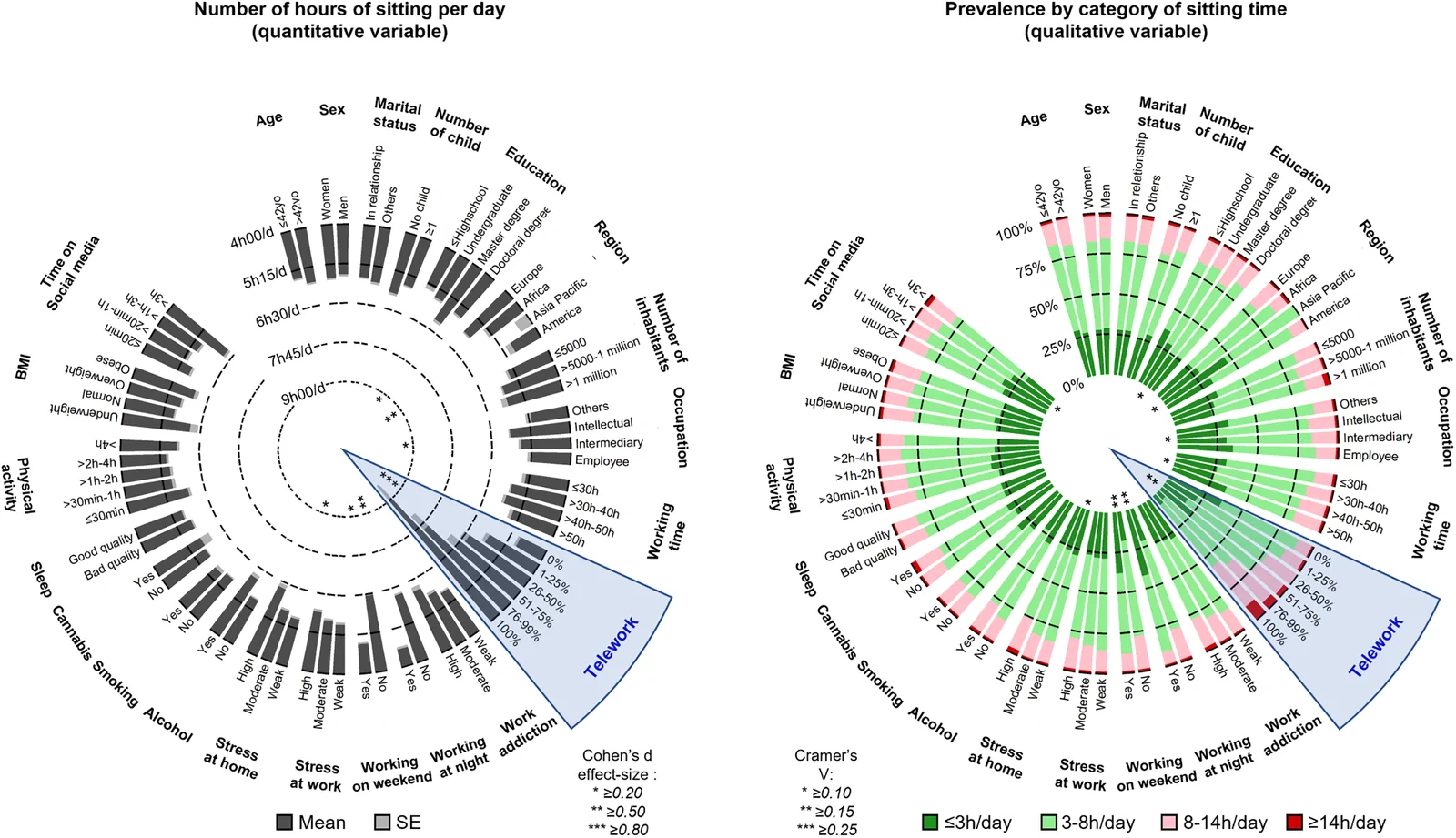
Sitting time depending on sociodemographic, occupational, and lifestyle factors (polar plots). Sitting time as a quantitative variable (mean±SD) were compared between groups using a Student t-test for 2-group comparisons (or a Wilcoxon-Mann-Whitney test if data were not normally distributed), and using an analysis of variance (ANOVA) for 3 or more groups comparisons (or a Kruskal-Wallis test if data were not normally distributed). Results were interpreted by using Cohen’s d effect-size (ES) and 95% confident intervals (95 CI). ES ≥0.20 were considered small (*), ≥0.50 moderate (**), and ≥0.80 large (***). The prevalence of sitting durations (qualitative variable, i.e., number of people spending ≤3h, >3h to ≤8h, >8h to ≤14h, and >14h sitting per day) was compared between groups using a Chi^2^ test. To quantify the strength of the association between secondary outcomes and sitting durations, Cramer’s V was calculated. Cramer’s V ≥0.10 were considered small (*), ≥0.15 moderate (**), and ≥0.25 strong (***). Stars show the highest significance between two groups.

**Fig 4:**
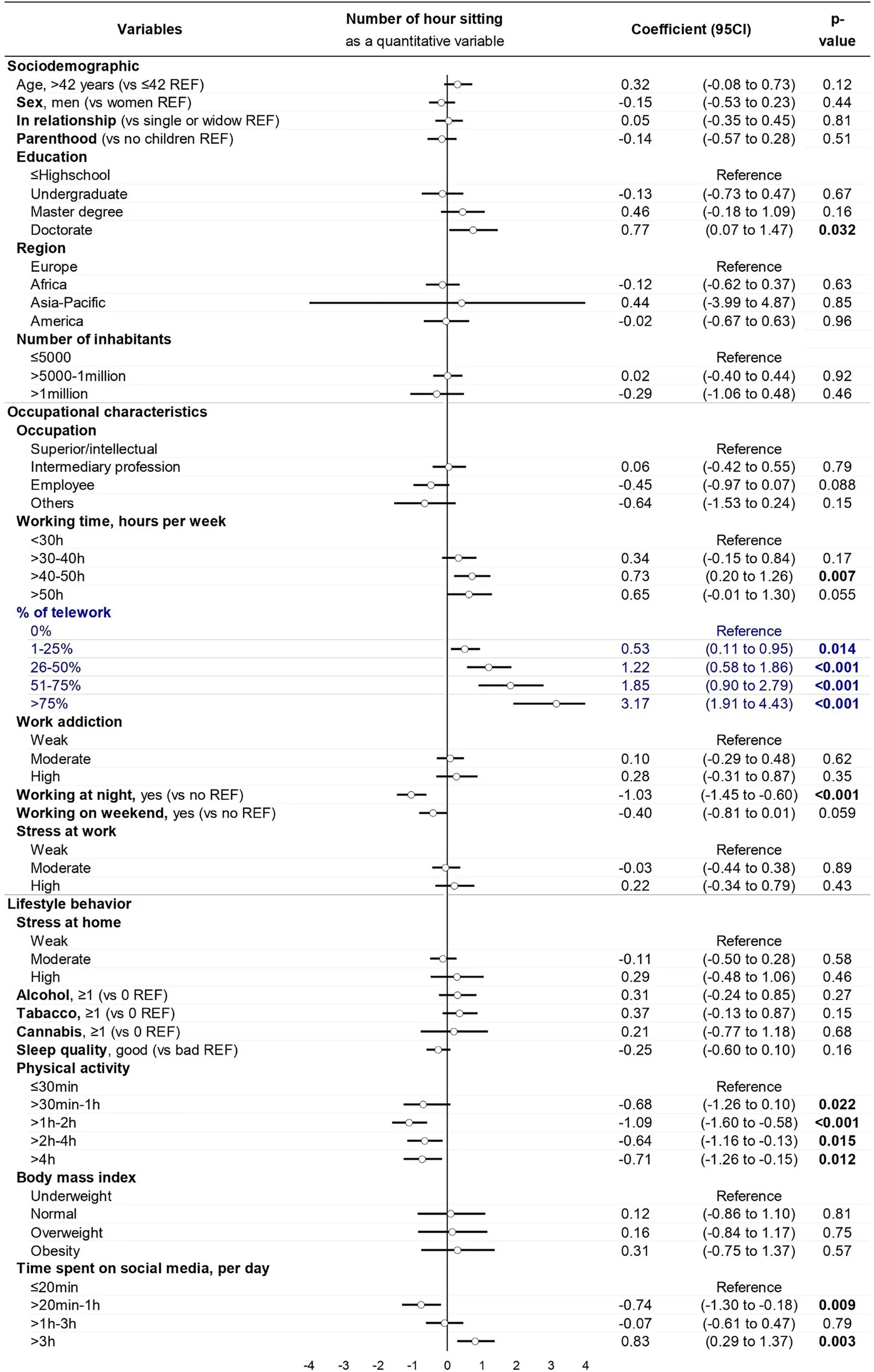
Factors influencing number of hours of sitting time using multivariate linear regression. The effect of each variable on the number of hours of sitting time is represented by a dot on a horizontal line in the forest-plot. The dots represent the effect (coefficient) for each variable, and the length of each line around the dots represent their 95% confidence interval (95 CI). The black solid vertical line represents the null estimate (with a value of 0). Coefficients with horizontal lines that do not cross the null vertical line are significant. Significant variables with a coefficient <0 are factors decreasing sitting time and those with a coefficient >0 are factors increasing sitting time. REF: Reference, i.e., the reference for group comparisons.

**Fig 5:**
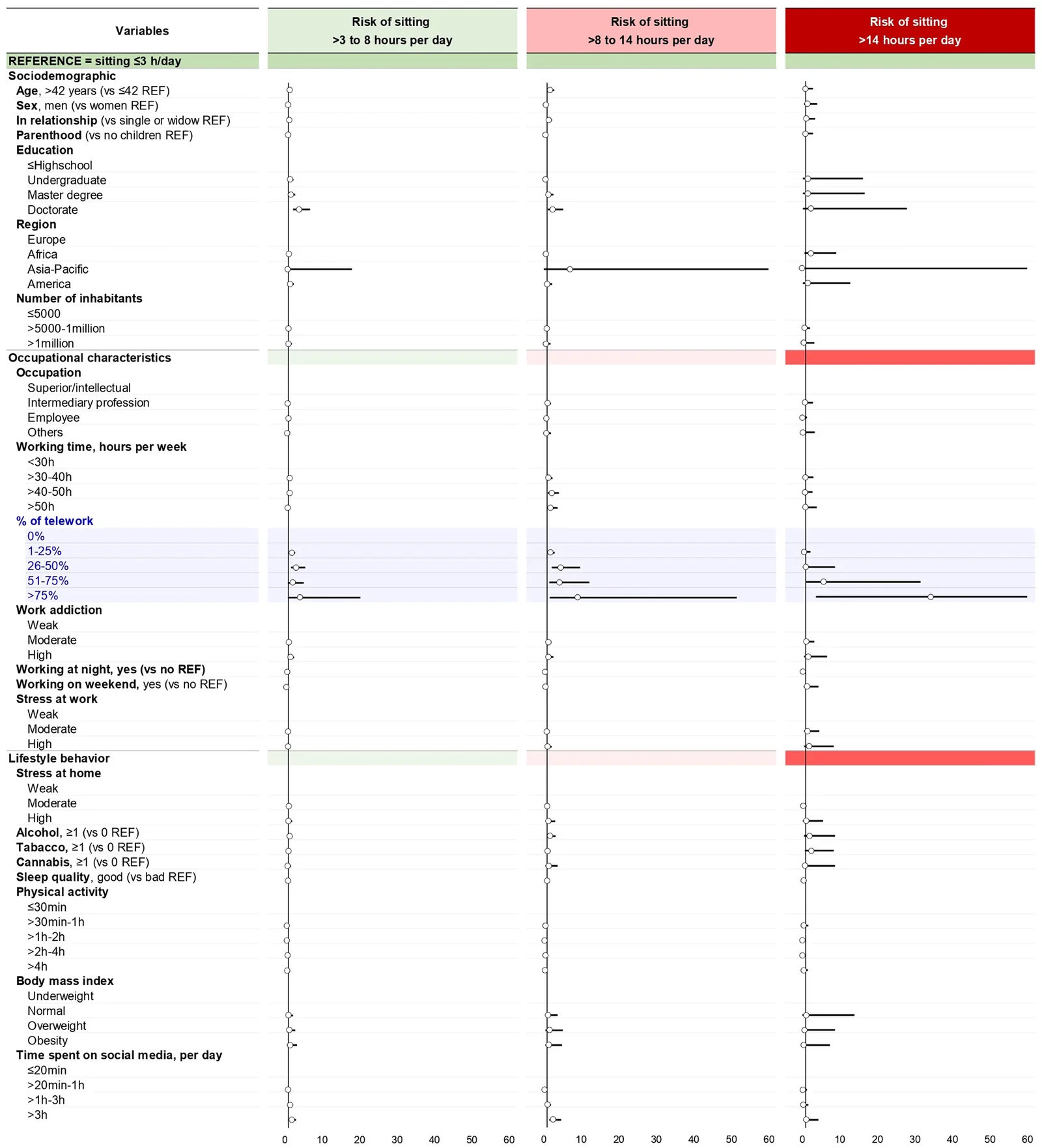
Summary of factors influencing the risk of sitting using multivariate logistic regression. The effect of each variable on the risk of sitting >3–8 hours, >8–14 hours, and >14 hours per day is represented by a dot on a horizontal line in the forest-plot. The dots represent the risk of high sitting time (odds ratio) for each variable, and the length of each line around the dots represent their 95% confidence interval (95 CI). The black solid vertical line represents the null estimate (with a value of 1). Odds ratio with horizontal lines that do not cross the vertical line are significant. Significant variables with an odds ratio <1 are protective factors decreasing the risk if high sitting time and those with an odds ratio >1 are risk factors. REF: Reference, i.e., the reference for group comparisons.

Statistical analyses were conducted using Stata® software (v15, StataCorp, College Station, USA). Quantitative variables were described as mean and standard deviation, and qualitative, i.e., categorical variables were presented as number and associated percentage (%). Number of hours of sitting (quantitative variable) was compared between groups of telework (0%, 1–25%, 26–50%, 51–75%, >75%) using a Student t-test for 2-group comparisons (or a Wilcoxon-Mann-Whitney test if data were not normally distributed), and using an analysis of variance (ANOVA) for 3 or more groups comparisons (or a Kruskal-Wallis test if data were not normally distributed). Results were interpreted by using Cohen’s d effect-size (ES) and 95% confident intervals (95 CI). ES ≥ 0.10 were considered weak (tendency ^†^), ≥0.20 were considered small (*), ≥0.50 moderate (**), and ≥0.80 large (***). The prevalence of sitting durations (qualitative variable, i.e., number of people sitting ≤3h, >3h-8h, >8h-14h, and >14h per day) was compared between groups of telework using a Chi^2^ test. To quantify the strength of the association between secondary outcomes and sitting durations, Cramer’s V was calculated. Cramer’s V measures effect size from 0 to 1, where 0 indicates no association, values >0.05 indicate a weak effect (^†^), >0.10 a moderate effect (*), >0.15 a strong effect (**), and >0.25 a very strong (***) relationship (Table 1 and Fig 2) [56]. Those analysis were repeated to compare sitting time between groups for other variables (sociodemographic, occupational, and lifestyle variables) (Table 1 and Fig 3). Furthermore, univariate (S1 Fig and S3 Fig) then multivariate (Fig 4 and 5, and S2 Fig) regression models were used to assess the relationship between sitting time and telework, taking into account sociodemographic, occupational, and lifestyle factors. The results were presented as coefficient, 95 CI, and corresponding p-values for linear regression, i.e., using sitting time as a continuous data (Fig 4 and S1 Fig). The results were presented as odds ratio (OR), 95 CI and corresponding p-values for logistic regression, i.e., using sitting time as a qualitative data (the risk of sitting >3h-8h, >8h-14h, and >14h compared with sitting ≤3h per day as reference) (Fig 5 and S2 Fig and S3 Fig). We conducted sensitivity analyses to check the accuracy of our regression estimates, by verifying multicollinearity and interactions between covariates. First, we calculated the variance inflation factor (VIF) (S1 Table). A VIF ≤1 indicates no correlation between the covariates, <5 a moderate correlation generally acceptable, <10 a potentially problematic multicollinearity, and ≥10: a serious multicollinearity that may require further investigation. We further performed several prespecified sensitivity analyses to strengthen the robustness of our findings. We studied the relationship between covariables to evaluate the impact by removing the less answered variables on multivariate regression analysis, i.e., marital status, parenthood, number of inhabitants, and work addiction (S4 Fig). We conducted step by step regression analyses by adding variables one by one in addition to telework to verify the consistency of the association between sitting time and telework (S2 Table). A complete-case approach with multivariate model using missing data as a specific class for each variable was also performed (S5 Fig). Furthermore, in order to search for the pattern of missing data, we conducted assessment of non‑response bias through comparison of complete and incomplete respondents (S3 Table). Statistical significance was marked by p-value ≤0.05. There was no imputation of missing data.

**Table 1:** Sitting time depending on sociodemographic, occupational, and lifestyle factors. Sitting time as a quantitative variable (mean±SD) were compared between groups using a Student t-test for 2-group comparisons (or a Wilcoxon-Mann-Whitney test if data were not normally distributed), and using an analysis of variance (ANOVA) for 3 or more groups comparisons (or a Kruskal-Wallis test if data were not normally distributed). Results were interpreted by using Cohen’s d effect-size (ES) and 95% confident intervals (95 CI). ES ≥ 0.10 were considered a tendency (†), ES ≥0.20 were considered small (*), ≥0.50 moderate (**), and ≥0.80 large (***). The prevalence of sitting durations (qualitative variable, i.e., number of people spending ≤3h, >3h to ≤8h, >8h to ≤14h, and >14h sitting per day) was compared between groups using a Chi² test. To quantify the strength of the association between secondary outcomes and sitting durations, Cramer’s V was calculated. Cramer’s V ≥0.05 were considered a tendency (†), ≥0.10 were considered small (*), ≥0.15 moderate (**), and ≥0.25 strong (***).

	n	Sedentary behavior									
		Quantitative variable				Qualitative variable					
		(Number of hours sitting/day)				(Prevalence i.e. number of individuals per sitting duration group)					
		Mean ± SD	p-value	ES	(95CI)	≤3h	3h-8h	8h-14h	>14	p-value	ES
Sociodemographic						n (%)					
Age											
≤42yo	3561	5.52 ± 3.38	<0.001	REF		1,055 (29.6)	1,957 (55.0)	483 (13.6)	66 (1.8)	0.001	REF
>42yo	2942	5.74 ± 3.30		0.07	(0.02 to 0.11)	799 (27.2)	1,627 (55.3)	480 (16.3)	36 (1.2)		0.05†
Sex											
Women	4291	5.70 ± 3.31	0.02	REF		1,191 (27.7)	2,354 (54.9)	687 (16.0)	59 (1.4)	0.019	REF
Men	2331	5.59 ± 3.45		–0.03	(–0.08 to 0.02)	673 (28.9)	1,285 (55.1)	324 (13.9)	49 (2.1)		0.04
Marital status											
In relationship	4548	5.76 ± 3.50	0.78	REF		1,269 (27.9)	2,508 (55.2)	706 (15.5)	65 (1.4)	0.14	REF
Others	1888	5.66 ± 3.32		–0.03	(–0.08 to 0.03)	532 (28.2)	1,020 (54.0)	294 (15.6)	42 (2.2)		0.03
Parenthood											
No child	1940	5.96 ± 3.49	<0.001	REF		488 (25.2)	1,057 (54.4)	355 (18.3)	40 (2.1)	<0.001	REF
≥ 1 child	4283	5.60 ± 3.31		–0.11†	(–0.16 to –0.05)	1,243 (29.0)	2,360 (55.1)	615 (14.4)	65 (1.5)		0.06^†^
Education level											
≤High school	859	5.31 ± 3.71	<0.001	REF		314 (36.6)	400 (46.6)	127 (14.8)	18 (2.1)	<0.001	REF
Undergraduate	2121	5.54 ± 3.31		0.07	(–0.01 to 0.14)	631 (29.8)	1,172 (55.3)	290 (13.7)	28 (1.3)		0.08^†^
Master degree	1596	6.14 ± 3.44		0.23*	(0.15 to 0.32)	378 (23.7)	873 (54.7)	313 (19.6)	32 (2.0)		0.14*
Doctoral degree	2054	5.59 ± 3.16		0.18^†^	(0.12 to 0.25)	543 (26.4)	1,198 (58.3)	283 (13.8)	30 (1.5)		0.11*
Region											
Europe	4667	5.85 ± 3.38	<0.001	REF		1,280 (27.5)	2,488 (53.3)	828 (17.7)	71 (1.5)	<0.001	REF
Africa	1507	5.29 ± 3.29		–0.17†	(–0.23 to –0.11)	436 (28.9)	902 (59.9)	139 (9.2)	30 (2.0)		0.10*
Asia Pacific	15	4.03 ± 1.64		–0.54**	(–1.05 to –0.03)	7 (46.7)	8 (53.3)	0 (0.0)	0 (0.0)		0.03
America	284	4.94 ± 3.10		–0.27*	(–0.39 to –0.15)	92 (32.4)	162 (57.0)	27 (9.5)	3 (1.1)		0.05^†^
Number of inhabitants											
≤ 5000	1694	5.44 ± 3.21	0.003	REF		531 (31.3)	901 (53.2)	243 (14.3)	19 (1.1)	<0.001	REF
>5000–1 million	3957	5.80 ± 3.41		0.10^†^	(0.05 to 0.16)	1068 (27.0)	2169 (54.8)	656 (16.6)	64 (1.6)		0.05^†^
>1 million	381	5.80 ± 3.56		0.11^†^	(0.002 to 0.22)	92 (24.1)	229 (60.1)	46 (12.1)	14 (3.7)		0.10†
Occupational characteristics											
Occupation											
Superior/intellectual	3547	5.90 ± 3.29	<0.001	REF		861 (24.3)	2,039 (57.5)	587 (16.5)	60 (1.7)	<0.001	REF
Intermediary profession	1447	5.57 ± 3.38		–0.10^†^	(–0.16 to –0.04)	443 (30.6)	763 (52.7)	221 (15.3)	20 (1.4)		0.07^†^
Employee	1338	5.28 ± 3.48		–0.19^†^	(–0.25 to –0.12)	463 (34.6)	685 (51.2)	168 (12.6)	22 (1.6)		0.11*
Others	321	5.17 ± 3.28		–0.22*	(–0.33 to –0.11)	105 (32.7)	172 (53.6)	38 (11.8)	6 (1.9)		0.06^†^
Working time (per week)											
≤ 30h	991	5.37 ± 3.39	<0.001	REF		310 (31.3)	548 (55.3)	112 (11.3)	21 (2.1)	<0.001	REF
>30–40h	2762	5.75 ± 3.19		0.11^†^	(0.04 to 0.19)	730 (26.4)	1,572 (56.9)	427 (15.5)	33 (1.2)		0.07^†^
>40–50h	1569	5.84 ± 3.49		0.13^†^	(0.05 to 0.21)	403 (25.7)	875 (55.8)	257 (16.4)	34 (2.1)		0.08^†^
>50h	981	5.48 ± 3.60		0.03	(0.06 to 0.12)	348 (35.5)	447 (45.6)	169 (17.2)	17 (1.7)		0.11*
Percentage of telework											
0%	1792	4.93 ± 3.20	<0.001	REF		648 (36.2)	953 (53.2)	164 (9.1)	27 (1.5)	<0.001	REF
1–25%	670	5.83 ± 3.18		0.28*	(0.19 to 0.37)	146 (21.8)	426 (63.6)	85 (12.7)	13 (1.9)		0.14*
26–50%	225	6.36 ± 3.08		0.45*	(0.31 to 0.59)	39 (17.3)	135 (60.0)	50 (22.2)	1 (0.4)		0.17**
51–75%	124	6.51 ± 3.84		0.49*	(0.31 to 0.67)	31 (25.0)	63 (50.8)	26 (21.0)	4 (3.2)		0.11*
76–99%	37	7.58 ± 3.53		0.83***	(0.50 to 1.15)	2 (5.4)	21 (56.8)	12 (32.4)	2 (5.4)		0.14*
100%	56	9.05 ± 4.25		1.27***	(1.01 to 1.54)	4 (7.1)	28 (50.0)	18 (32.2)	6 (10.7)		0.19**
Work addiction											
Weak	3641	5.66 ± 3.32	0.007	REF		1,026 (28.2)	2,008 (55.2)	554 (15.2)	53 (1.4)	0.007	REF
Moderate	1282	5.81 ± 3.39		0.05	(–0.02 to 0.12)	342 (26.7)	700 (54.6)	219 (17.1)	21 (1.6)		0.03
High	743	6.14 ± 3.65		0.14^†^	(0.06 to 0.22)	193 (26.0)	384 (51.7)	145 (19.5)	21 (2.8)		0.06^†^
Working at night											
No	3842	6.24 ± 3.32	<0.001	REF		814 (21.2)	2,209 (57.5)	753 (19.6)	66 (1.7)	<0.001	REF
Yes	2026	4.58 ± 3.17		–0.51**	(–0.56 to –0.45)	849 (41.9)	987 (48.7)	163 (8.1)	27 (1.3)		0.24**
Working on weekend											
No	2929	6.45 ± 3.24	<0.001	REF		532 (18.2)	1,745 (59.6)	604 (20.6)	48 (1.6)	<0.001	REF
Yes	3254	4.94 ± 3.33		–0.46*	(–0.51 to –0.41)	1,233 (37.9)	1,622 (49.8)	345 (10.6)	54 (1.7)		0.23**
Stress at work											
Weak	1859	5.57 ± 3.24	0.024	REF		530 (28.5)	1,055 (56.7)	247 (13.3)	27 (1.5)	<0.001	REF
Moderate	2587	5.68 ± 3.34		0.04	(–0.02 to 0.09)	728 (28.1)	1,409 (54.5)	407 (15.7)	43 (1.7)		0.04
High	1607	5.91 ± 3.51		0.10^†^	(0.03 to 0.17)	433 (26.9)	839 (52.2)	305 (19.0)	30 (1.9)		0.08^†^
Lifestyle behavior											
Stress at home											
Weak	2830	5.54 ± 3.29	<0.001	REF		826 (29.2)	1,567 (55.4)	395 (14.0)	42 (1.4)	<0.001	REF
Moderate	2292	5.68 ± 3.24		0.04	(–0.01 to 0.10)	627 (27.4)	1,281 (55.9)	352 (15.3)	32 (1.4)		0.03
High	931	6.31 ± 3.86		0.22*	(0.15 to 0.30)	235 (25.2)	471 (50.6)	195 (21.0)	30 (3.2)		0.10*
Alcohol											
0	5700	5.59 ± 3.35	<0.001	REF		1,662 (29.1)	3,117 (54.7)	832 (14.6)	89 (1.6)	<0.001	REF
≥ 1	882	6.16 ± 3.40		0.17^†^	(0.10 to 0.24)	196 (22.2)	493 (55.9)	175 (19.8)	18 (2.04)		0.07^†^
Smoking											
0	5571	5.68 ± 3.33	0.082	REF		1,528 (27.4)	3,116 (55.9)	838 (15.04)	89 (1.6)	<0.001	REF
≥ 1	996	5.57 ± 3.53		–0.03	(–0.10 to 0.03)	327 (32.8)	486 (48.8)	165 (16.57)	18 (1.8)		0.05†
Cannabis											
0	6352	5.67 ± 3.34	0.51	REF		1,785 (28.1)	3,493 (55.0)	974 (15.3)	100 (1.6)	0.15	REF
≥ 1	175	5.74 ± 4.05		0.02	(–0.13 to 0.17)	54 (30.9)	86 (49.1)	29 (16.6)	6 (3.4)		0.03
Sleep quality											
Bad quality	3536	5.74 ± 3.45	0.12	REF		997 (28.2)	1,909 (54.0)	566 (16.0)	64 (1.8)	0.12	REF
Good quality	3117	5.58 ± 3.26		–0.05	(–0.10 to –0.001)	875 (28.1)	1,750 (56.1)	448 (14.4)	44 (1.4)		0.03
Physical activity (per week)											
≤30min	1665	5.97 ± 3.61	<0.001	REF		449 (27.0)	882 (53.0)	294 (17.6)	40 (2.4)	<0.001	REF
>30min–1h	1002	5.53 ± 3.35		–0.13^†^	(–0.20 to –0.05)	309 (30.8)	528 (52.7)	153 (15.3)	12 (1.2)		0.06^†^
>1h–2h	1330	5.53 ± 3.34		–0.13^†^	(–0.20 to –0.06)	400 (30.1)	708 (53.2)	205 (15.4)	17 (1.3)		0.06^†^
>2h–4h	1373	5.44 ± 3.03		–0.16^†^	(–0.23 to –0.09)	379 (27.6)	798 (58.1)	183 (13.3)	13 (1.0)		0.08^†^
>4h	1151	5.78 ± 3.32		–0.06	(–0.13 to 0.02)	295 (25.6)	668 (58.0)	166 (14.4)	22 (1.9)		0.06^†^
Body mass index											
Underweight	233	6.16 ± 3.92	<0.001	REF		63 (27.0)	121 (51.9)	44 (18.9)	5 (2.2)	0.15	REF
Normal	3421	5.58 ± 3.33		–0.17^†^	(–0.31 to –0.04)	990 (28.9)	1,879 (54.9)	494 (14.4)	58 (1.7)		0.03
Overweight	1697	5.71 ± 3.23		–0.13^†^	(–0.27 to 0.002)	447 (26.3)	952 (56.1)	275 (16.2)	23 (1.4)		0.04
Obesity	798	6.04 ± 3.58		–0.03	(–0.18 to 0.11)	207 (25.9)	437 (54.8)	137 (17.2)	17 (2.1)		0.03
Time spent on social media											
≤ 20min	1526	5.63 ± 3.29	<0.001	REF		451 (29.6)	807 (52.9)	246 (16.1)	22 (1.4)	<0.001	REF
>20min–1h	1353	5.32 ± 3.19		–0.10^†^	(–0.17 to –0.02)	441 (32.6)	725 (53.5)	174 (12.9)	13 (1.0)		0.06^†^
>1h–3h	1471	5.49 ± 3.20		–0.06	(–0.12 to 0.03)	433 (29.4)	798 (54.3)	225 (15.3)	15 (1.0)		0.02
>3h	1761	6.27 ± 3.64		0.18^†^	(0.12 to 0.25)	380 (21.6)	1,014 (57.5)	315 (18.0)	52 (2.9)		0.10*

## Results

### Characteristics of participants

Among the 7064 respondents who answered the COVISTRESS sedentary questionnaire in 2023 and 2024, 6653 answered the sitting time item and were included. Among them, 2904 responded both to sitting time and telework items, and 1417 responded to all questions without any missing data (Fig 1). Respondents were aged 41.8 ± 11.4 years, 64.8% were women. About two thirds were in relationships (70.7%), with at least one child (68.8%). 13% had an education level less or equal to high school, 32% were undergraduate, 24.1% master degree, and 31% doctoral degree. Most of the respondents were from Europe (72.1%), followed by Africa (23.3%) and a small percentage of respondents from America (4.4%) and Asia Pacific (0.2%). For occupational characteristics, 53.3% of participants was executives and senior intellectual professionals, 21.8% was intermediary professions, and 20.1% was employees. Among participants, 13.1% had a high work addiction, and 26.6% had high level of stress at work. Furthermore, for lifestyle variables, 15.4% had high level of stress at home, 13.4% frequently drank alcohol, 15.1% were smokers, 2.6% used cannabis, 27.6% were overweight and 13.0% obese, and 52.9% used social media >1h/day.

### Sedentary behavior and telework

The mean sitting time was 5.7 ± 3.4h/day, with 15.2% of respondents sitting for 8-14h/day and 1.6% sitting >14 hours/day. 61.7% of respondents did telework and 7.5% worked more than 50% in telework. It’s important to note that among the respondents who did telework, 85.9% spent <8h/day sitting. There was a dose response relation between telework and sitting time: workers who teleworked 0%, 1–25%, 26–50%, 51–75%, 76–99%, and 100% respectively sat on average 4.9 ± 3.2h/day, 5.8 ± 3.2h/day, 6.4 ± 3.1h/day, 6.5 ± 3.8h/day, 7.6 ± 3.5h/day, and 9.1 ± 4.3h/day, with a gradual increase in the effect sizes (from a Cohen’s d of 0.28 for 1–25% telework to 1.27 for 100% telework, in comparison with 0% telework). Similarly, for workers who teleworked 0%, 1–25%, 26–50%, 51–75%, 76–99%, and 100%, prevalence of workers sitting 8 to 14h/d increased from 9.1%, 12.7%, 22.2%, 21%, 32.4% to 32.2% respectively, as well as for prevalence of workers sitting more than 14h/d that increased from 1.5%, 1.9%, 0.4%, 3.2%, 5.4% to 10.7%, with also a gradual increase in the effect size (from a Cramer’s V of 0.14 for 1–25% telework to 0.19 for 100% telework, in comparison with 0% telework) (Table 1 and Fig 2).

### Mean sitting time depending on sociodemographic, occupational and lifestyle factors

Using number of hours of sitting as a quantitative variable (mean sitting time), only some variables were associated to sitting time with a Cohen ES ≥0.20. Regarding sociodemographic, in comparison with people having a level equal or under high school (5.3 ± 3.7 hours/day sitting), sitting time was higher for respondents with a master degree (6.1 ± 3.4 h/d; ES = 0.23, 95 CI 0.15 to 0.32) or doctorate (5.59 ± 3.16 h/d; ES = 0.18, 0.12 to 0.25). Compared to people living in Europe (5.9 ± 3.4 h sitting/day), those living in Asia Pacific had a lower sitting time (4.0 ± 1.6 h sitting/day; ES = −0.54, −1.05 to −0.03), as well as those living in America (4.9 ± 3.1, ES = −0.27, −0.39 to −0.15). Regarding occupational variables, superior/intellectual workers had the highest sitting time (5.9 ± 3.3 h/day) compared with intermediary professions (5.6 ± 3.4 h sitting/day; ES = −0.10, −0.16 to −0.04), employees (5.3 ± 3.5 h/d; ES = −0.19, −0.25 to −0.12), and others (5.2 ± 3.3 h/d; ES = −0.22, −0.33 to −0.11). Those who worked at night had lower sitting time (4.6 ± 3.2 vs 6.2 ± 3.3 h sitting/day for day-time workers; ES = −0.51, −0.56 to −0.45), as well as those working on weekends (4.9 ± 3.3 vs 6.5 ± 3.2 h sitting/day for those who did not work on weekends; ES = −0.46, −0.51 to −0.41). Regarding lifestyle variables, people with high stress at home had higher sitting time than those with a low level (6.3 ± 3.9 vs 5.5 ± 3.3 h sitting/day, ES = 0.22, 0.15 to 0.30). There were weak associations (ES ≥0.10) for higher sitting time for people without having children, living in cities with 5000−1 million and ≥1 million inhabitants, working >40-50h and 30-40h vs <30 h/week, having a high level of work addiction and stress at work, alcohol, lower physical activity, BMI, and time spent on social media. Despite nearly all variables being associated with sitting time (at least weakly), only telework had very large and strong ES (Cohen’s D > 0.80) (Table 1 and Fig 3).

### Prevalence of high sitting time depending on sociodemographic, occupational and lifestyle factors

As a qualitative variable (prevalence of sitting time), only some variables were associated to a high prevalence of sitting time with a Cramer’s V ES ≥ 0.10. The prevalence of sitting >8 h/day were higher in people with master degree vs those ≤ high school (21.6 vs 16.9%, ES = 0.14), in people living in Europe vs Africa (19.2 vs 11.2%, ES = 0.10), living in the megalopolis with >1 million inhabitants (15.8 vs 15.4%, ES = 0.10), in superior and intellectual professions compared to employees (18.2 vs 14.2%, ES = 0.11), those working more than 50h/week compared to those working ≤30 h/week (18.9 vs 13.4%, ES = 0.11), not working at night (21.3 vs 9.4%, ES = 0.24) and not working on weekend (22.2 vs 12.3%, ES = 0.23), those with a high level of stress at home (24.2 vs 15.4% for those with a low level of stress at home, ES = 0.10), and those spending > 3h on social media (20.9 vs 17.5%, ES = 0.10). There were weak associations (ES ≥ 0.05) for a higher prevalence of high sitting time for older people, without children, having a high level of work addiction and stress at work, alcohol, smoking, and lower physical activity (Table 1 and Fig 3).

### Factors influencing sedentary behavior (multivariate linear regression)

Globally, all variables that influenced sitting time in univariate linear regression (S1 Fig) were also significant in multivariate linear regression. In particular, multivariate regression confirmed the influence of telework on sitting time. Compared with the group without telework, sitting time increased by 0.53 hours per day (95 CI 0.11 to 0.95 h/d) for 1−25% telework, by 1.22 hours per day (−0.58 to 1.86 h/d) for 26−50% telework, by 1.85 hours sitting/day (0.90 to 2.79 h sitting/d) for 51−75% telework, by 3.17 h sitting/day (1.91 to 4.43 h sitting/d) for >75% telework. People who have an education doctoral degree increased sitting time by 0.77 h sitting/day (0.07 to 1.47 h sitting/d), working >40-50h/week increased sitting time by 0.73 h sitting/day (0.20 to 1.26 h sitting/d), while number of hours of sitting decreased in those working at night (−1.03 h sitting/day, −1.45 to −0.60 h sitting/day), with physical activity (−0.68, −1.26 to 0.10 h sitting/day for 1h of physical activity/week; −1.09, −1.60 to −0.58 h sitting/day for 1−2 hours of physical activity/week; −0.64, −1.16 to −0.13 h sitting/day for 2-4h of physical activity/week; and −0.71, −1.26 to −0.15 h sitting/day for >4 hours of physical activity/week), and spending >3h/day on social media increased sitting time by 0.83 h sitting/day (0.29 to 1.37 h sitting/d) (Fig 4).

### Risk factor of sedentary (multivariate logistic regression)

Similar to linear regression, all variables that influenced the risk of prolonged sitting in univariate logistic regression (S3 Fig) were also significant in multivariate logistic regression. There was a major dose response relation between sitting time and telework. Compared with workers who did not telework and who sat <3h/day, the risk of sitting 3–8 h/day was multiplied by 2.0 (95 CI 1.4 to 2.7) for people working 1–25% in telework, and by 3.0 (1.7 to 5.5) for people working 26–50% in telework; the risk of sitting 8–14 h/day was multiplied by 1.9 (1.2 to 3.0) for people working 1–25% in telework, by 4.7 (2.2 to 9.7) for people working 26–50% in telework, by 4.3 (1.5 to 12.2) for people working 51–75% in telework, and by 9.2 (1.6 to 51.5) for people working >75% in telework; the risk of sitting >14h/day was multiplied by 5.8 (1.07 to 31.5) for people working 51–75% in telework and dramatically increased by 34.3 (3.71 to 317) for people working >75% in telework. Older people, no children, high level of education, long working hours, low physical activity, more time on social media, not working at night and not working on week-end increased the risk of sitting time >3–8 and >8–14 hours. However, the factors that reduced the risk of sitting >14 hours per day were working at night (OR= 0.21, 95 CI 0.06 to 0.74) and doing physical activity >1-2h/week (OR= 0.16, 0.04 to 0.69) and >2-4h/week (OR= 0.13, 0.02 to 0.70) (Fig 5 and S2 Fig).

### Sensitivity analyses

The mean VIF (variance inflation factor) was 2.01, i.e., an acceptable correlation within the predictor variables. All variables had a VIF < 4, except BMI. Removing BMI from our multivariate models did not change our results. Telework in particular had a VIF close to 1 in favor of the robustness of our study (S1 Table).

We further studied the relationship between covariables to evaluate the impact by removing the less answered variables on multivariate regression analysis (i.e., marital status, parenthood, number of inhabitants, and work addiction), the number of observations increased by >20% (n = 1740) and demonstrated similar results (S4 Fig). We also performed step by step regression analyses by adding variables one by one in addition to telework, demonstrating the consistency of the association between sitting time and telework (S2 Table). The association remained also consistent by coding all missing data as a specific class for each variable. To note, coding missing data as a distinct category produced narrower confidence intervals, indicating greater precision and supporting the stability of the estimated association (S5 Fig). With a cut-off at ≥14 hours, the number of workers sitting more than 14 hours increased by 50 people (from 108 sitting >14 hours/d to 158 sitting ≥14 hours/d). Using this cutoff as a sensitivity analysis also gave similar results for all aforementioned analyses. Furthermore, the assessment of non‑response bias through comparison of complete and incomplete respondents showed that the respondents with missing data did not differ from those who fully answered all items, except for sleep quality (Cramer’s V = 0.173) (S3 Table).

## Discussion

The main findings were that telework is the main risk factor for high sedentary behavior. The more you telework, the more you sat – with a strong dose response relationship. Higher education, longer working hours, and spent longer time on social media were associated with an increased sitting time, while working at night, and physical activity regardless the durations decreased sitting time. However, all these variables had weaker association compared to telework.

### Telework as the main risk factor for sedentary behavior

Our study showed the dramatic increase in sedentary behavior with telework, in line with literature [15,34,36–39,57,58]. Similar to those studies, we found that the most sedentary workers were those with the highest percentage of telework [39]. However, those studies had bias in their analysis, particularly by not taking into account most putative influencing variables in their analyses. This study is the first to show that telework was the primary driver of high sedentary behavior when all variables were analyzed together. We also performed several sensitivity analyses in favor of the robustness of our results. The increase of telework, especially during the COVID-19 pandemic, has increased attention to the research on the link between telework and sedentary behavior [59]. A meta-analysis reported a strong increase in sedentary behavior in that period [60], possibly due to longer working hours, online meetings, and less outdoor activities [58]. Our study uses recent data, after the COVID-19 pandemic, showing that sedentary behavior is still a public health concern, worldwide [61]. As the main feature of telework is the use of computers and telecommunications, numerous studies have shown that employees engaged in computer based or desk-based work accumulate great prolonged sitting time during the workday [39,62]. They were more likely had longer sustained sedentary time (bouts >30 minutes) and fewer breaks in sedentary time [62]. Evidence also suggests that remote or home-based work environments may further increase sedentary due to fewer interruptions and longer working hours [39,63]. Therefore, the prolonged sitting time observed among frequent teleworkers in our study aligns with findings on sedentary behavior in office-based populations using objective devices [62,64], where ICT-enabled work is strongly associated with prolonged sitting. Another novelty of our study is the large sample size, that permitted us to assess extreme sedentary behavior such as sitting over 14 hours per day. Some studies reported prevalence of sitting time >11 hours per day [49,50], >12 h/day [52, 65-66–], or 15h/day [52] but those studies did not assess telework. Studies on the influence of telework on sitting time mostly reported more than 8 hours per day sitting [15,38,40,67], and never considered extreme sedentary time such as 14h/day sitting. In our study, prevalence of workers sitting over 14 hours per day rose to 3.2%, 5.4%, and 10.7% among those teleworking over 50%, 75%, or 100%, respectively. However, this group represents only 0.4% of all respondents, which limit the generalizability of this finding. Sedentary behavior is the leading cause of preventable mortality [68]. Sedentary behavior increases the risk of cardiometabolic disease [1,2,69], as well as colon, breast, endometrial, and lung cancers [70–78]. Accordingly, interventions to reduce sedentary behavior among teleworkers include rehabilitation programs [31], height‐adjustable desks at home [39] or portable pedal machines, which may offer health benefits [79]. Nonetheless, the effectiveness of height‑adjustable desks remains debated, as standing often does not exceed sedentary‑level energy expenditure [80,81]. Beyond device‑based approaches, broader strategies addressing environmental, educational/behavioral, and attitudinal determinants may also be important [82] and interventions integrating multi-component and environmental strategy are most effective in reducing sedentary time [83].

### Influence of sociodemographic on prolonged sedentary behavior

Many studies have reported the positive link between older age and higher sitting time [10–13], although some studies reported the opposite [84–86]. Higher sitting time at older age is often attributed to social and environmental factors, chronic diseases, depression, and mobility issues [87]. In our study, age showed associations in univariate analyses, but it was not independently related to sedentary behavior in the multivariate models, suggesting that the crude association was explained by other factors such as occupation or telework. We did not find relationships between sex and sitting time, consistent with most studies summarized in two systematic reviews [26,86]. Although many studies found that women sit more than men [10,31,32], some research from Latin countries [79,80] and high-income European countries [75,81] have reported the opposite. These inconsistencies in the literature likely reflect cultural, occupational, and lifestyle differences across populations. Prevalence of sitting time in our study was also reported higher in Europe (19.2%) compared to Africa (11.2%). This aligns with global evidence showing that high income countries tend to have nearly double the sedentary time of low income countries [88], possibly due to the higher proportion of workers in sedentary occupations [89]. However, region was not associated with sedentary behavior in our multivariate models. As in the previous studies [15,85], we observed that lower education was associated with lower sitting time. This is consistent with the fact that individuals with higher education are more likely to work in tertiary and administrative occupations that involve prolonged sitting [26]. Even during leisure time, if people with lower education engage more in television watching, those with higher education also engage in associated leisure sedentary behavior, although different – such as reading, leisure computer use, or passive travel [90–92]. The fact that people living in megalopolis were more sedentary than people living in smaller city or rural areas has already been reported [84,93], and our descriptive results showed similar patterns. However, region and urbanicity did not remain significant in our multivariate models. Regarding marital status, even though we did not find a relationship with sedentary behavior, a systematic review showed that living in couple increased sitting time [14]. Similarly, while some studies have shown that having children reduces prolonged sitting [26], parenthood was not associated with sedentary behavior in our multivariate analyses, indicating that the crude association was confounded by other variables. Taken together, sociodemographic variables showed limited independent effects once adjusted for all covariates, with only education remaining significant. This highlights that telework is the primary factor associated with prolonged sedentary behavior.

### Occupational variables associated with prolonged sedentary behavior

Occupational characteristics other than telework also influenced prolonged sedentary behavior [94,95]. Office-based workers are likely to be more sedentary [96], spending two-thirds to three quarters of their working hours sitting [7,62] and have fewer breaks as well as fewer bouts of physical activity during working time [62]. They are also more sedentary outside work [7]. Studies reported high levels of sedentary behavior in administrative workers as well as in customer service [17]. However, our multivariate model did not confirm an independent association between sedentary behavior and occupation. Sedentary behavior has also been linked to work-related stress. Although our adjusted analysis did not show an association, previous studies have reported that higher levels of occupational sedentary behavior were significantly associated with mental health problems [97], such as stress [98], sadness and anxiety [99], and psychological distress [38] – possibly related to psychosocial risk factors [100] and lower work engagement [101]. We included work addiction in our analysis. To our knowledge, this association was never explored in the literature. Although this association was not confirmed in multivariate model, the hypothesis remains plausible: work addiction may increase screen-time and reduce opportunities for movement [102,103]. In our multivariate analysis, working at night was a protective factor for extreme sitting time, consistent with a meta-analysis showing that people working at night were less sedentary than daytime workers [104,105]. A possible explanation is that night workers are commonly employed in service and information sectors [106] that require continuous operations, such as healthcare, emergency services, manufacturing, transportation, hospitality, and retail. We also included working on weekends as an occupational variable that tend to be associated with lower sitting time in our multivariate model. While there are few studies on the negative effects of weekend work on sleep disturbance or social interaction [107,108], we did not find any literature exploring its association with sedentary behavior.

### Influence of lifestyle characteristics on prolonged sedentary behavior

Lifestyle factors also showed associations with sedentary behavior. In our study, we included stress at home in our analysis. Even if the direct link between sitting time and stress at home has not been widely explored and no association was found in our multivariate analysis, its association with mental health was often discussed in the literature, especially its relation with greater stress, mood disturbance [109], depression and anxiety [18], as well as mental disorders [19]. However the link between stress and sedentary behavior can be bidirectional where stress possibly as risk factor or a consequence of prolonged sitting [16,110]. Furthermore, we reported a negative association between good sleep quality and high sitting time, consistent with a meta-analysis showing that high sedentary people were more likely to have an increased risk of insomnia and sleep disturbance [28,111]. Again, this relationship maybe bidirectional, poor sleep may reduce energy and motivation for movement, while prolonged sitting increases linked to poor sleep quality [112–114]. Even though our multivariate analysis did not find any association between BMI and sedentary, previous studies reported that abnormal BMI (<18.5 or >25) were more sedentary [24]. It demonstrated behavioral patterns such as increased TV watching among individuals with higher BMI [15,26,115], and gender specific difference in screen time among underweight individuals [91]. This association could be bidirectional with some studies reported the increase risk of obesity due to sedentary [116,117]. Even if our study did not find any link between smoking, alcohol and cannabis consumption with sedentary behavior, several studies have assessed these relations [24,118]. For example, older people who ever smoked were more likely to be sedentary [24], and alcohol and cannabis use were positively associated with sedentary behavior in both adolescents [25] and adults [118]. We confirmed the expected association between higher physical activity and lower sedentary behavior [91], as well as the relationship between sedentary and time spent on social media consistent with studies linking high sedentary time to greater cellphone [26] and screen use [102,103]. Overall, while several lifestyle factors showed association with sitting time, many of these relationships weakened or disappeared in the multivariate models, suggesting that they may be partly explained by other correlated factors. Importantly, telework remained the strongest and most consistent determinants of sedentary behavior.

### Limitations

Our study has some limitations. First, we used a self-reported questionnaire that may have biased estimates due to social desirability, but this limitation is inherent to all studies using self-reported questionnaire and it is the only way to get massive international sample size of respondents. The questionnaire was anonymous [119] and participants were self‑selecting that may create bias, such as individuals who voluntarily participated may have stronger interest, awareness, or personal experience with the topic, leading to over-representation of certain behaviors. In addition, as this is a fully online questionnaire, it could attract more screen user participants [109], precluding the population that are not familiar with internet or screen use. Nonetheless, internet use is nowadays widely common [16]. Some questions were less answered – especially sensitive questions such as BMI, age, mental health condition, or alcohol/smoking/cannabis uses. However, our sensitivity analysis showed that respondents with missing data did not differ from those who fully answered all items except for variable sleep quality. Moreover, we still obtained a large sample size on all variables. Some less answered questions might have promoted under or over-estimation on the data collection [120], but we controlled the robustness of our statistical by including sensitivity analysis. Furthermore, all sensitivity analysis confirmed that the association between sitting time and telework remained consistent across different modeling strategies, including when missing data were explicitly coded as a separate category. Furthermore, sitting time was not measured with objective measurement devices such as accelerometers which may have permitted a more detailed analysis on patterns of sitting time [121,122]. Accuracy of sitting time may be questionable, but literature showed the strength of questionnaire in the measurement of sedentary behavior [123,124]. As the questionnaire on telework only asked “the percentage of telework”, participants could have different interpretation about the concept of teleworking. Fortunately, our study included participants from many different countries with very diverse backgrounds [125]. We have respondents for most continents, even from Africa that is commonly less explored in the literature. However, we lack respondents from Asia, precluding a wide international generalizability of our findings. The cross-sectional design of our study may also preclude to study inferential relationship. Further research should use longitudinal and experimental designs to clarify whether sociodemographic, lifestyle, and occupational factors are true determinants or consequences of sedentary behavior, and specifically to examine how these relationships evolve in teleworking contexts (e.g., different telework intensities, hybrid models, and organizational policies).

## Conclusion

We demonstrated that teleworking is the main risk factor for high sedentary behavior, with a strong dose response relationship. The more you telework, the greater the sitting time. Overall, while several other sociodemographic, occupational, and lifestyle factors showed association with sitting time in univariate model, many of these relationships weakened or disappeared in the multivariate models, except telework that remained the strongest and most consistent determinants of sedentary behavior when concomitantly put together with other variables. Considering the physical and mental health consequences of sedentary behavior, preventive strategies at work should be encouraged to counteract the negative effect of telework on sedentary behavior.

## Supporting information

S1 Fig Factors influencing number of hours of sitting time (univariate linear regression). The effect of each variable on the number of hours of sitting time is represented by a dot on a horizontal line in the forest-plot. The dots represent the effect (coefficient) for each variable, and the length of each line around the dots represent their 95% confidence interval (95 CI). The black solid vertical line represents the null estimate (with a value of 0). Coefficients with horizontal lines that do not cross the null vertical line are significant. Significant variables with a coefficient <0 are factors decreasing sitting time and those with a coefficient >0 are factors increasing sitting time. REF: Reference, i.e., the reference for group comparisons.
(DOCX)

S2 Fig Details of factors influencing the risk of sitting (multivariate logistic regression). The effect of each variable on the risk of sitting >3–8 hours, >8–14 hours, and >14 hours per day is represented by a dot on a horizontal line in the forest-plot. The dots represent the risk of high sitting time (odds ratio) for each variable, and the length of each line around the dots represent their 95% confidence interval (95 CI). The black solid vertical line represents the null estimate (with a value of 1). Odds ratio with horizontal lines that do not cross the vertical line are significant. Significant variables with an odds ratio <1 are protective factors decreasing the risk if high sitting time and those with an odds ratio >1 are risk factors. REF: Reference, i.e., the reference for group comparisons.
(DOCX)

S3 Fig Summary and details of factors influencing the risk of sitting (univariate logistic regression). The effect of each variable on the risk of sitting >3–8 hours, >8–14 hours, and >14 hours per day is represented by a dot on a horizontal line in the forest-plot. The dots represent the risk of high sitting time (odds ratio) for each variable, and the length of each line around the dots represent their 95% confidence interval (95 CI). The black solid vertical line represents the null estimate (with a value of 1). Odds ratio with horizontal lines that do not cross the vertical line are significant. Significant variables with an odds ratio <1 are protective factors decreasing the risk if high sitting time and those with an odds ratio >1 are risk factors. REF: Reference, i.e., the reference for group comparisons.
(DOCX)

S4 Fig Sensitivity analyses: factors influencing number of hours of sitting time (multivariate linear regression), with the removal of the less answered variables, i.e., marital status, parenthood, number of inhabitants, and work addiction. The effect of each variable on the number of hours of sitting time is represented by a dot on a horizontal line in the forest-plot. The dots represent the effect (coefficient) for each variable, and the length of each line around the dots represent their 95% confidence interval (95 CI). The black solid vertical line represents the null estimate (with a value of 0). Coefficients with horizontal lines that do not cross the null vertical line are significant. Significant variables with a coefficient <0 are factors decreasing sitting time and those with a coefficient >0 are factors increasing sitting time. REF: Reference, i.e., the reference for group comparisons.
(DOCX)

S5 Fig Sensitivity analyses: linear and logistic regression analyses comparing model 1 (only the outcome “sitting” and exposure to “telework”), model 22 (final model with all covariates), and model 23 (final model 22 with missing data as a specific class for all covariates), demonstrating the consistency of the association between sitting time and telework. To note, model 23 in which missing data were treated as a specific category for all covariates, yielded narrower confidence intervals, thereby strengthening the robustness of the association between sitting time and telework.
(DOCX)

S1 Table Variance Inflation Factor (VIF) test. VIF test is used to check the presence of multicollinearity that could affect the accuracy of the regression estimates. VIF = 1: no correlation between the predictor variable and other variables. 1 < VIF < 5: moderate correlation; generally acceptable. VIF ≥ 5: indicates potentially problematic multicollinearity. VIF ≥ 10: Indicates serious multicollinearity that may require further investigation.
(DOCX)

S2 Table Sensitivity analyses: step by step regression analyses by adding variables one by one in addition to telework, demonstrating the consistency of the association between sitting time and telework. (DOCX)

S3 Table Sensitivity analyses: Assessment of non‑response bias among the 2904 participants who responded both to sitting time and telework items (comparisons between the 1417 participants who responded to all questions without any missing data, and the 1487 respondents who did not respond to at least one question). Complete dataset uses sample in the final multivariate analysis model (Model 22). Cramer’s V ≥0.10 were considered small (*), ≥0.15 moderate (**), and ≥0.25 strong (***). Stars show the highest significance between two groups. REF: Reference, i.e., the reference for group comparisons.
(DOCX)

S1 File STROBE Statement – Checklist of items that should be included in reports of cross-sectional studies. (DOCX)

S1 Dataset Teleworking as the main risk factor for sedentary behavior: the COVISTRESS international study. (XLS)
