# Supplementary material for: Teleworking as the main risk factor for sedentary behavior: the COVISTRESS international study
Source: PLoS One. 2026 Sep 10;21(9):e0356764. doi: 10.1371/journal.pone.0356764 (PMC13561329; doi:10.1371/journal.pone.0356764)
Supplement: Supplementary file 1 [file pone.0356764.s001.docx]

**S1 Fig**. Factors influencing the risk of sitting >3 to 8 hours, >8 to 14 hours, and >14 hours per day (univariate linear regression).

*The effect of each variable on the number of hours of sitting time is represented by a dot on a horizontal line in the forest-plot. The dots represent the effect (coefficient) for each variable, and the length of each line around the dots represent their 95% confidence interval (95CI). The black solid vertical line represents the null estimate (with a value of 0). Coefficients with horizontal lines that do not cross the null vertical line are significant. Significant variables with a coefficient <0 are factors decreasing sitting time and those with a coefficient >0 are factors increasing sitting time. REF: Reference i.e. the reference for group comparisons.*
