# Supplementary material for: Teleworking as the main risk factor for sedentary behavior: the COVISTRESS international study
Source: PLoS One. 2026 Sep 10;21(9):e0356764. doi: 10.1371/journal.pone.0356764 (PMC13561329; doi:10.1371/journal.pone.0356764)
Supplement: Supplementary file 2 [file pone.0356764.s002.docx]

**S2 Fig**. Details of factors influencing the risk of sitting >3 to 8 hours, >8 to 14 hours, and >14 hours per day (multivariate logistic regression).

*The effect of each variable on the risk of sitting >3 to 8 hours, >8 to 14 hours, and >14 hours per day is represented by a dot on a horizontal line in the forest-plot. The dots represent the risk of high sitting time (odds ratio) for each variable, and the length of each line around the dots represent their 95% confidence interval (95CI). The black solid vertical line represents the null estimate (with a value of 1). Odds ratio with horizontal lines that do not cross the vertical line are significant. Significant variables with an odds ratio <1 are protective factors decreasing the risk if high sitting time and those with an odds ratio >1 are risk factors. REF: Reference i.e. the reference for group comparisons.*
