# Supplementary material for: Teleworking as the main risk factor for sedentary behavior: the COVISTRESS international study
Source: PLoS One. 2026 Sep 10;21(9):e0356764. doi: 10.1371/journal.pone.0356764 (PMC13561329; doi:10.1371/journal.pone.0356764)
Supplement: Supplementary file 5 [file pone.0356764.s005.docx]

**S5 Fig**. Sensitivity analyses: linear and logistic regression analyses comparing model 1 (only the outcome “sitting” and exposure to “telework”), model 22 (final model with all covariates), and model 23 (final model 22 with missing data as a specific class for all covariates), demonstrating the consistency of the association between sitting time and telework.

*To note, model 23 in which missing data were treated as a specific category for all covariates, yielded narrower confidence intervals, thereby strengthening the robustness of the association between sitting time and telework.*
