# Supplementary material for: Teleworking as the main risk factor for sedentary behavior: the COVISTRESS international study
Source: PLoS One. 2026 Sep 10;21(9):e0356764. doi: 10.1371/journal.pone.0356764 (PMC13561329; doi:10.1371/journal.pone.0356764)
Supplement: Supplementary file 6 [file pone.0356764.s006.docx]

**S1 Table**. Variance Inflation Factor (VIF) test to check the presence of multicollinearity that could affect the accuracy of the regression estimates.

*VIF = 1: no correlation between the predictor variable and other variables. 1 < VIF < 5: moderate correlation; generally acceptable. VIF ≥ 5: indicates potentially problematic multicollinearity. VIF ≥ 10: Indicates serious multicollinearity that may require further investigation.*

| **Variable** | **VIF** |
| --- | --- |
| BMI (underweight) | 8.97 |
| BMI (normal) | 7.96 |
| BMI (overweight) | 5.05 |
| Education (doctoral) | 3.46 |
| Education (master) | 3.27 |
| Education (undergraduate) | 2.89 |
| Social media (>1h-3h/day) | 2.61 |
| Working hours (>30-40h/week) | 2.27 |
| Region (Africa) | 2.12 |
| Working hours (>40-50h/week) | 2.08 |
| Social media (>20min-1h/day) | 2.03 |
| Physical activity (1-2h/week) | 1.80 |
| Physical activity (1h/week) | 1.80 |
| Social media (≤20min/day) | 1.79 |
| Physical activity (>2-4h/week) | 1.75 |
| Occupation (employee) | 1.74 |
| Working hours (>50h/week) | 1.72 |
| Stress at work (moderate) | 1.61 |
| Stress at work (weak) | 1.60 |
| Parenthood (having children) | 1.58 |
| Working at night (yes) | 1.57 |
| Physical activity (≤30min/week) | 1.54 |
| Working on weekend (yes) | 1.52 |
| Age (>42 years) | 1.42 |
| Occupation (intermediary profession) | 1.41 |
| Stress at home (weak) | 1.40 |
| Number of inhabitants (>5000-1 million) | 1.37 |
| Sex (men) | 1.37 |
| Number of inhabitants (≤5000) | 1.35 |
| Marital status (in relationship) | 1.30 |
| Stress at home (moderate) | 1.25 |
| Region (America) | 1.23 |
| Smoking (yes) | 1.20 |
| Telework (0%) | 1.20 |
| Cannabis (yes) | 1.16 |
| Work addiction (moderate) | 1.16 |
| Alcohol (yes) | 1.14 |
| Occupation (others) | 1.13 |
| Work addiction (weak) | 1.13 |
| Telework (1-25%) | 1.11 |
| Sleep quality (good) | 1.10 |
| Telework (51-75%) | 1.07 |
| Telework (26-50%) | 1.07 |
| Region (Asia Pacific) | 1.04 |
| **Mean VIF** | **2.01** |
