# Supplementary material for: Teleworking as the main risk factor for sedentary behavior: the COVISTRESS international study
Source: PLoS One. 2026 Sep 10;21(9):e0356764. doi: 10.1371/journal.pone.0356764 (PMC13561329; doi:10.1371/journal.pone.0356764)
Supplement: Supplementary file 7 [file pone.0356764.s007.docx]

**S2 Table**. Sensitivity analyses: step by step linear regression analyses by adding variables one by one in addition to telework, demonstrating the consistency of the association between sitting time and telework.

| **Factors influencing …** | **… the number of hours of sitting as a quantitative variable** | | | | | | | | | | | | | | | | | | | | | |
| --- | --- | --- | --- | --- | --- | --- | --- | --- | --- | --- | --- | --- | --- | --- | --- | --- | --- | --- | --- | --- | --- | --- |
|  | **Model**  **1**  Telework | **Model**  **2**  Model  1  +  age | **Model**  **3**  Model  2  +  sex | **Model**  **4**  Model  3  +  marital status | **Model**  **5**  Model  4  +  parent-hood | **Model**  **6**  Model  5  +  educa-tion level | **Model**  **7**  Model  6  +  region | **Model**  **8**  Model  7  +  number of inha-bitants | **Model**  **9**  Model  8  +  occupation | **Model**  **10**  Model  9  +  working time | **Model**  **11**  Model  10  +  work addiction | **Model**  **12**  Model  11  +  working at night | **Model**  **13**  Model  12  +  working on week-end | **Model**  **14**  Model  13  +  stress at work | **Model**  **15**  Model  14  +  stress at home | **Model**  **16**  Model  15  +  alcohol | **Model**  **17**  Model  16  +  smoking | **Model**  **18**  Model  17  +  cannabis | **Model**  **19**  Model  18  +  sleep quality | **Model**  **20**  Model  19  +  physical activity | **Model**  **21**  Model  20  +  BMI | **Model**  **22**  Model  21  +  time on social media |
|  | n=2,904 | n=2,790 | n=2,777 | n=2,606 | n=2,472 | n=2,462 | n=2,379 | n=2,172 | n=2,172 | n=2,161 | n=1,794 | n=1,693 | n=1,675 | n=1,605 | n=1,578 | n=1,570 | n=1,566 | n=1,557 | n=1,557 | n=1,548 | n=1,492 | n=1,417 |
| **Percentage of telework** |  |  |  |  |  |  |  |  |  |  |  |  |  |  |  |  |  |  |  |  |  |  |
| 0% | REF |  |  |  |  |  |  |  |  |  |  |  |  |  |  |  |  |  |  |  |  |  |
| 1-25% | **<0.001** | **<0.001** | **<0.001** | **<0.001** | **<0.001** | **<0.001** | **<0.001** | **<0.001** | **<0.001** | **<0.001** | **<0.001** | **0.001** | **0.002** | **0.003** | **0.003** | **0.008** | **0.007** | **0.008** | **0.009** | **0.004** | **0.012** | **0.014** |
| 26-50% | **<0.001** | **<0.001** | **<0.001** | **<0.001** | **<0.001** | **<0.001** | **<0.001** | **<0.001** | **<0.001** | **<0.001** | **<0.001** | **<0.001** | **<0.001** | **<0.001** | **<0.001** | **<0.001** | **<0.001** | **<0.001** | **<0.001** | **<0.001** | **<0.001** | **<0.001** |
| 51-75% | **<0.001** | **<0.001** | **<0.001** | **<0.001** | **<0.001** | **<0.001** | **<0.001** | **<0.001** | **<0.001** | **<0.001** | **<0.001** | **<0.001** | **<0.001** | **0.001** | **0.001** | **0.001** | **0.001** | **0.001** | **0.001** | **0.001** | **<0.001** | **<0.001** |
| >75% | **<0.001** | **<0.001** | **<0.001** | **<0.001** | **<0.001** | **<0.001** | **<0.001** | **<0.001** | **<0.001** | **<0.001** | **<0.001** | **<0.001** | **<0.001** | **<0.001** | **<0.001** | **<0.001** | **<0.001** | **<0.001** | **<0.001** | **<0.001** | **<0.001** | **<0.001** |
| **Age** |  | **<0.001** | **0.001** | **0.003** | **0.002** | **0.002** | **0.008** | **0.018** | **0.028** | **0.018** | **0.090** | 0.14 | 0.22 | 0.17 | 0.15 | 0.11 | 0.13 | 0.10 | 0.11 | 0.13 | 0.22 | 0.12 |
| **Sex** |  |  | 0.29 | 0.26 | 0.27 | 0.14 | 0.29 | 0.46 | 0.38 | 0.24 | 0.35 | 0.33 | 0.36 | 0.48 | 0.48 | 0.36 | 0.40 | 0.39 | 0.40 | 0.38 | 0.46 | 0.44 |
| **Marital status** |  |  |  | 1.00 | 0.51 | 0.78 | 0.84 | 0.75 | 0.74 | 0.71 | 0.75 | 0.60 | 0.52 | 0.48 | 0.61 | 0.54 | 0.57 | 0.60 | 0.61 | 0.77 | 0.99 | 0.81 |
| **Parenthood** |  |  |  |  | 0.16 | 0.48 | 0.37 | 0.58 | 0.45 | 0.41 | 0.84 | 0.64 | 0.61 | 0.59 | 0.60 | 0.66 | 0.68 | 0.61 | 0.61 | 0.49 | 0.32 | 0.51 |
| **Education level** |  |  |  |  |  |  |  |  |  |  |  |  |  |  |  |  |  |  |  |  |  |  |
| ≤ Highschool |  |  |  |  |  |  |  |  |  |  |  |  |  |  |  |  |  |  |  |  |  |  |
| Undergraduate |  |  |  |  |  | 0.81 | 0.74 | 0.92 | 0.68 | 0.49 | 0.35 | 0.42 | 0.50 | 0.50 | 0.63 | 0.56 | 0.61 | 0.61 | 0.54 | 0.76 | 0.62 | 0.67 |
| Master degree |  |  |  |  |  | **0.003** | **0.008** | **0.005** | 0.091 | 0.15 | 0.24 | 0.40 | 0.34 | 0.26 | 0.17 | 0.18 | 0.16 | 0.17 | 0.17 | 0.10 | 0.13 | 0.16 |
| Doctoral degree |  |  |  |  |  | **0.002** | **0.009** | **0.014** | 0.21 | 0.30 | 0.27 | 0.14 | 0.12 | 0.10 | 0.074 | 0.069 | **0.047** | **0.041** | **0.045** | **0.025** | **0.036** | **0.032** |
| **Region** |  |  |  |  |  |  |  |  |  |  |  |  |  |  |  |  |  |  |  |  |  |  |
| Europe |  |  |  |  |  |  |  |  |  |  |  |  |  |  |  |  |  |  |  |  |  |  |
| Africa |  |  |  |  |  |  | 0.97 | 0.25 | 0.19 | 0.35 | 0.66 | 0.33 | 0.16 | 0.17 | 0.20 | 0.15 | 0.11 | 0.077 | 0.078 | 0.16 | 0.40 | 0.63 |
| Asia Pacific |  |  |  |  |  |  | 0.23 | 0.31 | 0.30 | 0.29 | 1.00 | 0.89 | 0.87 | 0.85 | 0.86 | 0.83 | 0.89 | 0.87 | 0.80 | 0.80 | 0.88 | 0.85 |
| America |  |  |  |  |  |  | 0.46 | 0.22 | 0.25 | 0.33 | 0.50 | 0.96 | 0.72 | 0.79 | 0.83 | 0.83 | 0.86 | 0.89 | 0.88 | 0.90 | 0.98 | 0.96 |
| **Number of inhabitants** |  |  |  |  |  |  |  |  |  |  |  |  |  |  |  |  |  |  |  |  |  |  |
| ≤5000 |  |  |  |  |  |  |  |  |  |  |  |  |  |  |  |  |  |  |  |  |  |  |
| >5000-1 million |  |  |  |  |  |  |  | **0.027** | **0.033** | 0.064 | 0.17 | 0.29 | 0.22 | 0.35 | 0.50 | 0.54 | 0.54 | 0.49 | 0.47 | 0.44 | 0.55 | 0.92 |
| >1 million |  |  |  |  |  |  |  | 0.10 | **0.097** | 0.092 | 0.87 | 0.94 | 0.86 | 0.67 | 0.56 | 0.57 | 0.58 | 0.66 | 0.63 | 0.58 | 0.61 | 0.46 |
| **Occupation** |  |  |  |  |  |  |  |  |  |  |  |  |  |  |  |  |  |  |  |  |  |  |
| Superior /intellectual |  |  |  |  |  |  |  |  |  |  |  |  |  |  |  |  |  |  |  |  |  |  |
| Intermediary profession |  |  |  |  |  |  |  |  | 0.13 | 0.19 | 0.81 | 0.96 | 0.90 | 0.87 | 0.82 | 0.94 | 0.91 | 0.85 | 0.87 | 0.87 | 0.97 | 0.79 |
| Employee |  |  |  |  |  |  |  |  | **0.078** | 0.15 | 0.14 | 0.14 | 0.18 | 0.22 | 0.28 | 0.28 | 0.28 | 0.32 | 0.29 | 0.30 | 0.11 | 0.088 |
| Others |  |  |  |  |  |  |  |  | 0.29 | 0.39 | 0.40 | 0.095 | 0.15 | 0.095 | 0.11 | 0.10 | 0.11 | 0.10 | 0.10 | 0.12 | 0.17 | 0.15 |
| **Working time (per week)** |  |  |  |  |  |  |  |  |  |  |  |  |  |  |  |  |  |  |  |  |  |  |
| ≤30h |  |  |  |  |  |  |  |  |  |  |  |  |  |  |  |  |  |  |  |  |  |  |
| >30-40h |  |  |  |  |  |  |  |  |  | **0.015** | 0.10 | 0.24 | 0.29 | 0.48 | 0.43 | 0.41 | 0.41 | 0.33 | 0.33 | 0.31 | 0.37 | 0.17 |
| >40-45h |  |  |  |  |  |  |  |  |  | **<0.001** | **0.005** | **0.003** | **0.002** | **0.008** | **0.007** | **0.013** | **0.013** | **0.009** | **0.011** | **0.016** | **0.019** | **0.007** |
| >50h |  |  |  |  |  |  |  |  |  | **0.019** | 0.28 | 0.082 | 0.064 | 0.12 | 0.11 | 0.14 | 0.14 | 0.11 | 0.12 | 0.11 | 0.12 | 0.055 |
| **Work addiction** |  |  |  |  |  |  |  |  |  |  |  |  |  |  |  |  |  |  |  |  |  |  |
| Weak |  |  |  |  |  |  |  |  |  |  |  |  |  |  |  |  |  |  |  |  |  |  |
| Moderate |  |  |  |  |  |  |  |  |  |  | 0.42 | 0.39 | 0.43 | 0.42 | 0.43 | 0.36 | 0.36 | 0.35 | 0.33 | 0.44 | 0.70 | 0.62 |
| High |  |  |  |  |  |  |  |  |  |  | 0.26 | 0.44 | 0.35 | 0.25 | 0.34 | 0.27 | 0.29 | 0.29 | 0.29 | 0.31 | 0.34 | 0.35 |
| **Working at night** |  |  |  |  |  |  |  |  |  |  |  | **<0.001** | **<0.001** | **<0.001** | **<0.001** | **<0.001** | **<0.001** | **<0.001** | **<0.001** | **<0.001** | **<0.001** | **<0.001** |
| **Working on weekend** |  |  |  |  |  |  |  |  |  |  |  |  | **0.012** | **0.042** | **0.045** | **0.041** | **0.032** | **0.022** | **0.025** | **0.042** | **0.032** | 0.059 |
| **Stress at work** |  |  |  |  |  |  |  |  |  |  |  |  |  |  |  |  |  |  |  |  |  |  |
| Weak |  |  |  |  |  |  |  |  |  |  |  |  |  |  |  |  |  |  |  |  |  |  |
| Moderate |  |  |  |  |  |  |  |  |  |  |  |  |  | 0.92 | 0.88 | 0.89 | 0.93 | 0.99 | 0.89 | 0.86 | 0.84 | 0.89 |
| High |  |  |  |  |  |  |  |  |  |  |  |  |  | 0.11 | 0.33 | 0.34 | 0.30 | 0.31 | 0.44 | 0.63 | 0.87 | 0.43 |
| **Stress at home** |  |  |  |  |  |  |  |  |  |  |  |  |  |  |  |  |  |  |  |  |  |  |
| Weak |  |  |  |  |  |  |  |  |  |  |  |  |  |  |  |  |  |  |  |  |  |  |
| Moderate |  |  |  |  |  |  |  |  |  |  |  |  |  |  | 0.81 | 0.75 | 0.71 | 0.67 | 0.55 | 0.52 | 0.51 | 0.58 |
| High |  |  |  |  |  |  |  |  |  |  |  |  |  |  | **0.046** | 0.053 | 0.056 | 0.055 | 0.078 | 0.22 | 0.21 | 0.46 |
| **Alcohol** |  |  |  |  |  |  |  |  |  |  |  |  |  |  |  | 0.18 | 0.38 | 0.48 | 0.48 | 0.53 | 0.51 | 0.27 |
| **Smoking** |  |  |  |  |  |  |  |  |  |  |  |  |  |  |  |  | 0.070 | 0.14 | 0.17 | 0.22 | 0.26 | 0.15 |
| **Cannabis** |  |  |  |  |  |  |  |  |  |  |  |  |  |  |  |  |  | 0.14 | 0.15 | 0.46 | 0.45 | 0.68 |
| **Sleep quality** |  |  |  |  |  |  |  |  |  |  |  |  |  |  |  |  |  |  | 0.051 | 0.074 | 0.11 | 0.17 |
| **Physical activity (per week)** |  |  |  |  |  |  |  |  |  |  |  |  |  |  |  |  |  |  |  |  |  |  |
| ≤30min |  |  |  |  |  |  |  |  |  |  |  |  |  |  |  |  |  |  |  |  |  |  |
| >30min-1h |  |  |  |  |  |  |  |  |  |  |  |  |  |  |  |  |  |  |  | **0.018** | **0.024** | **0.022** |
| >1h-2h |  |  |  |  |  |  |  |  |  |  |  |  |  |  |  |  |  |  |  | **<0.001** | **<0.001** | **<0.001** |
| >2h-4h |  |  |  |  |  |  |  |  |  |  |  |  |  |  |  |  |  |  |  | **0.003** | **0.005** | **0.015** |
| >4h |  |  |  |  |  |  |  |  |  |  |  |  |  |  |  |  |  |  |  | **0.019** | **0.014** | **0.012** |
| **Body mass index** |  |  |  |  |  |  |  |  |  |  |  |  |  |  |  |  |  |  |  |  |  |  |
| Underweight |  |  |  |  |  |  |  |  |  |  |  |  |  |  |  |  |  |  |  |  |  |  |
| Normal |  |  |  |  |  |  |  |  |  |  |  |  |  |  |  |  |  |  |  |  | 0.82 | 0.81 |
| Overweight |  |  |  |  |  |  |  |  |  |  |  |  |  |  |  |  |  |  |  |  | 0.66 | 0.75 |
| Obesity |  |  |  |  |  |  |  |  |  |  |  |  |  |  |  |  |  |  |  |  | 0.43 | 0.57 |
| **Time spent on social media** |  |  |  |  |  |  |  |  |  |  |  |  |  |  |  |  |  |  |  |  |  |  |
| ≤20min |  |  |  |  |  |  |  |  |  |  |  |  |  |  |  |  |  |  |  |  |  |  |
| >20min-1h |  |  |  |  |  |  |  |  |  |  |  |  |  |  |  |  |  |  |  |  |  | **0.009** |
| >1h-3h |  |  |  |  |  |  |  |  |  |  |  |  |  |  |  |  |  |  |  |  |  | 0.79 |
| >3h |  |  |  |  |  |  |  |  |  |  |  |  |  |  |  |  |  |  |  |  |  | **<0.001** |
