# Supplementary material for: Teleworking as the main risk factor for sedentary behavior: the COVISTRESS international study
Source: PLoS One. 2026 Sep 10;21(9):e0356764. doi: 10.1371/journal.pone.0356764 (PMC13561329; doi:10.1371/journal.pone.0356764)
Supplement: Supplementary file 8 [file pone.0356764.s008.docx]

**S3 Table**. Sensitivity analyses: Assessment of non‑response bias among the 2904 participants who responded both to sitting time and telework items (comparisons between the 1417 participants who responded to all questions without any missing data, and the 1487 respondents who did not respond to at least one question)

*Complete dataset uses sample in the final multivariate analysis model (Model 22). Cramer’s V ≥0.10 were considered small (*), ≥0.15 moderate (**), and ≥0.25 strong (***). Stars show the highest significance between two groups. REF: Reference i.e. the reference for group comparisons.*

|  | Among the 2904 participants  who responded both to “sitting time” and “telework” items | | |
| --- | --- | --- | --- |
|  | Incomplete dataset  n=1487 | Complete dataset  n=1417 | Cramer’s V |
| **Percentage of telework** |  |  |  |
| 0% | 895 (60.2%) | 897 (63.3%) | 0.090 |
| 1-25% | 334 (22.5%) | 336 (23.7%) |  |
| 26-50% | 114 (7.7%) | 111 (7.8%) |  |
| 51-75% | 77 (5.2%) | 47 (3.3%) |  |
| >75% | 67 (4.5%) | 26 (1.8%) |  |
| **Age** |  |  |  |
| ≤42 | 929 (67.7%) | 927 (65.4%) | 0.024 |
| >42 | 444 (32.3%) | 490 (34.6%) |  |
| **Sex** |  |  |  |
| Women | 819 (55.6%) | 794 (56.0%) | 0.004 |
| Men | 653 (44.4%) | 623 (44.0%) |  |
| **Marital status** |  |  |  |
| In relationship | 867 (66.0%) | 983 (69.4%) | 0.036 |
| Others | 447 (34.0%) | 434 (30.6%) |  |
| **Parenthood** |  |  |  |
| No child | 405 (34.4%) | 512 (36.1%) | 0.018 |
| ≥1 child | 771 (65.6%) | 905 (63.9%) |  |
| **Education level** |  |  |  |
| ≤ Highschool | 211 (14.3%) | 165 (11.6%) | 0.081 |
| Undergraduate | 486 (32.9%) | 445 (31.4%) |  |
| Master degree | 368 (24.9%) | 452 (31.9%) |  |
| Doctoral degree | 411 (27.9%) | 355 (25.1%) |  |
| **Region** |  |  |  |
| Europe | 649 (47.2%) | 774 (54.6%) | 0.089 |
| Africa | 614 (44.7%) | 520 (36.7%) |  |
| Asia Pacific | 7 (0.5%) | 2 (0.1%) |  |
| America | 104 (7.6%) | 121 (8.5%) |  |
| **Number of inhabitants** |  |  |  |
| ≤5000 | 248 (22.9%) | 305 (21.5%) | 0.069 |
| >5000-1 million | 728 (67.1%) | 1019 (71.9%) |  |
| >1 million | 109 (10.1%) | 93 (6.6%) |  |
| **Occupation** |  |  |  |
| Superior /intellectual | 779 (52.4%) | 762 (53.8%) | 0.034 |
| Intermediary profession | 283 (19.0%) | 283 (20.0%) |  |
| Employee | 372 (25.0%) | 315 (22.2%) |  |
| Others | 53 (3.6%) | 57 (4.0%) |  |
| **Working time (per week)** |  |  |  |
| ≤30h | 285 (19.4%) | 235 (16.6%) | 0.048 |
| >30-40h | 601 (40.9%) | 628 (44.3%) |  |
| >40-50h | 385 (26.2%) | 384 (27.1%) |  |
| >50h | 199 (13.5%) | 170 (12.0%) |  |
| **Work addiction** |  |  |  |
| Weak | 538 (58.0%) | 879 (62.0%) | 0.072 |
| Moderate | 254 (27.4%) | 398 (28.1%) |  |
| High | 135 (14.6%) | 140 (9.9%) |  |
| **Working at night** |  |  |  |
| No | 789 (61.1%) | 900 (63.5%) | 0.025 |
| Yes | 502 (38.9%) | 517 (36.5%) |  |
| **Working on weekend** |  |  |  |
| No | 589 (41.5%) | 574 (40.5%) | 0.010 |
| Yes | 830 (58.5%) | 843 (59.5%) |  |
| **Stress at work** |  |  |  |
| Weak | 344 (31.3%) | 505 (35.6%) | 0.050 |
| Moderate | 549 (49.9%) | 683 (48.2%) |  |
| High | 207 (18.8%) | 229 (16.2%) |  |
| **Stress at home** |  |  |  |
| Weak | 533 (49.0%) | 727 (51.3%) | 0.094 |
| Moderate | 434 (39.9%) | 606 (42.8%) |  |
| High | 121 (11.1%) | 84 (5.9%) |  |
| **Alcohol** |  |  |  |
| 0 | 1299 (88.9%) | 1253 (88.4%) | 0.008 |
| ≥ 1 | 162 (11.1%) | 164 (11.6%) |  |
| **Smoking** |  |  |  |
| 0 | 1285 (87.9%) | 1205 (85.0%) | 0.042 |
| ≥ 1 | 177 (12.1%) | 212 (15.0%) |  |
| **Cannabis** |  |  |  |
| 0 | 1408 (97.5%) | 1369 (96.6%) | 0.027 |
| ≥ 1 | 36 (2.5%) | 48 (3.4%) |  |
| **Sleep quality** |  |  |  |
| Bad quality | 650 (43.7%) | 865 (61.0%) | 0.173** |
| Good quality | 837 (56.3%) | 552 (39.0%) |  |
| **Physical activity (per week)** |  |  |  |
| ≤30min | 323 (22.8%) | 274 (19.3%) | 0.045 |
| >30min-1h | 203 (14.3%) | 199 (14.0%) |  |
| >1h-2h | 318 (22.4%) | 344 (24.3%) |  |
| >2h-4h | 327 (23.0%) | 333 (23.5%) |  |
| >4h | 249 (17.5%) | 267 (18.8%) |  |
| **Body mass index** |  |  |  |
| Underweight | 48 (4.1%) | 43 (3.0%) | 0.055 |
| Normal | 574 (49.1%) | 752 (53.1%) |  |
| Overweight | 349 (29.9%) | 425 (30.0%) |  |
| Obesity | 198 (16.9%) | 197 (13.9%) |  |
| **Time spent on social media** |  |  |  |
| ≤20min | 167 (15.7%) | 249 (17.6%) | 0.059 |
| >20min-1h | 248 (23.4%) | 266 (18.8%) |  |
| >1h-3h | 236 (22.2%) | 347 (24.5%) |  |
| >3h | 411 (38.7%) | 555 (39.2%) |  |
