# Supplementary material for: Teleworking as the main risk factor for sedentary behavior: the COVISTRESS international study
Source: PLoS One. 2026 Sep 10;21(9):e0356764. doi: 10.1371/journal.pone.0356764 (PMC13561329; doi:10.1371/journal.pone.0356764)
Supplement: Supplementary file 10 [file pone.0356764.s011.docx]

**PLOS One**

**Teleworking as the main risk factor for sedentary behavior: the COVISTRESS**

**international study**

Dear authors

It has been a pleasure to have the opportunity to review the manuscript, “Teleworking as the main risk factor for sedentary behavior: the COVISTRESS’ international study”. As the debate on whether teleworking should or shouldn’t be allowed to continue rumbles on, this study provides an interesting insight into the potential health impacts of teleworking. It is clear that a lot of effort has gone into the study. Nonetheless, as you will see from my notes below, I do have quite a few comments. These are not meant to dishearten. I hope they will lead to improvements in the clarity, readability, and reproducibility of the study, and following this I hope the study will receive a wider audience and further the discussions about teleworking – and how to improve worker health and wellbeing.

Introduction

1. Please would the authors consider providing a definition of teleworking. In case it’s useful, here is the link to Eurofound’s definition: <https://www.eurofound.europa.eu/en/topics/teleworking>
2. To save the reader having to look up every reference, it would be helpful if the authors were more explicit in their storytelling. For example:

The authors state, “However, a major recent occupational change is related to teleworking, that could also be particularly impacting on sedentary behavior”. If the authors mean the Covid-19 pandemic then please will they say so?

The authors state, “A study quantifying the percentage of telework concluded that the workers with the highest percentage (76%-100% of time spent working) had the highest sedentary time compared with other groups (1%-25%, and 26%-50%).” Please will the authors state who the other groups were?

3. To improve clarity, please would the authors explain the following:

What do they mean by “weight” in their statement, “While aforementioned studies … only a few of them concomitantly assessed the weight of telework on sitting time in addition to …”? Whilst at this point I haven’t thoroughly read the manuscript in full, it appears they’ve reported effect size (using Cohen’s D, Cramer’s V, Odds Ratios, etc). Does their study assess weight or effect size?

Do the authors mean one country, and if so, which country in their statement, “Moreover, those studies focused mainly on one country.”?

What “other components” are relevant to the authors’ study in addition to the “many variables”, when they state, “As well as other components (26–28), sedentary behavior can be influenced by many variables (11,12,29)”?

4. The authors state, “However, no studies took into account all those variables simultaneously in the statistical models investigating a link between sedentary time and telework.” Please would the authors explain more fully what the studies have accounted for, and what in their opinion has been missed? Could the lack of simultaneous inclusion of variables in previous studies be due to potential problems relating to the danger of overfitting, multicollinearity, biased causal estimates etc? Could it be due to data availability? Could the previous studies have decided for theoretical and/or statistical reasons not to include some variables?

Methods: Study Design

5. Whilst the authors do state that “Participation was entirely voluntary and anonymous”, it would be helpful if they would also explain if participants are self-selecting and if so, how this might bias the results (and to address this too in the section on limitations).

Methods: Participants and also Results: Participants

6. The authors state, “We included all participants who answered their sitting time (Figure 1).” Please would the authors double check the data shown in this figure? It shows the number of participants was reduced from 7064 to 6653 by excluding 1333 who did not respond to the question about sitting time. 7064 minus 1333 equals 5,731 not 6653.
7. Figure 1 shows there were 2,904 participants who responded to the question about telework. As the aim of the study was to take into account all the variables (which can influence sedentary behaviour) “simultaneously in the statistical models investigating a link between sedentary time and telework” was the final number of participants in the study 2,904 or did the authors do some form of missing data imputation?
8. Figure 1 also includes the number of responses per variable. What was the pattern of missing data? Was there any imputation? What was the final analytical sample?
9. The authors state that the “teleworking variables were applicable only to this subgroup”. What exactly were the teleworking variables – what were respondents asked?

Methods: Outcomes Instrument Survey

10. The inclusion of the word “Outcomes” suggest that this will focus only on the outcome – sedentary behaviour. However, this section covers more than that. For reader clarity, it might be helpful to replace “Outcomes Instrument Survey” with something along the lines of “Survey variables”; “Main outcomes” with “exposure and outcome variables” or “dependent and independent variables”; and “secondary outcomes” with “covariates”.
11. Please would the authors explain for the readers, why they treated their outcome variable, sitting time per day, as both a quantitative and qualitative variable, and what was their rationale for determining the thresholds for each category? Furthermore, which category are participants assigned to if they sit for 8 hours per day, is it 3-8 or 8-14; or if they sit for 14 hours per day is it 8-14, or ≥14?
12. Please would the authors explain why they treated their exposure variable, teleworking, as a categorical variable, and what was their rationale for determining the thresholds for each category? Furthermore, which category are participants assigned to if they telework 25%, 50% and 75% when the potential categories are 1-25%, 25-50%, 50-75%, 75-99%?
13. Please would the authors explain the rationale for determining the thresholds for the categories of the “secondary” variables? Furthermore, as several of the thresholds overlap, which category are participants assigned to in the following scenarios: 1 million inhabitants; weekly working hours of 40hrs, and 50 hrs; physical activity of 1hr, and 2hrs; BMI of 25; and social media of 20mins, and 1 hour?
14. In addition, please would the authors explain the rationale for determining the reference categories in these variables. For example, why select working less than 30 hours per week as the reference category? Among men, working such part-time hours is often associated with ill health, something which may increase sedentary behavior.
15. Please would the authors clarify why in this section, the categories for occupation are, superior, intermediary, and others, when their results show four not three categories, and these four categories comprise: superior/intellectual, intermediary profession, employee, others? What do these categories represent? Are employees the only participants who work for other people/other organisations, are the participants in the three remaining categories all self-employed, partners in a firm, etc?

Methods: Statistical analysis

16. Please would the authors clarify what they mean by, “We further studied the relationship between covariables by evaluating the impact to remove the less answered variables on multivariate regression analysis.” Do they mean they omitted some of the covariables due to low response rates? If so, which did they omit – it looks like all those listed in figure 1 were included in the multivariate regressions.
17. As noted above, it would be useful to include the final analytical sample size.

Results: Participants

18. This section only reports on the socio-demographics of the participants. Perhaps the title of this section should reflect this or perhaps the authors could use this section to also highlight some of the interesting findings relating to the occupation and lifestyle characteristics show in Table 1?

Results: Sedentary behaviour and telework

19. The readability of this section could be improved by drawing out the most interesting or important findings from Table 1 rather than repeating the statistics in full here.
20. It would be useful if the authors included a note about the prevalence of telework and each of the outcome categories. It’s noteworthy that almost 62% of the participants did not telework, and less than 8% spent more than 50% of their work time teleworking. It’s also noteworthy that of the participants with data on telework, more than 85% spent less than eight hours per day sitting.
21. Please would the authors clarify what they mean by the phrase, “…those involved in sitting time…”?
22. Please would the authors label each axis in Figure 1?

Results: Mean sitting time depending on sociodemographic, occupational and lifestyle factors; and Prevalence of high sitting time depending on sociodemographic, occupational and lifestyle factors.

23. There is some ambiguity in Figure 2, for example there can not be two categories for age where one is less than or equal to 42 and the other is more than or equal to 42. In which category would a 42 year old be placed?
24. Also, as is usual for a polar graph, the one representing sedentary behaviour as a categorical variable has a starting value of 0 at its centre, whereas the other does not. The chart using categorical data is also much clearer to interpret with its logical radial axis from 0 to 100%; it’s not clear why in the second chart the authors have chosen to use axes of 4hr/day, 5hr15/day, 6hr30/day, 7hr45/day, and 9hr/day.

Results: Sensitivity analyses

25. The authors report “with most variables having a VIF<2. No variable had a VIF>10. Except BMI, all other variables had a VIF<4.” This could phrased a little clearer.
26. The authors state, “We also performed sensitivity analysis by removing variables one by one, besides telework, all variables were similarly answered.” What is meant by “similarly answered”? Also, what were the findings for deleting variables?
27. The authors state, “Sensitivity analyses did not increase over 10% the number of observations used in the multivariate analysis, and results were similar.” What is meant by this?
28. In the analysis in Appendix 2, it looks like the authors conducted three separate logistic regressions – one for each outcome category. If this is so, why didn’t they use a regression analysis for categorical outcomes?

Discussion

29. The authors state, “… other sociodemographic, occupational, and lifestyle variables were weakly associated with sedentary behavior.” Whilst telework showed the strongest association with sedentary behaviour, weren’t the following moderately to strongly associated with sedentary behaviour: education to masters or doctorate level, long working hours, not working at nighttimes, not working at weekends, and high use of social media?

Telework as the main risk factor for sedentary behaviour

30. To improve readability, please would the authors consider reviewing this section for repetition and to improve the grammar? For example, could the following be rewritten, “Our study is novel by enabling to demonstrate that telework is the main risk factor for high sedentary behavior among all variables.” Could this be rewritten?

“Additionally, there was a strong interest for sitting time in teleworkers during the Covid-19 pandemic.” What is meant by this//who has shown interest?

31. The authors state, “In our study, prevalence of workers sitting over 14 hours per day reached 3.2%, 5.4%, and even 10.7% in workers who teleworked more than 50%, 75%, or 100%, respectively. Nonetheless, it is important to note that these represent 4, 2, and 6 participants respectively – in total about 0.4% of the sample. Please would the authors consider putting their finding in to perspective?
32. Regarding the authors’ recommendations for “height‐adjustable desks at home”, the literature on the benefits of such desks is somewhat mixed; and the MET for standing may be lower than 1.5 METS, so the use of standing desks would still be classified as sedentary behaviour according to the authors’ definition of sedentary behaviour. See for example,

Kowalsky RJ, Stoner L, Faghy MA, Barone Gibbs B. A Call to Clarify the Intensity and Classification of Standing Behavior. Int J Environ Res Public Health. 2021 Aug 10;18(16):8460. doi: 10.3390/ijerph18168460. PMID: 34444209; PMCID: PMC8391207.

MacEwen BT, MacDonald DJ, Burr JF. A systematic review of standing and treadmill desks in the workplace. Prev Med. 2015 Jan;70:50-8. doi: 10.1016/j.ypmed.2014.11.011. Epub 2014 Nov 28. PMID: 25448843.

Furthermore, other interventions which take account of environmental, behavioural, and attitudinal factors aimed at tackling sedentary behaviours in workers, may deserve some consideration. See for example,

Coffey A, Parés-Salomón I, Bort-Roig J, Proper KI, Walsh D, Reckman P, Vaqué-Crusellas C, Señé-Mir AM, Puig-Ribera AM, Dowd KP. Factors influencing reducing sedentary time in home office employees. Sci Rep. 2025 Jul 6;15(1):24147. doi: 10.1038/s41598-025-08831-4. PMID: 40619477; PMCID: PMC12230110.

Chu, A. H. Y., Ng, S. H. X., Tan, C. S., Win, A. M., Koh, D., and Müller-Riemenschneider, F. (2016) A systematic review and meta-analysis of workplace intervention strategies to reduce sedentary time in white-collar workers. Obesity Reviews, 17: 467–481. doi: 10.1111/obr.12388.

Influence of sociodemographic on prolonged sedentary behaviour.

33. The authors might need to exercise a little more caution not to overstate their findings. Perhaps they can consider highlighting the key findings and the associations they found consistently among their analyses or discuss why their findings might differ between their analyses? For example, what might explain the following inconsistencies? Having no children was associated with lower odds of sitting 8-14 hours per day, but it was not associated with sedentary behaviour in any of the other analyses; Participants older than 42 years were associated with greater odds of sitting 3-8 hours and 8-14 hours compared with younger participants, but age was not associated with sedentary behaviour in any of the other analyses; The regression results showed no statistically significant associations between region and sedentary behaviour, and whilst the Cohen’s D was weak for Africa, moderate for Asia Pacific and small for America, the Cramer’s V was moderate for Africa, and weak for America.
34. The statement that in some countries men sit more than women contradicts the statement made by the authors in their introduction section.

Occupational variables associated with prolonged sedentary behavior

35. As with the previous section, perhaps the authors could focus on the key results and/or explain the conflicting results in their own study. For example, occupation was not statistically associated with sedentary behaviour in the regression analyses, the category ‘others’ had a moderate effect size with the Cohen’s D analysis but not the Cramer’s V etc. Work-related stress had weak ES in both Cohen’s D and Cramer’s V, and no association with sedentary behaviour in the logistic regressions.
36. Perhaps the authors could consider why they found what they did, for example, consider what type of jobs nightworkers and weekend workers are most likely to do.

Influence of lifestyle characteristics on prolonged sedentary behavior

37. The comments relating to the sections on socio-demographcs and occupational variables, could also be applied to this section. Please would the authors examine the consistency of their findings and what this might mean? Also, please would they consider whether these are risk factors for sedentary behaviours or outcomes of sedentary behaviours?
38. Generally, it would be a more interesting discussion (here and in the previous discussion sections), if the authors drew attention to the key findings and examined them in relation to previous studies in more detail.
39. Also, it is not clear why, in their discussion, the authors have chosen to focus so heavily on the univariate results, especially when the results section focused on descriptive statistics, effect sizes, and multivariate regression analyses; the results section only made a passing reference to the univariate regression analyses in the Appendix; and furthermore, the objective of the study related to the simultaneous inclusion of the covariates.

Limitations

40. As noted earlier, it might be noted if there any potential bias from self-selecting participants?
41. Was there any risk that participants did not understand the concept of teleworking – how clear was(were) the question(s) used to assess teleworking? Could participants be miscategorised?
42. The authors state, “However, we still obtained a large sample size on all variables”. As requested previously, please would the authors state their analytical sample size? What was the pattern of missing data/could the authors have performed multiple imputation etc?
43. Please would the authors be careful not to overstate the positives? For example, 72% of participants were from Europe, and just over 0.2% were from Asia Pacific; and the authors provide no stratification by region (or any other variable), yet the authors state, “Fortunately, our study included participants from many different countries with very diverse backgrounds allowing us to generalize the result in wider populations.”
44. The authors end this section with the statement, “Further studies should observe long term implications of sociodemographic, lifestyle, and occupational variables that can influence the risk of sedentary behavior.” Bearing in mind the authors’ selection of variables was based on studies that had found links between sedentary behaviour and factors relating to sociodemographics, lifestyle, and occupation, please would the authors elaborate on what additional research they think is necessary? Furthermore, do they think there should be any additional research on these factors specifically in relation to teleworking?
45. It is noteworthy that Eurofound’s definition of teleworking begins: “Telework is a work arrangement in which work is performed outside a default place of work, normally the employer’s premises, by means of information and communication technologies (ICT). The main features of telework are the use of computers and telecommunications to change the usual location of work, the frequency with which the worker is working outside the employer’s premises and the number of places where workers work remotely (mobility).”

As the authors have not provided any definition of telework, nor have they provided any information on the actual question(s) participants answered, it is assumed that in their study, telework also means an arrangement whereby work, enabled by ICT, is conducted away from the employer’s premises; and being ICT-led, it will undoubtedly be associated with desk-based work. Therefore, perhaps it would be interesting to contrast the study findings with any studies of sedentary behaviour/sitting time among office workers.

Conclusion

46. The authors state, “Other variables, such as sociodemographic, occupational, and lifestyle variables, were weakly associated with sedentary behavior in univariate regressions, and nearly most did not anymore influence the risk of high sitting time in multivariate regressions, highlighting the major role of telework on sitting time.” This seems an odd conclusion when the authors’ stated objective was: “.. we aimed to investigate the influence of telework on sedentary behavior, taking into account main sociodemographic, occupational and lifestyle factors, in statistical models simultaneously combining all putative variables that may influence sedentary behavior.”.

Abstract

47. It would be helpful if the authors revised their abstract taking account of any changes to the manuscript in light of the feedback.
48. Furthermore, it would be helpful if they would revise confusing statements such as, “We assessed sitting time, and the weight of teleworking concomitantly with other putative influencing variables using effect size, univariate and multivariate regressions.” How is it possible to do this assessment “concomitantly” with a “univariate” regression?
49. Also, please would the authors also correct any inaccuracies, such as, “Nearly all variables influenced the risk of high sitting time in univariate regressions; however, they were not significant except teleworking in multivariate analysis.”?

STROBE Checklist

50. The page numbers given differ to those in the manuscript, possibly due to editorial treatment. Nonetheless, item 12c asks the authors to “Explain how missing data were addressed”, to which the authors responded “Not applicable”. However, it is clear from the manuscript that there are missing data due to item non-response. Please will the authors provide a full response to this question?

Finally, despite the large number of comments above, I think this remains a thought-provoking study, which should be of much interest to employers, occupational health professionals, and policymakers with a public health remit. I wish you all success. With best wishes.
