# Supplementary material for: Teleworking as the main risk factor for sedentary behavior: the COVISTRESS international study
Source: PLoS One. 2026 Sep 10;21(9):e0356764. doi: 10.1371/journal.pone.0356764 (PMC13561329; doi:10.1371/journal.pone.0356764)
Supplement: Supplementary file 11 [file pone.0356764.s013.docx]

*Laboratory of Metabolic Adaptations to Exercise in Physiological and Pathological conditions (****AME2P****)*

**UniversitÉ ClERMONT AUVERGNE** (**UCA**) 63178 Aubière, Clermont Ferrand, France

*Preventive & Occupational Medicine*

– **UniversitY HOSPITAL OF CLERMONT-FERRAND (CHU)**

63000 Clermont-Ferrand

France

*Psychological & Psychosocial Stress*

– National Centre for Scientific Research, **CNRS**, UMR 6024

– **UniversitÉ ClERMONT AUVERGNE** (**UCA**)

63000 Clermont-Ferrand, France

Hijrah Nasir, MSc

*Researcher in Laboratory of Metabolic Adaptations to Exercise in Physiological and Pathological Conditions (AME2P), Université Clermont Auvergne, Clermont-Ferrand, France*

July 2^nd^, 2026

Emily Chenette,

Editor-in-Chief

PLOS One

Dear Reviewer,

Thank you for your time to review our manuscript entitled “Teleworking as the main risk factor for sedentary behavior: The COVISTRESS international study”.

We respond the questions for our article submission point by point as below:

Dear authors

It has been a pleasure to have the opportunity to review the manuscript, “Teleworking as the main risk factor for sedentary behavior: the COVISTRESS’ international study”. As the debate on whether teleworking should or shouldn’t be allowed to continue rumbles on, this study provides an interesting insight into the potential health impacts of teleworking. It is clear that a lot of effort has gone into the study. Nonetheless, as you will see from my notes below, I do have quite a few comments. These are not meant to dishearten. I hope they will lead to improvements in the clarity, readability, and reproducibility of the study, and following this I hope the study will receive a wider audience and further the discussions about teleworking – and how to improve worker health and wellbeing.

[REPLY] Thank you very much for your very helpful and detailed comments. We welcome all the suggestions and we are delighted to clarify all the unclear explanations in our manuscript. Thank you for all the time dedicated to provide us the valuable suggestions for better quality of our manuscript. Please find below point by point responses.

Introduction

1. Please would the authors consider providing a definition of teleworking. In case it’s useful, here is the link to Eurofound’s definition: https://www.eurofound.europa.eu/en/topics/teleworking

[REPLY] Thank you very much for your suggestion and provided link. We have integrated the definition of teleworking in our manuscript. The section Introduction now reads: “The European Foundation defines teleworking as an arrangement in which employees carry out their job tasks away from the usual workplace, typically outside the employer’s premises, using information and communication technologies (REF).”

2. To save the reader having to look up every reference, it would be helpful if the authors were more explicit in their storytelling. For example:

The authors state, “However, a major recent occupational change is related to teleworking, that could also be particularly impacting on sedentary behavior”. If the authors mean the Covid-19 pandemic then please will they say so?

[REPLY] Thank you very much for your suggestion. The section Introduction now reads: […] “A major recent occupational change concerns the rapid expansion of teleworking particularly since the COVID-19 pandemic (REF).” […]

The authors state, “A study quantifying the percentage of telework concluded that the workers with the highest percentage (76%-100% of time spent working) had the highest sedentary time compared with other groups (1%-25%, and 26%-50%).” Please will the authors state who the other groups were?

[REPLY] Thank you very much for your comment. We have added more information regarding the group with different percentage of telework. The section Introduction now reads: Workers with the highest proportion of telework (76%-100% of working time) have been shown to accumulate the highest sedentary time (305 mins/day) compared with other groups (235.2 mins/day for those being 1%-25% in telework, 243.5 mins/day for 26%-50% in telework, and 258.5 mins/day for 51%‐75% in telework) (REF).”

3. To improve clarity, please would the authors explain the following:

What do they mean by “weight” in their statement, “While aforementioned studies … only a few of them concomitantly assessed the weight of telework on sitting time in addition to …”? Whilst at this point I haven’t thoroughly read the manuscript in full, it appears they’ve reported effect size (using Cohen’s D, Cramer’s V, Odds Ratios, etc). Does their study assess weight or effect size?

[REPLY] Thank you very much for your comment. We would like to clarify what we mean with “weight” in this sentence. We wanted to explain that even though other studies have assessed the influence of telework on sitting time, only a few studies run multivariate models taking into account other variables, i.e., sociodemographic, occupational, and lifestyle variables. The sentence on Introduction section now reads: […] “However, most existing studies were conducted on a single country - three in Japan (REF) and one in the Netherland (REF) and only a minority used multivariate models adjusting simultaneously for sociodemographics, occupational, and lifestyle variables (REF). As a result, the independent contribution of telework relative to other determinants of sedentary behavior remains unclear.” […]

Do the authors mean one country, and if so, which country in their statement, “Moreover, those studies focused mainly on one country.”?

[REPLY] Thank you very much for your comment. The section Introduction now reads: […] “However, most existing studies were conducted on a single country - three in Japan (REF) and one in the Netherland (REF).” […]

What “other components” are relevant to the authors’ study in addition to the “many variables”, when they state, “As well as other components (26–28), sedentary behavior can be influenced by many variables (11,12,29)”?

[REPLY] Thank you very much for your comment. We apologize for the typo. The word should be “comportments” or “behavior”. We have rewritten this section. The section Introduction now reads: […] “Numerous sociodemographic, occupational, and lifestyle factors have been associated with sedentary behavior (REF), including older age (REF), women (REF, being single (REF) or living alone (REF), higher education (REF, living in the city vs rural (REF), and lower household income (REF), administrative workers and customer services occupations (REF), work-related stress (REF), smoking (REF), alcohol consumption (REF), cannabis use (REF), obesity (REF), and poor sleep (REF). Each of these factors contributes only partially to sedentary behavior, and their combined influence remains insufficiently explored.” […]

4. The authors state, “However, no studies took into account all those variables simultaneously in the statistical models investigating a link between sedentary time and telework.” Please would the authors explain more fully what the studies have accounted for, and what in their opinion has been missed? Could the lack of simultaneous inclusion of variables in previous studies be due to potential problems relating to the danger of overfitting, multicollinearity, biased causal estimates etc? Could it be due to data availability? Could the previous studies have decided for theoretical and/or statistical reasons not to include some variables?

[REPLY] Thank you very much for your comment. All those studies have accounted for many variables that influence sedentary time, but those studies did not assess the influence of telework. Therefore, we have the proof that numerous variables influence sedentary time … but the few studies on telework assessed just few of those variables. The section Introduction now reads: […] “However, most existing studies were conducted on a single country - three in Japan (REF) and one in the Netherland (REF) and only a minority used multivariate models adjusting simultaneously for sociodemographics, occupational, and lifestyle variables (REF). As a result, the independent contribution of telework relative to other determinants of sedentary behavior remains unclear. Furthermore, previous studies generally categorized sitting time using thresholds such as 8 h/day or, more rarely, 11–12 h/day (REF). To our knowledge, no study has examined extreme sitting time, defined as >14 h/day, largely because available sample sizes were insufficient to capture such rare but clinically relevant behaviors. Identifying this subgroup is important, as extremely prolonged sitting may reflect distinct behavioral patterns and may carry disproportionately high health risks. […]

Methods: Study Design

5. Whilst the authors do state that “Participation was entirely voluntary and anonymous”, it would be helpful if they would also explain if participants are self-selecting and if so, how this might bias the results (and to address this too in the section on limitations).

[REPLY] Thank you very much for your comment. The section Methods Study Design now reads: “The COVISTRESS study is an international, cross-sectional online survey assessing various sociodemographic, occupational, and lifestyle behavior using a self-reported questionnaire. […] Participation was entirely voluntary and anonymous. Participants were self‑selecting.” […]

The Limitations section now reads: “The questionnaire was anonymous (REF) and participants were self‑selecting that may create bias, such as individuals who voluntarily participated may have stronger interest, awareness, or personal experience with the topic, leading to over-representation of certain behaviors. In addition, as this is a fully online questionnaire, it could attract more screen user participants (REF), precluding the population that are not familiar with internet or screen use. Nonetheless, internet use is nowadays widely common (REF).”

Methods: Participants and also Results: Participants

6. The authors state, “We included all participants who answered their sitting time (Figure 1).” Please would the authors double check the data shown in this figure? It shows the number of participants was reduced from 7064 to 6653 by excluding 1333 who did not respond to the question about sitting time. 7064 minus 1333 equals 5,731 not 6653.

[REPLY] Thank you very much for your very detailed findings. The number of participants answered the “COVISTRESS questionnaire” were 7,064. Among them, only 6,653 participants who answered the questions “number of hours of sitting per day”. So, excluded participants were n=411. We apologize for the mistake. We have revised Figure 1 (Flow chart).

7. Figure 1 shows there were 2,904 participants who responded to the question about telework. As the aim of the study was to take into account all the variables (which can influence sedentary behavior) “simultaneously in the statistical models investigating a link between sedentary time and telework” was the final number of participants in the study 2,904 or did the authors do some form of missing data imputation?

[REPLY] Thank you very much for your comment. There were 2,904 participants who responded both to the question about telework and sedentary time. We did not perform multiple imputation because the objective was to investigate associations between sedentary time and telework while simultaneously adjusting for numerous potential confounders. Imputing all explanatory variables would have required additional assumptions regarding the missing-data mechanism and could have introduced additional uncertainty into the multivariable models. We therefore adopted a complete-case approach. In addition, to strengthen the robustness of our findings, we performed several prespecified sensitivity analyses, which all led to consistent conclusions. The last sentence of the “Statistical analyses - Methods section” now reads: […] “We further performed several prespecified sensitivity analyses to strengthen the robustness of our findings. We studied the relationship between covariables to evaluate the impact by removing the less answered variables on multivariate regression analysis, i.e. marital status, parenthood, number of inhabitants, and work addiction (Appendix 5). We conducted step by step regression analyses by adding variables one by one in addition to telework to verify the consistency of the association between sitting time and telework (Appendix 6). A complete-case approach with multivariate model using missing data as a specific class for each variable was also performed (Appendix 7). Furthermore, in order to search for the pattern of missing data, we conducted assessment of non‑response bias through comparison of complete and incomplete respondents (Appendix 8). Statistical significance was marked by p-value ≤0.05. There was no imputation of missing data.”

We added the following sentence in the Results section: “Among the 7064 respondents who answered the COVISTRESS sedentary questionnaire in 2023 and 2024, 6653 answered the sitting time item and were included. Among them, 2904 responded both to sitting time and telework items, and 1417 responded to all questions without any missing data (Figure 1).” Figure 1 has been updated as follow:

We now present a new Appendix 6 (step by step regression analyses) that shows the number of subjects within each model. See below the first lines of the Appendix 6 (please see Appendix 6 for the complete figure):

|  | **Model**  **1**  Sitting time | **Model**  **2**  Model  1  +  age | **Model**  **3**  Model  2  +  sex | **Model**  **4**  Model  3  +  marital status | **Model**  **5**  Model  4  +  parent-hood | **Model**  **6**  Model  5  +  educa-tion level | **Model**  **7**  Model  6  +  region | **Model**  **8**  Model  7  +  number of inha-bitants | **Model**  **9**  Model  8  +  occupation | **Model**  **10**  Model  9  +  working time | **Model**  **11**  Model  10  +  work addiction | **Model**  **12**  Model  11  +  working at night | **Model**  **13**  Model  12  +  working on week-end | **Model**  **14**  Model  13  +  stress at work | **Model**  **15**  Model  14  +  stress at home | **Model**  **16**  Model  15  +  alcohol | **Model**  **17**  Model  16  +  smoking | **Model**  **18**  Model  17  +  cannabis | **Model**  **19**  Model  18  +  sleep quality | **Model**  **20**  Model  19  +  physical activity | **Model**  **21**  Model  20  +  BMI | **Model**  **22**  Model  21  +  time on social media |
| --- | --- | --- | --- | --- | --- | --- | --- | --- | --- | --- | --- | --- | --- | --- | --- | --- | --- | --- | --- | --- | --- | --- |
|  | n=2,904 | n=2,790 | n=2,777 | n=2,606 | n=2,472 | n=2,462 | n=2,379 | n=2,172 | n=2,172 | n=2,161 | n=1,794 | n=1,693 | n=1,675 | n=1,605 | n=1,578 | n=1,570 | n=1,566 | n=1,557 | n=1,557 | n=1,548 | n=1,492 | n=1,417 |
| **Percentage of telework** |  |  |  |  |  |  |  |  |  |  |  |  |  |  |  |  |  |  |  |  |  |  |
| 0% | REF | REF | REF | REF | REF | REF | REF | REF | REF | REF | REF | REF | REF | REF | REF | REF | REF | REF | REF | REF | REF | REF |
| 1-25% | **<0.001** | **<0.001** | **<0.001** | **<0.001** | **<0.001** | **<0.001** | **<0.001** | **<0.001** | **<0.001** | **<0.001** | **<0.001** | **0.001** | **0.002** | **0.003** | **0.003** | **0.008** | **0.007** | **0.008** | **0.009** | **0.004** | **0.012** | **0.014** |
| 26-50% | **<0.001** | **<0.001** | **<0.001** | **<0.001** | **<0.001** | **<0.001** | **<0.001** | **<0.001** | **<0.001** | **<0.001** | **<0.001** | **<0.001** | **<0.001** | **<0.001** | **<0.001** | **<0.001** | **<0.001** | **<0.001** | **<0.001** | **<0.001** | **<0.001** | **<0.001** |
| 51-75% | **<0.001** | **<0.001** | **<0.001** | **<0.001** | **<0.001** | **<0.001** | **<0.001** | **<0.001** | **<0.001** | **<0.001** | **<0.001** | **<0.001** | **<0.001** | **0.001** | **0.001** | **0.001** | **0.001** | **0.001** | **0.001** | **0.001** | **<0.001** | **<0.001** |
| >75% | **<0.001** | **<0.001** | **<0.001** | **<0.001** | **<0.001** | **<0.001** | **<0.001** | **<0.001** | **<0.001** | **<0.001** | **<0.001** | **<0.001** | **<0.001** | **<0.001** | **<0.001** | **<0.001** | **<0.001** | **<0.001** | **<0.001** | **<0.001** | **<0.001** | **<0.001** |

8. Figure 1 also includes the number of responses per variable. What was the pattern of missing data? Was there any imputation? What was the final analytical sample?

[REPLY] Thank you very much for your comment. The “Statistical analyses - Methods section” now reads: […] “Furthermore, in order to search for the pattern of missing data, we conducted assessment of non‑response bias through comparison of complete and incomplete respondents (Appendix 8). […] There was no imputation of missing data.”

The final analytical sample has been added in Figure 1 (see above) as well as in the Results section: “Among the 7064 respondents who answered the COVISTRESS sedentary questionnaire in 2023 and 2024, 6653 answered the sitting time item and were included. Among them, 2904 responded both to sitting time and telework items, and 1417 responded to all questions without any missing data (Figure 1).”

We also now add Appendix 8 (assessment of non‑response bias through comparison of complete and incomplete respondents).

The Result: Sensitivity analysis now reads: […] “ We further studied the relationship between covariables to evaluate the impact by removing the less answered variables on multivariate regression analysis (i.e. marital status, parenthood, number of inhabitants, and work addiction), the number of observations increased by >20% (n=1740) and demonstrated similar results (Appendix 5). We also performed step by step regression analyses by adding variables one by one in addition to telework, demonstrating the consistency of the association between sitting time and telework (Appendix 6). The association remained also consistent by coding all missing data as variables with NA handling to a specific class for each variable. To note, coding missing data as a distinct category produced narrower confidence intervals, indicating greater precision and supporting the stability of the estimated association (Appendix 7). With a cut-off at ≥14 hours, we increase the number of workers sitting more than 14 hours by 50 people (from 108 sitting >14 hours/d to 158 sitting ≥14 hours/d). Using this cutoff as a sensitivity analysis also give similar results for all analyses. Furthermore, the assessment of non‑response bias through comparison of complete and incomplete respondents showed that the respondents with missing data did not differ from those who fully answered all items, except for sleep quality (Cramer’s V=0.173) (Appendix 8).

The limitations section now reads: “Some questions were less answered – especially sensitive questions such as BMI, age, mental health condition, or alcohol/smoking/cannabis uses. However, our sensitivity analysis showed that respondents with missing data did not differ from those who fully answered all items except for variable sleep quality.” […]

9. The authors state that the “teleworking variables were applicable only to this subgroup”. What exactly were the teleworking variables – what were respondents asked?

[REPLY] Thank you very much for your comment and correcting typos / unclear sentences. The sentence now reads: […] “However, analyses focused on employed participants, as teleworking is only applicable for workers.”

Methods: Outcomes Instrument Survey

10. The inclusion of the word “Outcomes” suggest that this will focus only on the outcome – sedentary behavior. However, this section covers more than that. For reader clarity, it might be helpful to replace “Outcomes Instrument Survey” with something along the lines of “Survey variables”; “Main outcomes” with “exposure and outcome variables” or “dependent and independent variables”; and “secondary outcomes” with “covariates”.

[REPLY] Thank you very much for your suggestion. We have changed the title of sub-section to be “Outcomes: Survey variables”, “Main outcomes” with “Outcome and exposure variables”; and “Secondary outcomes” with “Covariates (other influencing variables)”.

11. Please would the authors explain for the readers, why they treated their outcome variable, sitting time per day, as both a quantitative and qualitative variable, and what was their rationale for determining the thresholds for each category? Furthermore, which category are participants assigned to if they sit for 8 hours per day, is it 3-8 or 8-14; or if they sit for 14 hours per day, is it 8-14, or ≥14?

[REPLY] Thank you for this important comment. We agree that the rationale for treating sitting time both as a continuous and a categorical variable should be clarified.

1) Sitting time was analysed both as a continuous variable (hours/day) and as a categorical variable to show the readers the consistency and coherence of the dataset. From personal experience, researchers have a propension to publish the statistical model that give the best significant results. Analysing sitting time both as a continuous and as a categorical variable for us is a sign of trust and transparency. We treated in continuous variable to allow us to check linear associations and we treated as categorical variable in order to identify high‑risk groups as also showed in other studies (Song et al., 2019, Holmes et al., 2026). Continuous modeling allows the detection of linear or non‑linear dose-response associations, which are well‑documented in the literature. Several large cohort studies have shown that each additional hour of sitting is associated with increased cardiometabolic and mortality risk in a graded manner (e.g., Ekelund et al., 2019; Patterson et al., 2018). This approach provides precise effect estimates and facilitates comparison with previous studies reporting continuous associations. We also categorized sitting time to improve interpretability and to identify potential threshold effects. Public‑health guidelines and many epidemiological studies commonly use categorical definitions of sedentary behavior, often distinguishing “low”, “moderate”, and “high” sitting time (typically using thresholds around 6-8 h/day or 10-12 h/day) (Patterson et al., 2018, Bauman et al., 2018; Diaz et al., 2017). These categorical analyses allow readers, clinicians, and policymakers to more easily understand the proportion of individuals exposed to different levels of sedentary behavior and to identify high‑risk groups. Hence, using both continuous and categorical analyses strengthens the robustness of our findings. Continuous models test overall trends, while categorical models identify threshold effects and facilitate interpretation. The convergence of both approaches increases confidence in the validity of our results.
2) Rationale for our specific thresholds (≤3 h, >3-8 h, >8-14 h, >14 h/day) that these thresholds combine conventions used in the literature and the unique characteristics of our dataset. We categorized sitting time into four groups (≤3 h, >3-8 h, >8-14 h, and >14 h/day) to distinguish between low, moderate, high, and extreme levels of sedentary behavior. Sitting more than 8-14 h/day corresponds with thresholds used to define high sitting time in prior research (despite the absence of consensus, most literature usually take a threshold around 6-8 h/day or 10-12 h/day) (Patterson et al., 2018, Bauman et al., 2018; Diaz et al., 2017). While sitting more than 14 h/day demonstrates a novel and unique contribution of our study. Thanks to our large sample size, we were able to identify a small but meaningful subgroup of participants reporting extremely prolonged sitting time (>14 h/day), a level rarely captured in previous studies due to insufficient sample sizes. Extreme sitting time has been hypothesized to carry disproportionately high health risks, but has seldom been examined empirically. Although this group represents a small number of participants, keeping it distinct allows us to explore potential non‑linear or threshold effects that would be obscured if these individuals were merged with those sitting 8-14 h/day.
3) Participants reporting exactly 8 hours/day were assigned to the >3-8 h/day category, and those reporting exactly 14 hours/day were assigned to the >8-14 h/day category.

The Method Section now reads: “Outcome and exposure variables were sedentary behavior and telework. Sedentary behavior was measured by answering the question ‘Number of hours sitting per day’ with a drop-down menu between 0 and 24, with 30 min intervals. Sitting time was used as a quantitative data and as a qualitative data (≤3, >3 to 8, >8 to 14, and >14 hours sitting per day). We treated the outcome using both continuous and categorical analyses to strengthen the robustness of our findings. Continuous models test overall trends, while categorical models identify threshold effects and facilitate interpretation.” […]

We also have updated the Introduction Section. Now the Introduction section read: “Furthermore, previous studies generally categorized sitting time using thresholds such as 8 h/day or, more rarely, 11–12 h/day (REF). To our knowledge, no study has examined extreme sitting time, defined as >14 h/day, largely because available sample sizes were insufficient to capture such rare but clinically relevant behaviors. Identifying this subgroup is important, as extremely prolonged sitting may reflect distinct behavioral patterns and may carry disproportionately high health risks. The COVISTRESS international study provides a unique opportunity to address these gaps. Its large sample size allows (i) the simultaneous inclusion of all major sociodemographic, occupational, and lifestyle variables in multivariate models, and (ii) the identification of extreme sedentary behavior (>14 h/day). For these reasons, sitting time was analysed both as a continuous variable (hours/day) and as a categorical variable (≤3 h/day, >3–8 h/day, >8–14 h/day, and >14 h/day). These categories reflect commonly used thresholds in the literature (≤3 h/day for low sitting, >3–8 h/day for moderate sitting, >8–14 h/day for high sitting) while adding an original category capturing extreme sitting behavior (>14 h/day), made possible by the size and diversity of our dataset.” […]

12. Please would the authors explain why they treated their exposure variable, teleworking, as a categorical variable, and what was their rationale for determining the thresholds for each category? Furthermore, which category are participants assigned to if they telework 25%, 50% and 75% when the potential categories are 1-25%, 25-50%, 50-75%, 75-99%?

[REPLY] Thank you very much for your comment. Telework was measured in the questionnaire using predefined categorical response options (0%, 1-25%, 26-50%, 51-75%, 76-99%, 100%). Because the exposure was originally collected as ordered categories rather than a continuous scale, we analysed telework as a categorical variable. This approach is consistent with prior epidemiological study that used the same percentage‑based telework categories to examine sedentary behavior (Fukushima et al., 2021).

We have updated the manuscript. Now the Methods section reads: “The exposure (telework) was measured by answering the question ‘Percentage of telework’. Telework was originally presented in the questionnaire in ordered categories (0%, 1-25%, 26-50%, 51-75%, 76-99%, 100%).”

If participants telework 25%, the were assigned to group 1-25%, 50% telework in group 26-50%, and telework 75% in group 51-75%. We apologized for the typo. We have corrected the name of each group.

13. Please would the authors explain the rationale for determining the thresholds for the categories of the “secondary” variables? Furthermore, as several of the thresholds overlap, which category are participants assigned to in the following scenarios: 1 million inhabitants; weekly working hours of 40hrs, and 50 hrs; physical activity of 1hr, and 2hrs; BMI of 25; and social media of 20mins, and 1 hour?

[REPLY] We thank the reviewer for this important question. We updated all figures and tables, as well as the text, to clearly state in which category are participants that could be exactly on a cut-off. We added sign <, ≤, ≥, >. All secondary variables were categorized based on the structure of the original questionnaire, established epidemiological standards, or percentile‑based cut‑offs. Several variables (weekly working hours, number of inhabitants, and social media use) were already collected in categorical form and were therefore analysed using collapsed categories to ensure adequate sample size. BMI was categorized using the WHO classification, which is standard in epidemiological research (WHO, 2000). For variables such as physical activity, categories were created using meaningful behavioral thresholds aligned with the distribution of our sample (percentile‑based cut‑offs (P25, P50, P75).

For number of inhabitants, 1 million inhabitants sit in the 2^nd^ category (>5000-1 million).

For weekly working hours, working hours of 40h/week sits on (>30-40h) and 50hr/week sits on (>40-50h).

For total physical activity/week, the questionnaire is from 0 and 24h/week, with 30 min intervals. Physical activity of 1hr sits on >30min-1h/day, while physical activity 2h sits on >1h-2h/day.

For BMI, we took the cut-off often used in the WHO classification with 4 categories: <18.5kg/m² underweight, 18.5-24.99kg/m² normal, ≥25kg/m² overweight, ≥30kg/m² obesity. For BMI of 25 sits on overweight category (≥ 25-30kg/m²). We have updated the categories.

For social media, spent time on social media of 20mins sits on the category ≤20min, and 1 hour sits on >20min-1h.

We have updated our manuscript. The Method section now reads: “**Covariates** (other influencing variables) were sociodemographic, occupational, and lifestyle variables. All secondary variables were categorized based on the structure of the original questionnaire, established epidemiological standards (for BMI and stress level), and percentile-based cut-offs. Sociodemographic were age (≤42 vs >42 years old); sex (men vs women); marital status (in a relationship vs single); parenthood (no child vs ≥1 child); education level (≤high school, undergraduate, master degree, doctorate); number of inhabitants (≤5000, >5000-1 million, >1 million). Occupational variables were occupation (superior, intermediary, employee, others – in detail 1) executives and senior intellectual professionals, e.g., highly skilled professionals, engineers, senior administrators or managers; 2) intermediary professions, e.g., technicians, mid-level supervisors, administrative coordinators; 3) workers or employees, e.g., manual, clerical, or service workers; and 4) others, e.g., farmers, artisans, merchants or entrepreneurs, information and arts professions, self-employed individuals); declared working time per week (≤30h, >30-40h, >40-50h, >50h); working at night or working on weekend; level of stress at work measured using an analog visual scale (low level of stress <50/100, intermediate 50-80, high >80)(55). Lifestyle behavior variables were stress at home (<50, 50-80, >80); alcohol (yes vs no) and tobacco (yes vs no) consumption; physical activity (≤30min, >30mins-1h, >1h-2h, >2h-4h, and >4h/week); body mass index (<18.5kg/m² underweight, 18.5-24.99kg/m² normal, ≥25kg/m² overweight, ≥30kg/m² obesity) (56); and time spent on social media (≤20min, >20min-1h, >1h-3h, >3h/day).”

14. In addition, please would the authors explain the rationale for determining the reference categories in these variables. For example, why select working less than 30 hours per week as the reference category? Among men, working such part-time hours is often associated with ill health, something which may increase sedentary behavior.

[REPLY] We thank the reviewer for this question. In our analyses, reference categories were selected using two simple principles commonly applied in epidemiology: 1) use the lowest or baseline exposure group to make dose-response patterns easy to interpret. This is standard practice for ordered variables such as telework percentage, working hours, physical activity, or sitting time, 2) ensure the reference group is large enough to provide stable estimates. In our dataset, the “≤30 hours/week” group included many participants, making it statistically reliable as a baseline. Using a very small group as the reference would increase variance and reduce precision. Regarding the reviewer’s concern that working <30 hours/week may reflect poorer health in some populations, we note that the reference category is used only as a statistical baseline, not as a health benchmark. Furthermore, our multivariate models adjust for many sociodemographic, occupational, and lifestyle factors, which reduces the risk of confounding. Finally, we verified that using alternative reference categories produced similar results, confirming that our findings were not dependent on the choice of reference group.

Our Method section now reads: […] “declared working time per week (≤30h, >30-40h, >40-50h, >50h)” […]

15. Please would the authors clarify why in this section, the categories for occupation are, superior, intermediary, and others, when their results show four not three categories, and these four categories comprise: superior/intellectual, intermediary profession, employee, others? What do these categories represent? Are employees the only participants who work for other people/other organisations, are the participants in the three remaining categories all self-employed, partners in a firm, etc?

[REPLY] Thank you very much for correcting the typo. For occupation categories, 1) executives and senior intellectual professionals, e.g., highly skilled professionals, engineers, senior administrators or managers; 2) intermediary professions, e.g., technicians, mid-level supervisors, administrative coordinators; 3) workers or employees, e.g., manual, clerical, or service workers; and 4) others, e.g., farmers, artisans, merchants or entrepreneurs, information and arts professions, self-employed individuals. We defined employees if they work as workers in the company/organisations, and we put self-employee in “others”.

We have updated the manuscript. The Methods section now reads: […] “Occupational variables were occupation (superior, intermediary, employee, others) – in detail 1) executives and senior intellectual professionals, e.g., highly skilled professionals, engineers, senior administrators or managers; 2) intermediary professions, e.g., technicians, mid-level supervisors, administrative coordinators; 3) workers or employees, e.g., manual, clerical, or service workers; and 4) others, e.g., farmers, artisans, merchants or entrepreneurs, information and arts professions, self-employed individuals.” […]

Methods: Statistical analysis

16. Please would the authors clarify what they mean by, “We further studied the relationship between covariables by evaluating the impact to remove the less answered variables on multivariate regression analysis.” Do they mean they omitted some of the covariables due to low response rates? If so, which did they omit – it looks like all those listed in figure 1 were included in the multivariate regressions.

[REPLY] Thank you very much for your comment. We performed sensitivity analysis by removing some variables that less answered (i.e. marital status, parenthood, number of inhabitants, and work addiction). Now we added the analysis in Appendix 5.

We have updated the Method – Statistical analysis section. Now it reads: “We further performed several prespecified sensitivity analyses to strengthen the robustness of our findings. We studied the relationship between covariables to evaluate the impact by removing the less answered variables on multivariate regression analysis, i.e. marital status, parenthood, number of inhabitants, and work addiction (Appendix 5). We conducted step by step regression analyses by adding variables one by one in addition to telework to verify the consistency of the association between sitting time and telework (Appendix 6). A complete-case approach with multivariate model using missing data as a specific class for each variable was also performed (Appendix 7). Furthermore, in order to search for the pattern of missing data, we conducted assessment of non‑response bias through comparison of complete and incomplete respondents (Appendix 8). Statistical significance was marked by p-value ≤0.05. There was no imputation of missing data.”

17. As noted above, it would be useful to include the final analytical sample size.

[REPLY] Thank you very much for your comment. We have added this final analytical sample size in Section Result - Participants. Now it reads: “Among the 7064 respondents who answered the COVISTRESS sedentary questionnaire in 2023 and 2024, 6653 answered the sitting time item and were included. Among them, 2904 responded both to sitting time and telework items, and 1417 responded to all questions without any missing data (Figure 1).”

Results: Participants

18. This section only reports on the socio-demographics of the participants. Perhaps the title of this section should reflect this or perhaps the authors could use this section to also highlight some of the interesting findings relating to the occupation and lifestyle characteristics show in Table 1?

[REPLY] Thank you very much for your comment and your suggestion. The title of this section is now “characteristics of participants”. We have added the information regarding occupational and lifestyle characteristics. Now the Section Results: Participants reads: […] “For occupational characteristics, 53.3% of participants was executives and senior intellectual professionals, 21.8% was intermediary professions, and 20.1% was employees. Among participants, 13.1% had a high work addiction, and 26.6% had high level of stress at work. Furthermore, for lifestyle variables, 15.4% had high level of stress at home, 13.4% frequently drank alcohol, 15.1% were smokers, 2.6% used cannabis, 27.6% were overweight and 13.0% obese, and 52.9% used social media >1h/day.”

Results: Sedentary behavior and telework

19. The readability of this section could be improved by drawing out the most interesting or important findings from Table 1 rather than repeating the statistics in full here.

[REPLY] Thank you very much for your comment. We removed some statistics and shortened this section. Please see this section in the answer to your comment number 20, below.

20. It would be useful if the authors included a note about the prevalence of telework and each of the outcome categories. It’s noteworthy that almost 62% of the participants did not telework, and less than 8% spent more than 50% of their work time teleworking. It’s also noteworthy that of the participants with data on telework, more than 85% spent less than eight hours per day sitting.

[REPLY] Thank you very much for your suggestion. We have added this information on Result. Now Results - Sedentary behavior and telework read: “The mean sitting time was 5.7±3.4h/day, with 15.2% of respondents sitting for >8-14h/day and 1.6% sitting >14 hours/day. 61.7% of respondents did telework and 7.5% worked more than 50% in telework. It’s important to note that among the respondents who did telework, 85.9% spent <8h/day sitting. There was a dose response relation between telework and sitting time: workers who teleworked 0%, 1-25%, 26-50%, 51-75%, 76-99%, and 100% respectively sat on average 4.9±3.2h/day, 5.8±3.2h/day, 6.4±3.1h/day, 6.5±3.8h/day, 7.6±3.5h/day, and 9.1±4.3h/day, with a gradual increase in the effect sizes (from a Cohen’s d of 0.28 for 1-25% telework to 1.27 for 100% telework, in comparison with 0% telework). Similarly, for workers who teleworked 0%, 1-25%, 26-50%, 51-75%, 76-99%, and 100%, prevalence of workers sitting 8 to 14h/d increased from 9.1%, 12.7%, 22.2%, 21%, 32.4% to 32.2% respectively, as well as for prevalence of workers sitting more than 14h/d that increased from 1.5%, 1.9%, 0.4%, 3.2%, 5.4% to 10.7%, with also a gradual increase in the effect size (from a Cramer’s V of 0.14 for 1-25% telework to 0.19 for 100% telework, in comparison with 0% telework) (Table 1 and Figure 2).

21. Please would the authors clarify what they mean by the phrase, “…those involved in sitting time…”?

[REPLY] Thank you very much for your comment. We apologize for the sentence that was not clear enough. We have paraphrased the sentence and now the Section Results - Sedentary behavior and telework read: […] “There was a dose response relation between telework and sitting time: workers who teleworked 0%, 1-25%, 26-50%, 51-75%, 76-99%, and 100% respectively sat on average 4.9±3.2h/day, 5.8±3.2h/day, 6.4±3.1h/day, 6.5±3.8h/day, 7.6±3.5h/day, and 9.1±4.3h/day, with a gradual increase in the effect sizes (from a Cohen’s d of 0.28 for 1-25% telework to 1.27 for 100% telework, in comparison with 0% telework) (Table 1 and Figure 2).”

22. Please would the authors label each axis in Figure 1?

[REPLY] Thank you very much for your helpful suggestion. We have updated Figure 2 by adding label in each axis.

Results: Mean sitting time depending on sociodemographic, occupational and lifestyle factors; and Prevalence of high sitting time depending on sociodemographic, occupational and lifestyle factors.

23. There is some ambiguity in Figure 2, for example there cannot be two categories for age where one is less than or equal to 42 and the other is more than or equal to 42. In which category would a 42-year-old be placed?

[REPLY] Thank you very much for your comment. In Figure 2, we did not include age categories. If you mean Figure 3, we categorized age in two categories with median as cut-off. We put participants aged 42-year-old in 1^st^ category (≤42). We have modified Figure 3 to make all the signs clearer. Thank you for pointing the typo. We updated all figures and tables related to age groups that now reads: ≤42 and >42 years old.

24. Also, as is usual for a polar graph, the one representing sedentary behavior as a categorical variable has a starting value of 0 at its centre, whereas the other does not. The chart using categorical data is also much clearer to interpret with its logical radial axis from 0 to 100%; it’s not clear why in the second chart the authors have chosen to use axes of 4hr/day, 5hr15/day, 6hr30/day, 7hr45/day, and 9hr/day.

[REPLY] Thank you very much for your comment. We have a color code in all figures and table. From figure 1 to figure 5 including in all the appendix, and also in table 1: grey is for sitting time as a continuous variable, and nuance of green and red are for sitting time as a categorical variable. We confirmed that the values for the axis are 4hr/day, 5hr15/day, 6hr30/day, 7hr45/day, and 9hr/day because this figure represents sitting time as a continuous variable, in number of hours sitting per day. Please see other articles indexed in PubMed with also a polar plot with an outcome as a continuous variable (Couarraze et al., 2024, Dutheil et al., 2025).

See references below:

Couarraze S, Decormeille G, Delamarre L, Marhar F, Gbaglo K, Avilès Dorlhiac R, Berthon M, Su-I Liu A, Antunes S, Pereira B, Baker JS, Charkhabi M, Ugbolue UC, Bagheri R, Gil-Cosano JJ, Zak M; COVISTRESS Network; Clinchamps M, Dutheil F. Impact of Teleworking on Work-Related and Home-Related Stress at During the First Global Lockdown-The International COVISTRESS Study. Brain Behav. 2025 Jun;15(6):e70592. doi: 10.1002/brb3.70592. PMID: 40444578; PMCID: PMC12123451.

Dutheil F, Saint-Arroman C, Clinchamps M, Flaudias V, Fantini ML, Pereira B, Berthon M, Laporte C, Baker JS, Charkhabi M, Cocco P, Lecca R, Puligheddu M, Figorilli M, Zak M, Ugbolue UC, Ubago-Guisado E, Gracia-Marco L, Bouillon-Minois JB, Vialatte L. Influence of Socio-Demographic, Occupational and Lifestyle Variables on Sleep Time. Nat Sci Sleep. 2025 Jan 30;17:195-210. doi: 10.2147/NSS.S495455. PMID: 39963101; PMCID: PMC11832214.

Results: Sensitivity analyses

25. The authors report “with most variables having a VIF<2. No variable had a VIF>10. Except BMI, all other variables had a VIF<4.” This could be phrased a little clearer.

[REPLY] Thank you very much for your comment. We have simplified our sentence. Section Results: Sensitivity analyses now read: “…. The mean VIF (variance inflation factor) was 2.01 i.e. an acceptable correlation within the predictor variables. All variables had a VIF<4, except BMI.”

26. The authors state, “We also performed sensitivity analysis by removing variables one by one, besides telework, all variables were similarly answered.” What is meant by “similarly answered”? Also, what were the findings for deleting variables?

[REPLY] Thank you very much for your comment. We paraphrased our sentence to make it clearer as well as added New Appendix (Appendix 5).

Please the answer for question number 27 below for the complete paragraph of the Result Section - Sensitivity Analysis.

27. The authors state, “Sensitivity analyses did not increase over 10% the number of observations used in the multivariate analysis, and results were similar.” What is meant by this?

[REPLY] Thank you very much for your comment. We have paraphrased our paragraph and corrected the numbers.

The Result Section now reads: “We further studied the relationship between covariables to evaluate the impact by removing the less answered variables on multivariate regression analysis (i.e. marital status, parenthood, number of inhabitants, and work addiction), the number of observations increased by >20% (n=1740) and demonstrated similar results (Appendix 5). We also performed step by step regression analyses by adding variables one by one in addition to telework, demonstrating the consistency of the association between sitting time and telework (Appendix 6). The association remained also consistent by coding all missing data as a specific class for each variable. To note, coding missing data as a distinct category produced narrower confidence intervals, indicating greater precision and supporting the stability of the estimated association (Appendix 7). With a cut-off at ≥14 hours, we increase the number of workers sitting more than 14 hours by 50 people (from 108 sitting >14 hours/d to 158 sitting ≥14 hours/d). Using this cutoff as a sensitivity analysis also give similar results for all analyses. Furthermore, the assessment of non‑response bias through comparison of complete and incomplete respondents showed that the respondents with missing data did not differ from those who fully answered all items, except for sleep quality (Cramer’s V=0.173) (Appendix 8).

28. In the analysis in Appendix 2, it looks like the authors conducted three separate logistic regressions – one for each outcome category. If this is so, why didn’t they use a regression analysis for categorical outcomes?

[REPLY] Thank you very much for your comment. We used logistic regression analysis using both univariate and multivariate. Appendix 2 is the forest plot with details of factors influencing the risk of sitting >3 to 8 hours, >8 to 14 hours, or >14 hours per day using multivariate logistic regression. While Appendix 3 is forest plot of summary and details of factors influencing the risk of sitting >3 to 8 hours, >8 to 14 hours, or >14 hours per day using univariate logistic regression.

Discussion

29. The authors state, “… other sociodemographic, occupational, and lifestyle variables were weakly associated with sedentary behavior.” Whilst telework showed the strongest association with sedentary behavior, weren’t the following moderately to strongly associated with sedentary behavior: education to masters or doctorate level, long working hours, not working at night time, not working at weekends, and high use of social media.

[REPLY] Thank you very much for your comment. We have modified the sentence to better reflect our results. Now Discussion read: “The main findings were that telework is the main risk factor for high sedentary behavior. The more you telework, the more you sat – with a strong dose response relationship. Higher education, longer working hours, and spent longer time on social media were associated with an increased sitting time, while working at night, and physical activity regardless the durations decreased sitting time. However, all these variables had weaker association compared to telework.

Telework as the main risk factor for sedentary behavior

30. To improve readability, please would the authors consider reviewing this section for repetition and to improve the grammar? For example, could the following be rewritten, “Our study is novel by enabling to demonstrate that telework is the main risk factor for high sedentary behavior among all variables.” Could this be rewritten?

[REPLY] Thank you very much for your comment. We have updated the manuscript to paraphrase it. Now Discussion - Telework as the main risk factor for sedentary behavior read: […] “This study is the first to show that telework was the primary driver of high sedentary behavior when all variables were analyzed together.” […]

“Additionally, there was a strong interest for sitting time in teleworkers during the Covid-19 pandemic.” What is meant by this//who has shown interest?

[REPLY] Thank you very much for your comment. We have rewritten the sentence. Now Section Discussion - Telework as the main risk factor for sedentary behavior read: “…. The increase of telework, especially during the COVID-19 pandemic, has dramatically increased attention to the research on the link between telework and sedentary behavior (REF).”

31. The authors state, “In our study, prevalence of workers sitting over 14 hours per day reached 3.2%, 5.4%, and even 10.7% in workers who teleworked more than 50%, 75%, or 100%, respectively.” Nonetheless, it is important to note that these represent 4, 2, and 6 participants respectively – in total about 0.4% of the sample. Please would the authors consider putting their finding in to perspective?

[REPLY] Thank you very much for your comment. As you noted, few people sit >14 hours per day. We added a new sensitivity analysis and emphasize those small numbers in the Results section as well as in the Discussion. The Results – Sensitivity analyses section now reads: “With a cut-off at ≥14 hours, we increase the number of workers sitting more than 14 hours by 50 people (from 108 sitting >14 hours/d to 158 sitting ≥14 hours/d). Using this cutoff as sensitivity analyses give similar results for all analyses.”

The Discussion now reads: […] “In our study, prevalence of workers sitting over 14 hours per day rose to 3.2%, 5.4%, and 10.7% among those teleworking over 50%, 75%, or 100%, respectively. However, this group represents only 0.4% of all respondents, which limit the generalizability of this finding.” […]

32. Regarding the authors’ recommendations for “height‐adjustable desks at home”, the literature on the benefits of such desks is somewhat mixed; and the MET for standing may be lower than 1.5 METS, so the use of standing desks would still be classified as sedentary behavior according to the authors’ definition of sedentary behavior. See for example,

Kowalsky RJ, Stoner L, Faghy MA, Barone Gibbs B. A Call to Clarify the Intensity and Classification of Standing Behavior. Int J Environ Res Public Health. 2021 Aug 10;18(16):8460. doi: 10.3390/ijerph18168460. PMID: 34444209; PMCID: PMC8391207.

MacEwen BT, MacDonald DJ, Burr JF. A systematic review of standing and treadmill desks in the workplace. Prev Med. 2015 Jan;70:50-8. doi: 10.1016/j.ypmed.2014.11.011. Epub 2014 Nov 28. PMID: 25448843.

Furthermore, other interventions which take account of environmental, behavioral, and attitudinal factors aimed at tackling sedentary behaviors in workers, may deserve some consideration. See for example,

Coffey A, Parés-Salomón I, Bort-Roig J, Proper KI, Walsh D, Reckman P, Vaqué-Crusellas C, Señé-Mir AM, Puig-Ribera AM, Dowd KP. Factors influencing reducing sedentary time in home office employees. Sci Rep. 2025 Jul 6;15(1):24147. doi: 10.1038/s41598-025-08831-4. PMID: 40619477; PMCID: PMC12230110.

Chu, A. H. Y., Ng, S. H. X., Tan, C. S., Win, A. M., Koh, D., and Müller-Riemenschneider, F. (2016) A systematic review and meta-analysis of workplace intervention strategies to reduce sedentary time in white-collar workers. Obesity Reviews, 17: 467–481. doi: 10.1111/obr.12388.

[REPLY] Thank you very much for your comment. Now Section Discussion - Telework as the main risk factor for sedentary behavior read: […] “Accordingly, interventions to reduce sedentary behavior among teleworkers include rehabilitation programs (REF), height-adjustable desks at home (REF) or portable pedal machines, which may offer health benefits (REF). Nonetheless, the effectiveness of height‑adjustable desks remains debated, as standing often does not exceed sedentary‑level energy expenditure (REF). Beyond device‑based approaches, broader strategies addressing environmental, educational/behavioral, and attitudinal determinants may also be important (REF) and interventions integrating multi-component and environmental strategy are most effective in reducing sedentary time (REF).”

Influence of sociodemographic on prolonged sedentary behavior.

33. The authors might need to exercise a little more caution not to overstate their findings. Perhaps they can consider highlighting the key findings and the associations they found consistently among their analyses or discuss why their findings might differ between their analyses? For example, what might explain the following inconsistencies? Having no children was associated with lower odds of sitting 8-14 hours per day, but it was not associated with sedentary behavior in any of the other analyses; Participants older than 42 years were associated with greater odds of sitting 3-8 hours and 8-14 hours compared with younger participants, but age was not associated with sedentary behavior in any of the other analyses; The regression results showed no statistically significant associations between region and sedentary behavior, and whilst the Cohen’s D was weak for Africa, moderate for Asia Pacific and small for America, the Cramer’s V was moderate for Africa, and weak for America.

[REPLY] Thank you very much for your comment. The statement that in some countries men sit more than women contradicts the statement made by the authors in their introduction section. We have corrected this sentence and updated our manuscript.

The Section Discussion “Influence of sociodemographic on prolonged sedentary behavior” now reads: “Many studies have reported the positive link between older age and higher sitting time (REF), although some studies reported the opposite (REF). Higher sitting time at older age is often attributed to social and environmental factors, chronic diseases, depression, and mobility issues (REF). In our study, age showed associations in univariate analyses, but it was not independently related to sedentary behavior in the multivariate models, suggesting that the crude association was explained by other factors such as occupation or telework. We did not find relationships between sex and sitting time, consistent with most studies summarized in two systematic reviews (REF). Although many studies found that women sit more than men (REF), some research from Latin countries (REF) and high-income European countries (REF) have reported the opposite. These inconsistencies in the literature likely reflect cultural, occupational, and lifestyle differences across populations. Prevalence of sitting time in our study was also reported higher in Europe (19.2%) compared to Africa (11.2%). This aligns with global evidence showing that high income countries tend to have nearly double the sedentary time of low income countries (REF), possibly due to the higher proportion of workers in sedentary occupations (REF). However, region was not associated with sedentary behavior in our multivariate models. As in the previous studies (44,79), we observed that lower education was associated with lower sitting time. This is consistent with the fact that individuals with higher education are more likely to work in tertiary and administrative occupations that involve prolonged sitting (REF). Even during leisure time, if people with lower education engage more in television watching, those with higher education also engage in associated leisure sedentary behavior, although different – such as reading, leisure computer use, or passive travel (REF). The fact that people living in megalopolis were more sedentary than people living in smaller city or rural areas has already been reported (REF), and our descriptive results showed similar patterns. However, region and urbanicity did not remain significant in our multivariate models. Regarding marital status, even though we did not find a relationship with sedentary behavior, a systematic review showed that living in couple increased sitting time (REF). Similarly, while some studies have shown that having children reduces prolonged sitting (REF), parenthood was not associated with sedentary behavior in our multivariate analyses, indicating that the crude association was confounded by other variables. Taken together, sociodemographic variables showed limited independent effects once adjusted for all covariates, with only education remaining significant. This highlights that telework is the primary factor associated with prolonged sedentary behavior.”

Occupational variables associated with prolonged sedentary behavior

34. As with the previous section, perhaps the authors could focus on the key results and/or explain the conflicting results in their own study. For example, occupation was not statistically associated with sedentary behavior in the regression analyses, the category ‘others’ had a moderate effect size with the Cohen’s D analysis but not the Cramer’s V etc. Work-related stress had weak ES in both Cohen’s D and Cramer’s V, and no association with sedentary behavior in the logistic regressions.

[REPLY] Thank you very much for your comment. Focusing on the multivariate findings, we observed that although several occupational variables showed small or moderate associations in descriptive or univariate analyses, only working hours and working at night remained significant in the multivariate linear regression, and only working at night appeared to be consistently significant in all categories in the multivariate logistic models. Inconsistencies in descriptive metrics (Cohen’s D, Cramer’s V) also come from the absence of adjustment for confounding. For example, the “others” occupation group showed a moderate Cohen’s D but no association after adjustment, suggesting that crude differences were driven by other factors such as telework. Work‑related stress showed similarly weak and non‑independent effects. Overall, the adjusted results indicate that occupational variables have only a limited independent role, while telework remains the strongest and most consistent occupational determinant of sedentary behaviour.

Now Section Discussion - Occupational variables associated with prolonged sedentary behavior read: “Occupational characteristics other than telework also influenced prolonged sedentary behavior (REF). Office-based workers are likely to be more sedentary (REF), spending two-thirds to three quarters of their working hours sitting (REF) and have fewer breaks as well as fewer bouts of physical activity during working time (REF). They are also more sedentary outside work (REF). Studies reported high levels of sedentary behavior in administrative workers as well as in customer service (REF). However, our multivariate model did not confirm an independent association between sedentary behavior and occupation. Sedentary behavior has also been linked to work-related stress. Although our adjusted analysis did not show an association, previous studies have reported that higher levels of occupational sedentary behavior were significantly associated with mental health problems (REF), such as stress (REF), sadness and anxiety (REF), and psychological distress (REF) - possibly related to psychosocial risk factors (REF) and lower work engagement (REF). We included work addiction in our analysis. To our knowledge, this association was never explored in the literature. Although this association was not confirmed in multivariate model, the hypothesis remains plausible: work addiction may increase screen-time and reduce opportunities for movement (REF). In our multivariate analysis, working at night was a protective factor for extreme sitting time, consistent with a meta-analysis showing that people working at night were less sedentary than daytime workers (REF). A possible explanation is that night workers are commonly employed in service and information sectors (REF) that require continuous operations, such as healthcare, emergency services, manufacturing, transportation, hospitality, and retail. We also included working on weekends as an occupational variable that tend to be associated with lower sitting time in our multivariate model. While there are few studies on the negative effects of weekend work on sleep disturbance or social interaction (REF), we did not find any literature exploring its association with sedentary behavior.”

35. Perhaps the authors could consider why they found what they did, for example, consider what type of jobs nightworkers and weekend workers are most likely to do.

[REPLY] Thank you very much for your helpful comment. We have added this information. Now Section Discussion Occupational variables associated with prolonged sedentary behavior read: […]“A possible explanation is that night workers are commonly employed in service and information sectors (REF) that require continuous operations, such as healthcare, emergency services, manufacturing, transportation, hospitality, and retail.” […]

Influence of lifestyle characteristics on prolonged sedentary behavior

36. The comments relating to the sections on socio-demographics and occupational variables, could also be applied to this section. Please would the authors examine the consistency of their findings and what this might mean? Also, please would they consider whether these are risk factors for sedentary behaviors or outcomes of sedentary behaviors?

[REPLY] Thank you very much for your comment.

Now Section Discussion - Influence of lifestyle characteristics on prolonged sedentary behavior reads: “Lifestyle factors also showed associations with sedentary behavior. In our study, we included stress at home in our analysis. Even if the direct link between sitting time and stress at home has not been widely explored and no association was found in our multivariate analysis, its association with mental health was often discussed in the literature, especially its relation with greater stress, mood disturbance (REF), depression and anxiety (REF), as well as mental disorders (REF). However the link between stress and sedentary behavior can be bidirectional where stress possibly as risk factor or a consequence of prolonged sitting (REF). Furthermore, we reported a negative association between good sleep quality and high sitting time, consistent with a meta-analysis showing that high sedentary people were more likely to have an increased risk of insomnia and sleep disturbance (REF). Again, this relationship maybe bidirectional, poor sleep may reduce energy and motivation for movement, while prolonged sitting increases linked to poor sleep quality (REF). Even though our multivariate analysis did not find any association between BMI and sedentary, previous studies reported that abnormal BMI (<18.5 or >25) were more sedentary (REF). It demonstrated behavioral patterns such as increased TV watching among individuals with higher BMI (REF), and gender specific difference in screen time among underweight individuals (REF). This association could be bidirectional with some studies reported the increase risk of obesity due to sedentary (REF). Even if our study did not find any link between smoking, alcohol and cannabis consumption with sedentary behavior, several studies have assessed these relations (REF). For example, older people who ever smoked were more likely to be sedentary (REF), and alcohol and cannabis use were positively associated with sedentary behavior in both adolescents (REF) and adults (REF). We confirmed the expected association between higher physical activity and lower sedentary behavior (REF), as well as the relationship between sedentary and time spent on social media consistent with studies linking high sedentary time to greater cellphone (REF) and screen use (REF). Overall, while several lifestyle factors showed association with sitting time, many of these relationships weakened or disappeared in the multivariate models, suggesting that they may be partly explained by other correlated factors. Importantly, telework remained the strongest and most consistent determinants of sedentary behavior.”

37. Generally, it would be a more interesting discussion (here and in the previous discussion sections), if the authors drew attention to the key findings and examined them in relation to previous studies in more detail.

[REPLY] Thank you very much for your comment. Around 50% of the discussion has been changed to better emphasize the key findings and examined them in relation to previous studies in more detail. We also greatly improved the introduction in the same way, to better show the readers the novelty of our study.

38. Also, it is not clear why, in their discussion, the authors have chosen to focus so heavily on the univariate results, especially when the results section focused on descriptive statistics, effect sizes, and multivariate regression analyses; the results section only made a passing reference to the univariate regression analyses in the Appendix; and furthermore, the objective of the study related to the simultaneous inclusion of the covariates.

[REPLY] Thank you very much for your very helpful comment. We have highlighted the result of our multivariate analysis. This is a benefit from our article, because most literature focused on few variables and did not perform multivariate analyses simultaneously. We have emphasized this in our Introduction as well as Discussion Section.

Now the Introduction section reads: “Prolonged sedentary behavior is associated with increased risks of all-cause mortality, cardiometabolic disease, cancer, and mental health disorders, independently of physical activity levels (REF). Sedentary behavior is defined as any waking behavior characterized by an energy expenditure ≤1.5 metabolic equivalents, while in a sitting, reclining, or lying posture (REF). Despite strong evidence of its detrimental effects, sitting time remains high worldwide (REF). Numerous sociodemographic, occupational, and lifestyle factors have been associated with sedentary behavior (REF), including older age (REF), women (REF), being single (REF) or living alone (REF), higher education (REF), living in the city vs rural (REF), and lower household income (REF), administrative workers and customer services occupations (REF), work-related stress (REF), smoking (REF), alcohol consumption (REF), cannabis use (REF), obesity (REF), and poor sleep (REF).  Each of these factors contributes only partially to sedentary behavior, and their combined influence remains insufficiently explored.

A major recent occupational change concerns the rapid expansion of teleworking particularly since the COVID-19 pandemic. The European Foundation defines teleworking as an arrangement in which employees carry out their job tasks away from the usual workplace, typically outside the employer’s premises, using information and communication technologies (REF). Several studies and systematic reviews have reported substantial increases in sedentary time among teleworkers (REF). A systematic review showed between +6% and +67% increase in sedentary behavior during the COVID-19 pandemic (REF), and thereafter (REF), as well as shorter daily breaks during typical working hours (REF). Workers with the highest proportion of telework (76%-100% of working time) have been shown to accumulate the highest sedentary time (305 mins/day) compared with other groups (235.2 mins/day for those being 1%-25% in telework, 243.5 mins/day for 26%-50% in telework, and 258.5 mins/day for 51%‐75% in telework) (REF). However, most existing studies were conducted on a single country - three in Japan (REF) and one in the Netherland (REF) and only a minority used multivariate models adjusting simultaneously for sociodemographics, occupational, and lifestyle variables (REF). As a result, the independent contribution of telework relative to other determinants of sedentary behavior remains unclear. Furthermore, previous studies generally categorized sitting time using thresholds such as 8 h/day (REF) or, more rarely, 11–12 h/day (REF). To our knowledge, no study has examined extreme sitting time, defined as >14 h/day, largely because available sample sizes were insufficient to capture such rare but clinically relevant behaviors. Identifying this subgroup is important, as extremely prolonged sitting may reflect distinct behavioral patterns and may carry disproportionately high health risks.

The COVISTRESS international study provides a unique opportunity to address this gap. Its sample size allows the simultaneous inclusion of all major sociodemographics, occupational, and lifestyle variables and the identification of extreme sedentary behavior (>14 h/day). For these reasons, sitting time was analyzed both as a continuous variable (hours/day) and as a categorical variable (≤3 h/day, >3–8 h/day, >8–14 h/day, and >14 h/day). These categories reflect commonly used thresholds in the literature (≤3 h/day for low sitting, >3–8 h/day for moderate sitting, >8–14 h/day for high sitting) while adding an original category capturing extreme sitting behavior (>14 h/day), made possible by the size and diversity of our dataset.

Therefore, we aimed to investigate the influence of telework on sedentary behavior, taking into account major sociodemographic, occupational and lifestyle factors, using statistical models that simultaneously combining all putative variables that may influence sedentary behavior.”

For the complete revision of our manuscript in Discussion Section, please see the answers for questions number 33, 34, and 36.

Limitations

39. As noted earlier, it might be noted if there any potential bias from self-selecting participants?

[REPLY] Thank you very much for your comment. Now Section Limitations read: […] “The questionnaire was anonymous (REF) and participants were self‑selecting that may create bias, such as individuals who voluntarily participated may have stronger interest, awareness, or personal experience with the topic, leading to overrepresentation of certain behaviors. In addition, as this is a fully online questionnaire, it could attract more screen users’ participants (113), precluding the population that are not familiar with internet or screen use. Nonetheless, internet use is nowadays widely common (REF).” […]

40. Was there any risk that participants did not understand the concept of teleworking – how clear was(were) the question(s) used to assess teleworking? Could participants be mis-categorized?

[REPLY] Thank you very much for your comment. Now Section Limitations read: […] “As the questionnaire on telework only asked “the percentage of telework”, participants could have different interpretation about the concept of teleworking.” […]

41. The authors state, “However, we still obtained a large sample size on all variables”. As requested previously, please would the authors state their analytical sample size? What was the pattern of missing data/could the authors have performed multiple imputation etc?

[REPLY] Thank you very much for your comment. As we previously explained, we did not perform multiple imputation because the objective was to investigate associations between sedentary time and telework while simultaneously adjusting for numerous potential confounders. We performed a complete-case approach. In addition, to strengthen the robustness of our findings, we performed several prespecified sensitivity analyses, which all led to consistent conclusions. We have added more information in the Limitation section and we have added New Appendix (please see Appendix 5, 6, 7, and 8) for the Sensitivity analysis.

The Limitation section now reads: […] “Some questions were less answered – especially sensitive questions such as BMI, age, mental health condition, or alcohol/smoking/cannabis uses. However, our sensitivity analysis showed that respondents with missing data did not differ from those who fully answered all items except for variable sleep quality. Moreover, we still obtained a large sample size on all variables. Some less answered questions might have promoted under or over-estimation on the data collection (125), but we controlled the robustness of our statistical by including sensitivity analysis. Furthermore, all sensitivity analysis confirmed that the association between sitting time and telework remained consistent across different modeling strategies, including when missing data were explicitly coded as a separate category.” […]

42. Please would the authors be careful not to overstate the positives? For example, 72% of participants were from Europe, and just over 0.2% were from Asia Pacific; and the authors provide no stratification by region (or any other variable), yet the authors state, “Fortunately, our study included participants from many different countries with very diverse backgrounds allowing us to generalize the result in wider populations.”

[REPLY] Thank you very much for your helpful comment. We have revised this sentence.

The Limitation section now reads: […] “Fortunately, our study included participants from many different countries with very diverse backgrounds (122). We have respondents for most continents, even from Africa that is commonly less explored in the literature. However, we lack respondents from Asia Pacific, precluding a wide international generalizability of our findings.” […]

43. The authors end this section with the statement, “Further studies should observe long term implications of sociodemographic, lifestyle, and occupational variables that can influence the risk of sedentary behavior.” Bearing in mind the authors’ selection of variables was based on studies that had found links between sedentary behavior and factors relating to sociodemographics, lifestyle, and occupation, please would the authors elaborate on what additional research they think is necessary? Furthermore, do they think there should be any additional research on these factors specifically in relation to teleworking?

[REPLY] Thank you very much for your comment.

The Limitation section now reads: […] “Future research should use longitudinal and experimental designs to clarify whether sociodemographic, lifestyle, and occupational factors are true determinants or consequences of sedentary behavior, and specifically to examine how these relationships evolve in teleworking contexts (e.g., different telework intensities, hybrid models, and organizational policies).”

44. It is noteworthy that Eurofound’s definition of teleworking begins: “Telework is a work arrangement in which work is performed outside a default place of work, normally the employer’s premises, by means of information and communication technologies (ICT). The main features of telework are the use of computers and telecommunications to change the usual location of work, the frequency with which the worker is working outside the employer’s premises and the number of places where workers work remotely (mobility).”

As the authors have not provided any definition of telework, nor have they provided any information on the actual question(s) participants answered, it is assumed that in their study, telework also means an arrangement whereby work, enabled by ICT, is conducted away from the employer’s premises; and being ICT-led, it will undoubtedly be associated with desk-based work. Therefore, perhaps it would be interesting to contrast the study findings with any studies of sedentary behavior/sitting time among office workers.

[REPLY] Thank you very much for your comment. We have to acknowledge that we did not provide any definition of telework in the questionnaire. We discuss this in the Limitation. The Limitation section now reads: […] “As the questionnaire on telework only asked “the percentage of telework”, participants could have different interpretation about the concept of teleworking.” […]

We also added more information with studies of sedentary behavior among office workers to contrast with our findings. The Discussion - Telework as the main risk factor for sedentary behavior now reads: […] “As the main feature of telework is the use of computers and telecommunications, numerous studies have shown that employees engaged in computer based or desk-based work accumulate great prolonged sitting time during the workday (REF, REF). They were more likely had longer sustained sedentary time (bouts >30 minutes) and fewer breaks in sedentary time (53). Evidence also suggests that remote or home-based work environments may further increase sedentary due to fewer interruptions and longer working hours (40,54). Therefore, the prolonged sitting time observed among frequent teleworkers in our study aligns with findings on sedentary behavior in office-based populations using objective devices (53,55), where ICT-enabled work is strongly associated with prolonged sitting.” […]

Conclusion

45. The authors state, “Other variables, such as sociodemographic, occupational, and lifestyle variables, were weakly associated with sedentary behavior in univariate regressions, and nearly most did not anymore influence the risk of high sitting time in multivariate regressions, highlighting the major role of telework on sitting time.” This seems an odd conclusion when the authors’ stated objective was: “.. we aimed to investigate the influence of telework on sedentary behavior, taking into account main sociodemographic, occupational and lifestyle factors, in statistical models simultaneously combining all putative variables that may influence sedentary behavior.”

[REPLY] Thank you very much for your comment.

The Conclusion section now reads: “We demonstrated that teleworking is the main risk factor for high sedentary behavior, with a strong dose response relationship. The more you telework, the greater the sitting time. Overall, while several other sociodemographic, occupational, and lifestyle factors showed association with sitting time in univariate model, many of these relationships weakened or disappeared in the multivariate models, except telework that remained the most consistent determinants of sedentary behavior when concomitantly put together with other variables. Considering the physical and mental health consequences of sedentary behavior, preventive strategies at work should be encouraged to counteract the negative effect of telework on sedentary behavior.”

Abstract

46. It would be helpful if the authors revised their abstract taking account of any changes to the manuscript in light of the feedback.

[REPLY] Thank you very much for your comment. We have rewritten the Abstract.

The Abstract now reads:

**Background**: If teleworking is already associated with prolonged sedentary behavior, the literature lacks an integrating model with all putative influencing variables, as well as a sufficient number of participants to explore extreme sitting behavior. Therefore, we aimed to investigate the influence of teleworking on sitting time, considering main sociodemographic, occupational and lifestyle factors.

**Methods**: The COVISTRESS study is an international observational study, using self-reported questionnaires (REDCap® software). We used linear and logistic multivariate model to assess the impact of telework on sitting time concomitantly with other sociodemographic, occupational, and lifestyle variables.

**Results**: 6,653 respondents were included. The mean sitting time was 5.6±3.4h/day, with 15.2% of respondents sitting for >8-14h/day and 1.6% sitting >14 hours/day. Workers who teleworked 0%, 1-25%, 26-50%, 51-75%, 76-99%, and 100% respectively sat on average 4.9±3.2h/day, 5.8±3.2h/day, 6.4±3.1h/day, 6.5±3.8h/day, 7.6±3.5h/day, and 9.1±4.3h/day, and the prevalence of workers sitting >14h/day increased from 1.5%, 1.9%, 0.4%, 3.2%, 5.4% to 10.7%. Compared with workers who did not telework, the risk of sitting 8-14 h/day was multiplied by 1.9 (1.2 to 3.0) for people working 1-25% in telework, by 4.7 (2.2 to 9.7) for people working 26-50% in telework, by 4.3 (1.5 to 12.2) for people working 51-75% in telework, and by 9.2 (1.6 to 51.5) for people working >75% in telework; the risk of sitting ≥14h/day was multiplied by 5.8 (1.07 to 31.5) for people working 51-75% in telework and dramatically increased by 34.3 (3.71 to 317) for people working >75% in telework in multivariate model.

**Conclusion**: Teleworking is the strongest risk factor for high sitting time, with a marked dose-response relation. While several other sociodemographic, occupational, and lifestyle factors showed association with sitting time in univariate model, many of these relationships weakened or disappeared in the multivariate models, except telework that remained the most consistent determinants of sedentary behavior when concomitantly put together with all other variables.”

47. Furthermore, it would be helpful if they would revise confusing statements such as, “We assessed sitting time, and the weight of teleworking concomitantly with other putative influencing variables using effect size, univariate and multivariate regressions.” How is it possible to do this assessment “concomitantly” with a “univariate” regression?

[REPLY] Thank you very much for your comment and your correction. We have corrected and rewritten the Abstract. The Abstract: Method now reads: “We used linear and logistic multivariate model to assess the impact of telework on sitting time concomitantly with other sociodemographic, occupational, and lifestyle variables.”

48. Also, please would the authors also correct any inaccuracies, such as, “Nearly all variables influenced the risk of high sitting time in univariate regressions; however, they were not significant except teleworking in multivariate analysis.”?

[REPLY] Thank you very much for your comment and your correction. We have rewritten the Abstract: Result. Now it reads: […] “Compared with workers who did not telework, the risk of sitting 8-14 h/day was multiplied by 1.9 (1.2 to 3.0) for people working 1-25% in telework, by 4.7 (2.2 to 9.7) for people working 25-50% in telework, by 4.3 (1.5 to 12.2) for people working 50-75% in telework, and by 9.2 (1.6 to 51.5) for people working >75% in telework; the risk of sitting >14h/day was multiplied by 5.8 (1.07 to 31.5) for people working 50-75% in telework and dramatically increased by 34.3 (3.71 to 317) for people working >75% in telework in multivariate model.”

STROBE Checklist

49. The page numbers given differ to those in the manuscript, possibly due to editorial treatment. Nonetheless, item 12c asks the authors to “Explain how missing data were addressed”, to which the authors responded “Not applicable”. However, it is clear from the manuscript that there are missing data due to item non-response. Please will the authors provide a full response to this question?

[REPLY] Thank you very much for your comment and your correction. We have revised our STROBE Checklist. The explanation about how missing data were addressed can be found on page 9 (Method: Statistical analysis) and page 15 (Result: Sensitivity analysis). We now also present a new Appendix 6 (step by step regression analyses) that shows the number of subjects within each model and Appendix 8 (assessment of non‑response bias among the 2904 participants who responded both to sitting time and telework items (comparisons between the 1417 participants who responded to all questions without any missing data, and the 1487 respondents who did not respond to at least one question).

Finally, despite the large number of comments above, I think this remains a thought-provoking study, which should be of much interest to employers, occupational health professionals, and policymakers with a public health remit. I wish you all success. With best wishes.

[REPLY] We would like to express our sincere gratitude to the reviewer for the exceptional time, care, and expertise invested in evaluating our manuscript. We agree with almost all points the reviewer have raised and we truly appreciate the willingness to examine every aspect of the study so thoroughly, from conceptual framing to methodological clarity and interpretation of results. The reviewer’s insights and suggestions help us to refine our arguments, clarify our analyses, and improve the overall coherence of the paper. We are genuinely grateful for the constructive nature of the feedback that guided us for more robust and meaningful revisions. We are especially encouraged by the reviewer’s concluding remarks acknowledging the relevance and potential impact of our study. We thank the reviewer again for the generosity, professionalism, and commitment to helping us enhance the quality of this manuscript.

Dear Dr. Nasir,

Thank you for submitting your manuscript to PLOS ONE. After careful consideration, we feel that it has merit but does not fully meet PLOS ONE’s publication criteria as it currently stands. Therefore, we invite you to submit a revised version of the manuscript that addresses the points raised during the review process.

==============================

Comments were received from only one reviewer.

Please improve the quality of your manuscript based on the comments.

==============================

Please submit your revised manuscript by Jun 05 2026 11:59PM. If you will need more time than this to complete your revisions, please reply to this message or contact the journal office at plosone@plos.org. When you're ready to submit your revision, log on to https://www.editorialmanager.com/pone/ and select the 'Submissions Needing Revision' folder to locate your manuscript file.

Please include the following items when submitting your revised manuscript:

A letter that responds to each point raised by the academic editor and reviewer(s). You should upload this letter as a separate file labeled 'Response to Reviewers'.

A marked-up copy of your manuscript that highlights changes made to the original version. You should upload this as a separate file labeled 'Revised Manuscript with Track Changes'.

An unmarked version of your revised paper without tracked changes. You should upload this as a separate file labeled 'Manuscript'.

If you would like to make changes to your financial disclosure, please include your updated statement in your cover letter. Guidelines for resubmitting your figure files are available below the reviewer comments at the end of this letter.

If applicable, we recommend that you deposit your laboratory protocols in protocols.io to enhance the reproducibility of your results. Protocols.io assigns your protocol its own identifier (DOI) so that it can be cited independently in the future. For instructions see: https://journals.plos.org/plosone/s/submission-guidelines#loc-laboratory-protocols. Additionally, PLOS ONE offers an option for publishing peer-reviewed Lab Protocol articles, which describe protocols hosted on protocols.io. Read more information on sharing protocols at https://plos.org/protocols?utm_medium=editorial-email&utm_source=authorletters&utm_campaign=protocols.

As the corresponding author, your ORCID iD is verified in the submission system and will appear in the published article. PLOS supports the use of ORCID, and we encourage all coauthors to register for an ORCID iD and use it as well. Please encourage your coauthors to verify their ORCID iD within the submission system before final acceptance, as unverified ORCID iDs will not appear in the published article. Only the individual author can complete the verification step; PLOS staff cannot verify ORCID iDs on behalf of authors.

We look forward to receiving your revised manuscript.

Kind regards,

Jianguo Wang, PhD

Academic Editor

PLOS One

Journal Requirements:

When submitting your revision, we need you to address these additional requirements.

1. Please ensure that your manuscript meets PLOS ONE's style requirements, including those for file naming. The PLOS ONE style templates can be found at

https://journals.plos.org/plosone/s/file?id=wjVg/PLOSOne_formatting_sample_main_body.pdf and

https://journals.plos.org/plosone/s/file?id=ba62/PLOSOne_formatting_sample_title_authors_affiliations.pdf.

2. We note that your Data Availability Statement is currently as follows: [All relevant data are within the manuscript and its Supporting Information files. ]

Please confirm at this time whether or not your submission contains all raw data required to replicate the results of your study. Authors must share the “minimal data set” for their submission. PLOS defines the minimal data set to consist of the data required to replicate all study findings reported in the article, as well as related metadata and methods (https://journals.plos.org/plosone/s/data-availability#loc-minimal-data-set-definition).

For example, authors should submit the following data:

- The values behind the means, standard deviations and other measures reported;

- The values used to build graphs;

- The points extracted from images for analysis.

Authors do not need to submit their entire data set if only a portion of the data was used in the reported study.

If your submission does not contain these data, please either upload them as Supporting Information files or deposit them to a stable, public repository and provide us with the relevant URLs, DOIs, or accession numbers. For a list of recommended repositories, please see https://journals.plos.org/plosone/s/recommended-repositories.

If there are ethical or legal restrictions on sharing a de-identified data set, please explain them in detail (e.g., data contain potentially sensitive information, data are owned by a third-party organization, etc.) and who has imposed them (e.g., an ethics committee). Please also provide contact information for a data access committee, ethics committee, or other institutional body to which data requests may be sent. If data are owned by a third party, please indicate how others may request data access.

[REPLY] Thank you very much for your remarks. We have added the dataset in Supporting Information (excel file).

3. One of the noted authors is a group or consortium [The COVISTRESS network]. In addition to naming the author group, please list the individual authors and affiliations within this group in the acknowledgments section of your manuscript. Please also indicate clearly a lead author for this group along with a contact email address.

[REPLY] Thank you very much for your comment. We have added the consortium information and the list the individual authors and their affiliations in the Acknowledgement.

4. Your ethics statement should only appear in the Methods section of your manuscript. If your ethics statement is written in any section besides the Methods, please delete it from any other section.

[REPLY] Thank you very much for your comment. We have deleted the ethics statement that we previously put under the Availability of data and materials.

5. If the reviewer comments include a recommendation to cite specific previously published works, please review and evaluate these publications to determine whether they are relevant and should be cited. There is no requirement to cite these works unless the editor has indicated otherwise.

[REPLY] Thank you very much for your comment. We have added several citations suggested by reviewers due to their relevancies to our results.

[Note: HTML markup is below. Please do not edit.]

Reviewers' comments:

Reviewer's Responses to Questions

Comments to the Author

1. Is the manuscript technically sound, and do the data support the conclusions?

The manuscript must describe a technically sound piece of scientific research with data that supports the conclusions. Experiments must have been conducted rigorously, with appropriate controls, replication, and sample sizes. The conclusions must be drawn appropriately based on the data presented.

Reviewer #1: Partly

2. Has the statistical analysis been performed appropriately and rigorously?

Reviewer #1: I Don't Know

3. Have the authors made all data underlying the findings in their manuscript fully available?

The PLOS Data policy requires authors to make all data underlying the findings described in their manuscript fully available without restriction, with rare exception (please refer to the Data Availability Statement in the manuscript PDF file). The data should be provided as part of the manuscript or its supporting information, or deposited to a public repository. For example, in addition to summary statistics, the data points behind means, medians and variance measures should be available. If there are restrictions on publicly sharing data—e.g. participant privacy or use of data from a third party—those must be specified.

Reviewer #1: Yes

4. Is the manuscript presented in an intelligible fashion and written in standard English?

PLOS ONE does not copyedit accepted manuscripts, so the language in submitted articles must be clear, correct, and unambiguous. Any typographical or grammatical errors should be corrected at revision, so please note any specific errors here.

Reviewer #1: Yes

5. Review Comments to the Author

Please use the space provided to explain your answers to the questions above. You may also include additional comments for the author, including concerns about dual publication, research ethics, or publication ethics. (Please upload your review as an attachment if it exceeds 20,000 characters)

Reviewer #1: Dear authors

It has been a pleasure to have the opportunity to review the manuscript, “Teleworking as the main risk factor for sedentary behavior: the COVISTRESS’ international study”. As the debate on whether teleworking should or shouldn’t be allowed to continue rumbles on, this study provides an interesting insight into the potential health impacts of teleworking. It is clear that a lot of effort has gone into the study. Nonetheless, as you will see from my notes, I do have quite a few comments. These are not meant to dishearten. I hope they will lead to improvements in the clarity, readability, and reproducibility of the study, and following this I hope the study will receive a wider audience and further the discussions about teleworking – and how to improve worker health and wellbeing.

The remainder of my feedback is in the attachment.

Best wishes

6. PLOS authors have the option to publish the peer review history of their article (what does this mean?). If published, this will include your full peer review and any attached files.

If you choose “no”, your identity will remain anonymous but your review may still be made public.

Do you want your identity to be public for this peer review? For information about this choice, including consent withdrawal, please see our Privacy Policy.

Reviewer #1: No

[NOTE: If reviewer comments were submitted as an attachment file, they will be attached to this email and accessible via the submission site. Please log into your account, locate the manuscript record, and check for the action link "View Attachments". If this link does not appear, there are no attachment files.]

To ensure your figures meet our technical requirements, please review our figure guidelines: https://journals.plos.org/plosone/s/figures

You may also use PLOS’s free figure tool, NAAS, to help you prepare publication quality figures: https://journals.plos.org/plosone/s/figures#loc-tools-for-figure-preparation.

NAAS will assess whether your figures meet our technical requirements by comparing each figure against our figure specifications.

We hope our work will be considered favorably and look forward to hearing from you.

Sincerely yours,

Hijrah Nasir
