# Supplementary material for: Teleworking as the main risk factor for sedentary behavior: the COVISTRESS international study
Source: PLoS One. 2026 Sep 10;21(9):e0356764. doi: 10.1371/journal.pone.0356764 (PMC13561329; doi:10.1371/journal.pone.0356764)
Supplement: Peer review history

# Peer review: Decision Letter 0

**DOI:** 10.1371/journal.pone.0356764.r001

**Authors:** Jianguo Wang

21 Apr 2026

Dear Dr. Nasir,

Thank you for submitting your manuscript to PLOS ONE. After careful consideration, we feel that it has merit but does not fully meet PLOS ONE’s publication criteria as it currently stands. Therefore, we invite you to submit a revised version of the manuscript that addresses the points raised during the review process.

- Comments were received from only one reviewer.
- Please improve the quality of your manuscript based on the comments.

Please submit your revised manuscript by Jun 05 2026 11:59PM. If you will need more time than this to complete your revisions, please reply to this message or contact the journal office at plosone@plos.org. When you're ready to submit your revision, log on to https://www.editorialmanager.com/pone/ and select the 'Submissions Needing Revision' folder to locate your manuscript file.

- A letter that responds to each point raised by the academic editor and reviewer(s). You should upload this letter as a separate file labeled 'Response to Reviewers'.
- A marked-up copy of your manuscript that highlights changes made to the original version. You should upload this as a separate file labeled 'Revised Manuscript with Track Changes'.
- An unmarked version of your revised paper without tracked changes. You should upload this as a separate file labeled 'Manuscript'.

If you would like to make changes to your financial disclosure, please include your updated statement in your cover letter. Guidelines for resubmitting your figure files are available below the reviewer comments at the end of this letter.

If applicable, we recommend that you deposit your laboratory protocols in protocols.io to enhance the reproducibility of your results. Protocols.io assigns your protocol its own identifier (DOI) so that it can be cited independently in the future. For instructions see: https://journals.plos.org/plosone/s/submission-guidelines#loc-laboratory-protocols. Additionally, PLOS ONE offers an option for publishing peer-reviewed Lab Protocol articles, which describe protocols hosted on protocols.io. Read more information on sharing protocols at https://plos.org/protocols?utm_medium=editorial-email&utm_source=authorletters&utm_campaign=protocols.

As the corresponding author, your ORCID iD is verified in the submission system and will appear in the published article. PLOS supports the use of ORCID, and we encourage all coauthors to register for an ORCID iD and use it as well. Please encourage your coauthors to verify their ORCID iD within the submission system before final acceptance, as unverified ORCID iDs will not appear in the published article. *Only*  the individual author can complete the verification step; PLOS staff *cannot*  verify ORCID iDs on behalf of authors.

We look forward to receiving your revised manuscript.

Kind regards,

Jianguo Wang, PhD

Academic Editor

PLOS One

Journal Requirements:

When submitting your revision, we need you to address these additional requirements.

1. Please ensure that your manuscript meets PLOS ONE's style requirements, including those for file naming. The PLOS ONE style templates can be found at

https://journals.plos.org/plosone/s/file?id=wjVg/PLOSOne_formatting_sample_main_body.pdf and

https://journals.plos.org/plosone/s/file?id=ba62/PLOSOne_formatting_sample_title_authors_affiliations.pdf.

2. We note that your Data Availability Statement is currently as follows: [All relevant data are within the manuscript and its Supporting Information files. ]

Please confirm at this time whether or not your submission contains all raw data required to replicate the results of your study. Authors must share the “minimal data set” for their submission. PLOS defines the minimal data set to consist of the data required to replicate all study findings reported in the article, as well as related metadata and methods (https://journals.plos.org/plosone/s/data-availability#loc-minimal-data-set-definition).

For example, authors should submit the following data:

- The values behind the means, standard deviations and other measures reported;

- The values used to build graphs;

- The points extracted from images for analysis.

Authors do not need to submit their entire data set if only a portion of the data was used in the reported study.

If your submission does not contain these data, please either upload them as Supporting Information files or deposit them to a stable, public repository and provide us with the relevant URLs, DOIs, or accession numbers. For a list of recommended repositories, please see https://journals.plos.org/plosone/s/recommended-repositories.

If there are ethical or legal restrictions on sharing a de-identified data set, please explain them in detail (e.g., data contain potentially sensitive information, data are owned by a third-party organization, etc.) and who has imposed them (e.g., an ethics committee). Please also provide contact information for a data access committee, ethics committee, or other institutional body to which data requests may be sent. If data are owned by a third party, please indicate how others may request data access.

3. One of the noted authors is a group or consortium [The COVISTRESS network ]. In addition to naming the author group, please list the individual authors and affiliations within this group in the acknowledgments section of your manuscript. Please also indicate clearly a lead author for this group along with a contact email address.

4. Your ethics statement should only appear in the Methods section of your manuscript. If your ethics statement is written in any section besides the Methods, please delete it from any other section.

5. If the reviewer comments include a recommendation to cite specific previously published works, please review and evaluate these publications to determine whether they are relevant and should be cited. There is no requirement to cite these works unless the editor has indicated otherwise.

[Note: HTML markup is below. Please do not edit.]

Reviewers' comments:

Reviewer's Responses to Questions

**Comments to the Author**

1. Is the manuscript technically sound, and do the data support the conclusions?

Reviewer #1: Partly

**********

2. Has the statistical analysis been performed appropriately and rigorously?

Reviewer #1: I Don't Know

**********

3. Have the authors made all data underlying the findings in their manuscript fully available?

The PLOS Data policy

Reviewer #1: Yes

**********

4. Is the manuscript presented in an intelligible fashion and written in standard English?

Reviewer #1: Yes

**********

Reviewer #1: Dear authors

It has been a pleasure to have the opportunity to review the manuscript, “Teleworking as the main risk factor for sedentary behavior: the COVISTRESS’ international study”. As the debate on whether teleworking should or shouldn’t be allowed to continue rumbles on, this study provides an interesting insight into the potential health impacts of teleworking. It is clear that a lot of effort has gone into the study. Nonetheless, as you will see from my notes, I do have quite a few comments. These are not meant to dishearten. I hope they will lead to improvements in the clarity, readability, and reproducibility of the study, and following this I hope the study will receive a wider audience and further the discussions about teleworking – and how to improve worker health and wellbeing.

The remainder of my feedback is in the attachment.

Best wishes

**********

what does this mean?). If published, this will include your full peer review and any attached files.

If you choose “no”, your identity will remain anonymous but your review may still be made public.

**Do you want your identity to be public for this peer review?**  For information about this choice, including consent withdrawal, please see our Privacy Policy

Reviewer #1: No

**********

[NOTE: If reviewer comments were submitted as an attachment file, they will be attached to this email and accessible via the submission site. Please log into your account, locate the manuscript record, and check for the action link "View Attachments". If this link does not appear, there are no attachment files.]

To ensure your figures meet our technical requirements, please review our figure guidelines: https://journals.plos.org/plosone/s/figures

You may also use PLOS’s free figure tool, NAAS, to help you prepare publication quality figures: https://journals.plos.org/plosone/s/figures#loc-tools-for-figure-preparation.

NAAS will assess whether your figures meet our technical requirements by comparing each figure against our figure specifications.

Attachment Submitted filename: PLOS One telework comments to authors.docx

# Author comment: Author response to Decision Letter 1

**DOI:** 10.1371/journal.pone.0356764.r002

6 Jul 2026

Dear Reviewer,

Thank you for your time to review our manuscript entitled “Teleworking as the main risk factor for sedentary behavior: The COVISTRESS international study”.

We respond the questions for our article submission point by point as below:

Dear authors

It has been a pleasure to have the opportunity to review the manuscript, “Teleworking as the main risk factor for sedentary behavior: the COVISTRESS’ international study”. As the debate on whether teleworking should or shouldn’t be allowed to continue rumbles on, this study provides an interesting insight into the potential health impacts of teleworking. It is clear that a lot of effort has gone into the study. Nonetheless, as you will see from my notes below, I do have quite a few comments. These are not meant to dishearten. I hope they will lead to improvements in the clarity, readability, and reproducibility of the study, and following this I hope the study will receive a wider audience and further the discussions about teleworking – and how to improve worker health and wellbeing.

[REPLY] Thank you very much for your very helpful and detailed comments. We welcome all the suggestions and we are delighted to clarify all the unclear explanations in our manuscript. Thank you for all the time dedicated to provide us the valuable suggestions for better quality of our manuscript. Please find below point by point responses.

Introduction

1. Please would the authors consider providing a definition of teleworking. In case it’s useful, here is the link to Eurofound’s definition: https://www.eurofound.europa.eu/en/topics/teleworking

[REPLY] Thank you very much for your suggestion and provided link. We have integrated the definition of teleworking in our manuscript. The section Introduction now reads: “The European Foundation defines teleworking as an arrangement in which employees carry out their job tasks away from the usual workplace, typically outside the employer’s premises, using information and communication technologies (REF).”

2. To save the reader having to look up every reference, it would be helpful if the authors were more explicit in their storytelling. For example:

The authors state, “However, a major recent occupational change is related to teleworking, that could also be particularly impacting on sedentary behavior”. If the authors mean the Covid-19 pandemic then please will they say so?

[REPLY] Thank you very much for your suggestion. The section Introduction now reads: […] “A major recent occupational change concerns the rapid expansion of teleworking particularly since the COVID-19 pandemic (REF).” […]

The authors state, “A study quantifying the percentage of telework concluded that the workers with the highest percentage (76%-100% of time spent working) had the highest sedentary time compared with other groups (1%-25%, and 26%-50%).” Please will the authors state who the other groups were?

[REPLY] Thank you very much for your comment. We have added more information regarding the group with different percentage of telework. The section Introduction now reads: Workers with the highest proportion of telework (76%-100% of working time) have been shown to accumulate the highest sedentary time (305 mins/day) compared with other groups (235.2 mins/day for those being 1%-25% in telework, 243.5 mins/day for 26%-50% in telework, and 258.5 mins/day for 51%‐75% in telework) (REF).”

3. To improve clarity, please would the authors explain the following:

What do they mean by “weight” in their statement, “While aforementioned studies … only a few of them concomitantly assessed the weight of telework on sitting time in addition to …”? Whilst at this point I haven’t thoroughly read the manuscript in full, it appears they’ve reported effect size (using Cohen’s D, Cramer’s V, Odds Ratios, etc). Does their study assess weight or effect size?

[REPLY] Thank you very much for your comment. We would like to clarify what we mean with “weight” in this sentence. We wanted to explain that even though other studies have assessed the influence of telework on sitting time, only a few studies run multivariate models taking into account other variables, i.e., sociodemographic, occupational, and lifestyle variables. The sentence on Introduction section now reads: […] “However, most existing studies were conducted on a single country - three in Japan (REF) and one in the Netherland (REF) and only a minority used multivariate models adjusting simultaneously for sociodemographics, occupational, and lifestyle variables (REF). As a result, the independent contribution of telework relative to other determinants of sedentary behavior remains unclear.” […]

Do the authors mean one country, and if so, which country in their statement, “Moreover, those studies focused mainly on one country.”?

[REPLY] Thank you very much for your comment. The section Introduction now reads: […] “However, most existing studies were conducted on a single country - three in Japan (REF) and one in the Netherland (REF).” […]

What “other components” are relevant to the authors’ study in addition to the “many variables”, when they state, “As well as other components (26–28), sedentary behavior can be influenced by many variables (11,12,29)”?

[REPLY] Thank you very much for your comment. We apologize for the typo. The word should be “comportments” or “behavior”. We have rewritten this section. The section Introduction now reads: […] “Numerous sociodemographic, occupational, and lifestyle factors have been associated with sedentary behavior (REF), including older age (REF), women (REF, being single (REF) or living alone (REF), higher education (REF, living in the city vs rural (REF), and lower household income (REF), administrative workers and customer services occupations (REF), work-related stress (REF), smoking (REF), alcohol consumption (REF), cannabis use (REF), obesity (REF), and poor sleep (REF). Each of these factors contributes only partially to sedentary behavior, and their combined influence remains insufficiently explored.” […]

4. The authors state, “However, no studies took into account all those variables simultaneously in the statistical models investigating a link between sedentary time and telework.” Please would the authors explain more fully what the studies have accounted for, and what in their opinion has been missed? Could the lack of simultaneous inclusion of variables in previous studies be due to potential problems relating to the danger of overfitting, multicollinearity, biased causal estimates etc? Could it be due to data availability? Could the previous studies have decided for theoretical and/or statistical reasons not to include some variables?

[REPLY] Thank you very much for your comment. All those studies have accounted for many variables that influence sedentary time, but those studies did not assess the influence of telework. Therefore, we have the proof that numerous variables influence sedentary time … but the few studies on telework assessed just few of those variables. The section Introduction now reads: […] “However, most existing studies were conducted on a single country - three in Japan (REF) and one in the Netherland (REF) and only a minority used multivariate models adjusting simultaneously for sociodemographics, occupational, and lifestyle variables (REF). As a result, the independent contribution of telework relative to other determinants of sedentary behavior remains unclear. Furthermore, previous studies generally categorized sitting time using thresholds such as 8 h/day or, more rarely, 11–12 h/day (REF). To our knowledge, no study has examined extreme sitting time, defined as >14 h/day, largely because available sample sizes were insufficient to capture such rare but clinically relevant behaviors. Identifying this subgroup is important, as extremely prolonged sitting may reflect distinct behavioral patterns and may carry disproportionately high health risks. […]

Methods: Study Design

5. Whilst the authors do state that “Participation was entirely voluntary and anonymous”, it would be helpful if they would also explain if participants are self-selecting and if so, how this might bias the results (and to address this too in the section on limitations).

[REPLY] Thank you very much for your comment. The section Methods Study Design now reads: “The COVISTRESS study is an international, cross-sectional online survey assessing various sociodemographic, occupational, and lifestyle behavior using a self-reported questionnaire. […] Participation was entirely voluntary and anonymous. Participants were self‑selecting.” […]

The Limitations section now reads: “The questionnaire was anonymous (REF) and participants were self‑selecting that may create bias, such as individuals who voluntarily participated may have stronger interest, awareness, or personal experience with the topic, leading to over-representation of certain behaviors. In addition, as this is a fully online questionnaire, it could attract more screen user participants (REF), precluding the population that are not familiar with internet or screen use. Nonetheless, internet use is nowadays widely common (REF).”

Methods: Participants and also Results: Participants

6. The authors state, “We included all participants who answered their sitting time (Figure 1).” Please would the authors double check the data shown in this figure? It shows the number of participants was reduced from 7064 to 6653 by excluding 1333 who did not respond to the question about sitting time. 7064 minus 1333 equals 5,731 not 6653.

[REPLY] Thank you very much for your very detailed findings. The number of participants answered the “COVISTRESS questionnaire” were 7,064. Among them, only 6,653 participants who answered the questions “number of hours of sitting per day”. So, excluded participants were n=411. We apologize for the mistake. We have revised Figure 1 (Flow chart).

7. Figure 1 shows there were 2,904 participants who responded to the question about telework. As the aim of the study was to take into account all the variables (which can influence sedentary behavior) “simultaneously in the statistical models investigating a link between sedentary time and telework” was the final number of participants in the study 2,904 or did the authors do some form of missing data imputation?

[REPLY] Thank you very much for your comment. There were 2,904 participants who responded both to the question about telework and sedentary time. We did not perform multiple imputation because the objective was to investigate associations between sedentary time and telework while simultaneously adjusting for numerous potential confounders. Imputing all explanatory variables would have required additional assumptions regarding the missing-data mechanism and could have introduced additional uncertainty into the multivariable models. We therefore adopted a complete-case approach. In addition, to strengthen the robustness of our findings, we performed several prespecified sensitivity analyses, which all led to consistent conclusions. The last sentence of the “Statistical analyses - Methods section” now reads: […] “We further performed several prespecified sensitivity analyses to strengthen the robustness of our findings. We studied the relationship between covariables to evaluate the impact by removing the less answered variables on multivariate regression analysis, i.e. marital status, parenthood, number of inhabitants, and work addiction (Appendix 5). We conducted step by step regression analyses by adding variables one by one in addition to telework to verify the consistency of the association between sitting time and telework (Appendix 6). A complete-case approach with multivariate model using missing data as a specific class for each variable was also performed (Appendix 7). Furthermore, in order to search for the pattern of missing data, we conducted assessment of non‑response bias through comparison of complete and incomplete respondents (Appendix 8). Statistical significance was marked by p-value ≤0.05. There was no imputation of missing data.”

We added the following sentence in the Results section: “Among the 7064 respondents who answered the COVISTRESS sedentary questionnaire in 2023 and 2024, 6653 answered the sitting time item and were included. Among them, 2904 responded both to sitting time and telework items, and 1417 responded to all questions without any missing data (Figure 1).” Figure 1 has been updated.

We now present a new Appendix 6 (step by step regression analyses) that shows the number of subjects within each model.

8. Figure 1 also includes the number of responses per variable. What was the pattern of missing data? Was there any imputation? What was the final analytical sample?

[REPLY] Thank you very much for your comment. The “Statistical analyses - Methods section” now reads: […] “Furthermore, in order to search for the pattern of missing data, we conducted assessment of non‑response bias through comparison of complete and incomplete respondents (Appendix 8). […] There was no imputation of missing data.”

The final analytical sample has been added in Figure 1 (see above) as well as in the Results section: “Among the 7064 respondents who answered the COVISTRESS sedentary questionnaire in 2023 and 2024, 6653 answered the sitting time item and were included. Among them, 2904 responded both to sitting time and telework items, and 1417 responded to all questions without any missing data (Figure 1).”

We also now add Appendix 8 (assessment of non‑response bias through comparison of complete and incomplete respondents).

The Result: Sensitivity analysis now reads: […] “ We further studied the relationship between covariables to evaluate the impact by removing the less answered variables on multivariate regression analysis (i.e. marital status, parenthood, number of inhabitants, and work addiction), the number of observations increased by >20% (n=1740) and demonstrated similar results (Appendix 5). We also performed step by step regression analyses by adding variables one by one in addition to telework, demonstrating the consistency of the association between sitting time and telework (Appendix 6). The association remained also consistent by coding all missing data as variables with NA handling to a specific class for each variable. To note, coding missing data as a distinct category produced narrower confidence intervals, indicating greater precision and supporting the stability of the estimated association (Appendix 7). With a cut-off at ≥14 hours, we increase the number of workers sitting more than 14 hours by 50 people (from 108 sitting >14 hours/d to 158 sitting ≥14 hours/d). Using this cutoff as a sensitivity analysis also give similar results for all analyses. Furthermore, the assessment of non‑response bias through comparison of complete and incomplete respondents showed that the respondents with missing data did not differ from those who fully answered all items, except for sleep quality (Cramer’s V=0.173) (Appendix 8).

The limitations section now reads: “Some questions were less answered – especially sensitive questions such as BMI, age, mental health condition, or alcohol/smoking/cannabis uses. However, our sensitivity analysis showed that respondents with missing data did not differ from those who fully answered all items except for variable sleep quality.” […]

9. The authors state that the “teleworking variables were applicable only to this subgroup”. What exactly were the teleworking variables – what were respondents asked?

[REPLY] Thank you very much for your comment and correcting typos / unclear sentences. The sentence now reads: […] “However, analyses focused on employed participants, as teleworking is only applicable for workers.”

Methods: Outcomes Instrument Survey

10. The inclusion of the word “Outcomes” suggest that this will focus only on the outcome – sedentary behavior. However, this section covers more than that. For reader clarity, it might be helpful to replace “Outcomes Instrument Survey” with something along the lines of “Survey variables”; “Main outcomes” with “exposure and outcome variables” or “dependent and independent variables”; and “secondary outcomes” with “covariates”.

[REPLY

Attachment Submitted filename: COVISTRESS-sedentary_ResponseToReviewers_PLOS One.docx

# Peer review: Decision Letter 1

**DOI:** 10.1371/journal.pone.0356764.r003

**Authors:** Jianguo Wang

10 Aug 2026

Teleworking as the main risk factor for sedentary behavior: the COVISTRESS international study

PONE-D-25-59938R1

Dear Dr. Nasir,

We’re pleased to inform you that your manuscript has been judged scientifically suitable for publication and will be formally accepted for publication once it meets all outstanding technical requirements.

Within one week, you’ll receive an e-mail detailing the required amendments. When these have been addressed, you’ll receive a formal acceptance letter and your manuscript will be scheduled for publication.

An invoice will be generated when your article is formally accepted. Please note, if your institution has a publishing partnership with PLOS and your article meets the relevant criteria, all or part of your publication costs will be covered. Please make sure your user information is up-to-date by logging into Editorial Manager at Editorial Manager® and clicking the ‘Update My Information' link at the top of the page. For questions related to billing, please contact billing support.

If your institution or institutions have a press office, please notify them about your upcoming paper to help maximize its impact. If they’ll be preparing press materials, please inform our press team as soon as possible -- no later than 48 hours after receiving the formal acceptance. Your manuscript will remain under strict press embargo until 2 pm Eastern Time on the date of publication. For more information, please contact onepress@plos.org.

Kind regards,

Jianguo Wang, PhD

Academic Editor

PLOS One

Additional Editor Comments (optional):

Reviewers' comments:

Reviewer's Responses to Questions

**Comments to the Author**

Reviewer #1: All comments have been addressed

**********

2. Is the manuscript technically sound, and do the data support the conclusions?

Reviewer #1: (No Response)

**********

3. Has the statistical analysis been performed appropriately and rigorously?

Reviewer #1: (No Response)

**********

4. Have the authors made all data underlying the findings in their manuscript fully available?

The PLOS Data policy

Reviewer #1: (No Response)

**********

5. Is the manuscript presented in an intelligible fashion and written in standard English?

Reviewer #1: (No Response)

**********

Reviewer #1: Dear authors,

Many thanks for your comprehensive response to my review, and for sharing your earnest revisions to the manuscript. I find it much improved - in terms of clarity, readability, and potential reproducibility of the study, I wish you success.

Best wishes.

**********

what does this mean?). If published, this will include your full peer review and any attached files.

If you choose “no”, your identity will remain anonymous but your review may still be made public.

**Do you want your identity to be public for this peer review?**  For information about this choice, including consent withdrawal, please see our Privacy Policy

Reviewer #1: No

**********

# Editor report: Acceptance letter

**DOI:** 10.1371/journal.pone.0356764.r004

**Authors:** Jianguo Wang

PONE-D-25-59938R1

PLOS One

Dear Dr. Nasir,

I'm pleased to inform you that your manuscript has been deemed suitable for publication in PLOS One. Congratulations! Your manuscript is now being handed over to our production team.

At this stage, our production department will prepare your paper for publication. This includes ensuring the following:

* All references, tables, and figures are properly cited

* All relevant supporting information is included in the manuscript submission,

* There are no issues that prevent the paper from being properly typeset

You will receive further instructions from the production team, including instructions on how to review your proof when it is ready. Please keep in mind that we are working through a large volume of accepted articles, so please give us a few days to review your paper and let you know the next and final steps.

Lastly, if your institution or institutions have a press office, please let them know about your upcoming paper now to help maximize its impact. If they'll be preparing press materials, please inform our press team within the next 48 hours. Your manuscript will remain under strict press embargo until 2 pm Eastern Time on the date of publication. For more information, please contact onepress@plos.org.

You will receive an invoice from PLOS for your publication fee after your manuscript has reached the completed accept phase. If you receive an email requesting payment before acceptance or for any other service, this may be a phishing scheme. Learn how to identify phishing emails and protect your accounts at https://explore.plos.org/phishing.

If we can help with anything else, please email us at customercare@plos.org.

Thank you for submitting your work to PLOS One and supporting open access.

Kind regards,

PLOS One Editorial Office Staff

on behalf of

Dr. Jianguo Wang

Academic Editor

PLOS One
